# Supplementary material for: Addressing a Gap in Medical School Training: Identifying and Caring for Human Trafficking Survivors Using Trauma-Informed Care
Source: MedEdPORTAL. 2023 Mar 14;19:11304. doi: 10.15766/mep_2374-8265.11304 (PMC10011204; doi:10.15766/mep_2374-8265.11304)
Supplement: Supplementary file 1 — Didactic Lecture.pptxFacilitation Guide.docxStudent Worksheet Without Answers.docxStudent Worksheet With Suggested Answers.docxTool Kit.docxPre- and Postsession Survey Questions.docxExtra Scenarios.docx [file mep_2374-8265.11304-s001.zip › A. Didactic Lecture.pptx]

## Slide 1
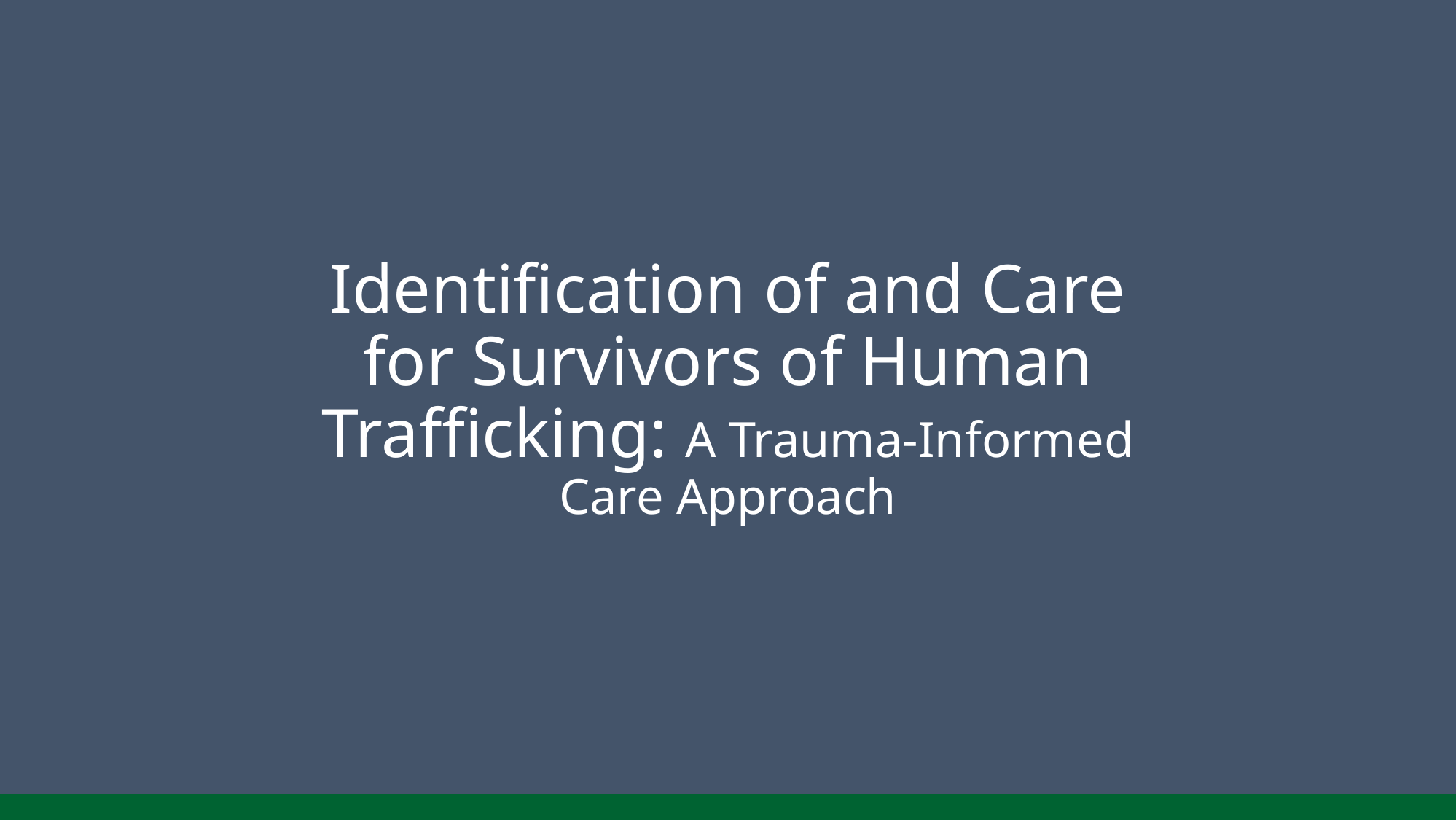

# Identification of and Care for Survivors of Human Trafficking: A Trauma-Informed Care Approach

## Slide 2
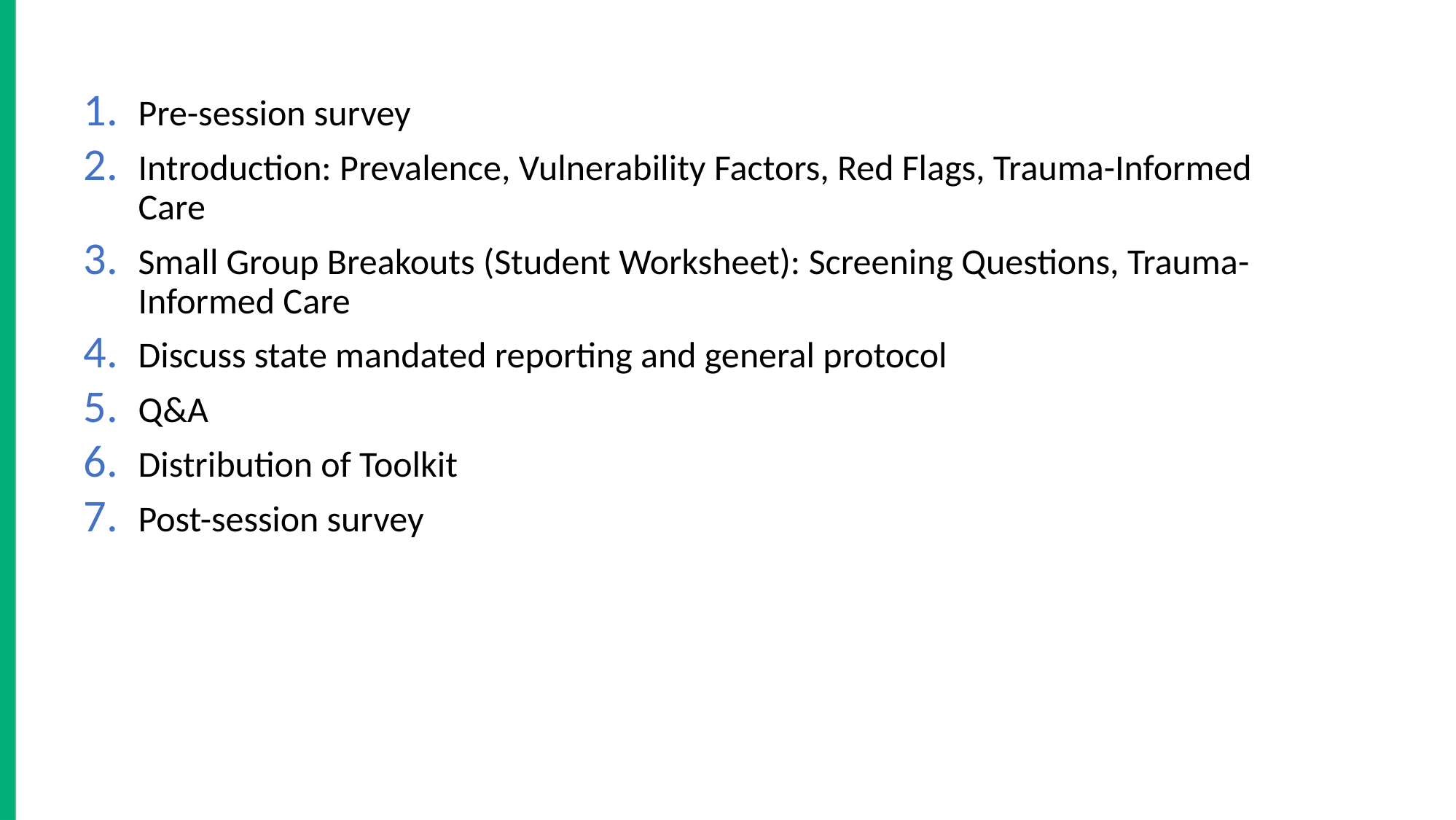

Pre-session survey
Introduction: Prevalence, Vulnerability Factors, Red Flags, Trauma-Informed Care
Small Group Breakouts (Student Worksheet): Screening Questions, Trauma-Informed Care
Discuss state mandated reporting and general protocol
Q&A
Distribution of Toolkit
Post-session survey

## Slide 3
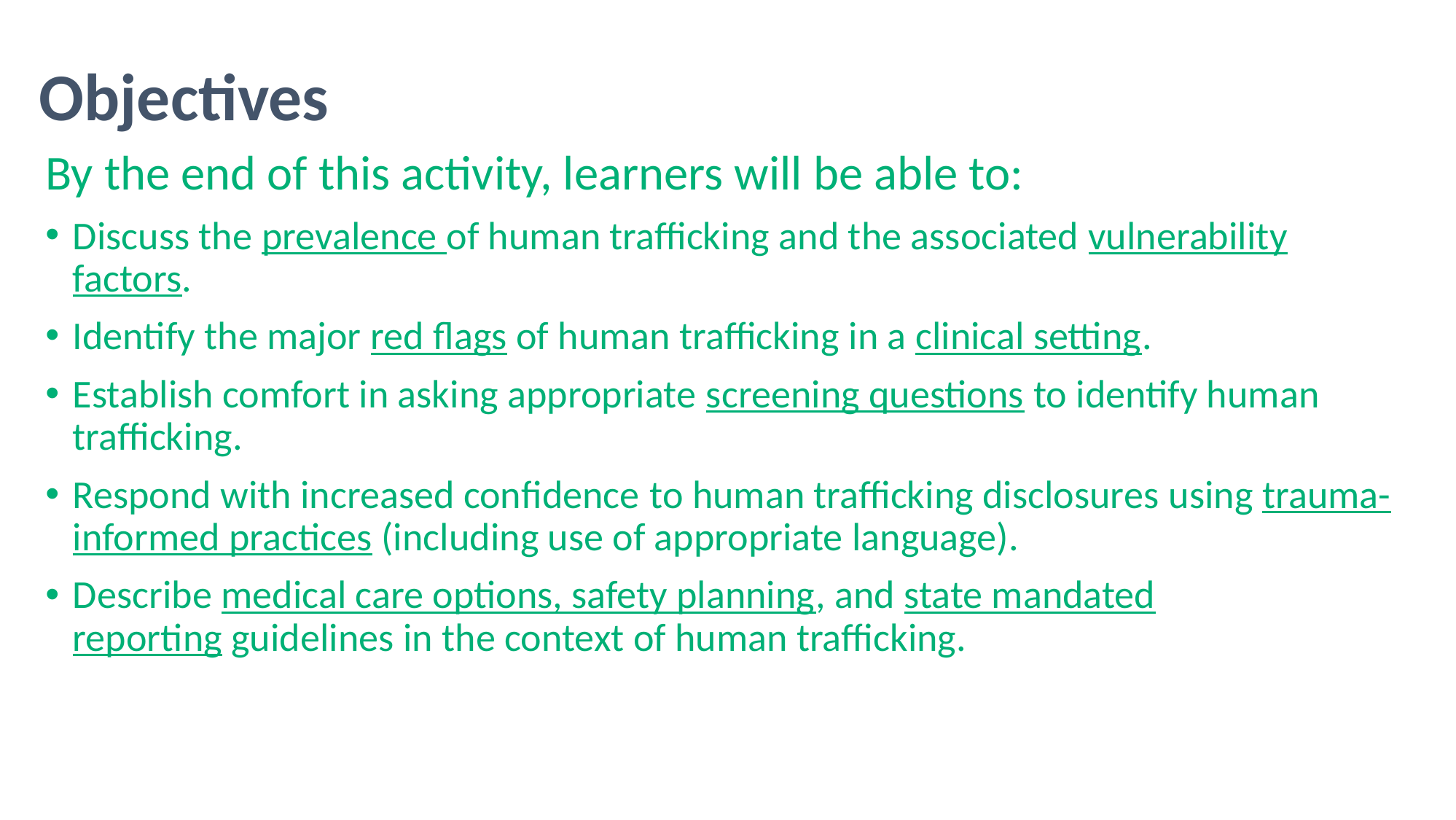

Objectives
By the end of this activity, learners will be able to:​
Discuss the prevalence of human trafficking and the associated vulnerability factors.​
Identify the major red flags of human trafficking in a clinical setting.​
Establish comfort in asking appropriate screening questions to identify human trafficking.​
Respond with increased confidence to human trafficking disclosures using trauma-informed practices (including use of appropriate language).​
Describe medical care options, safety planning, and state mandated reporting guidelines in the context of human trafficking.​

## Slide 4
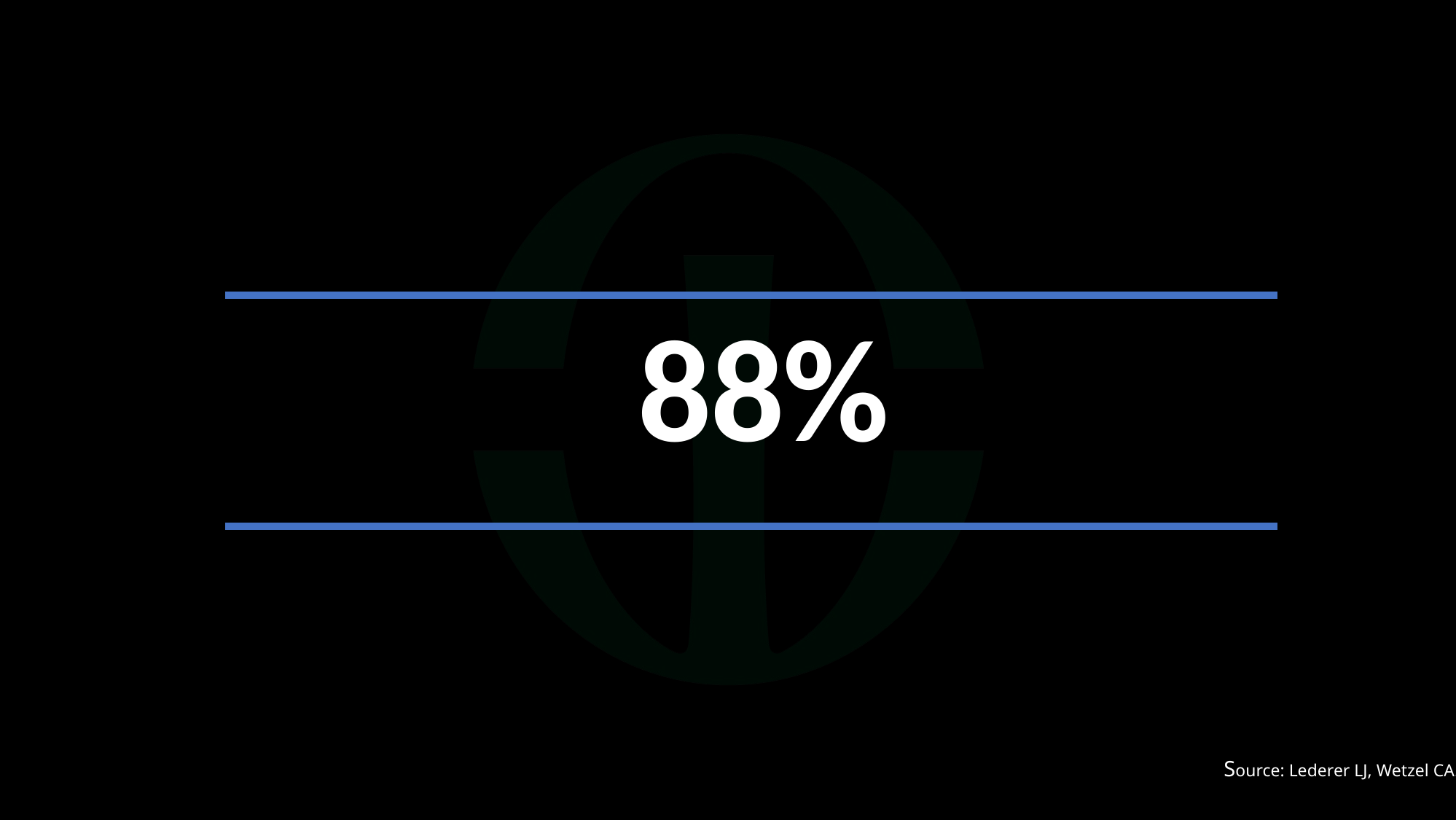

88%
Source: Lederer LJ, Wetzel CA.

## Slide 5
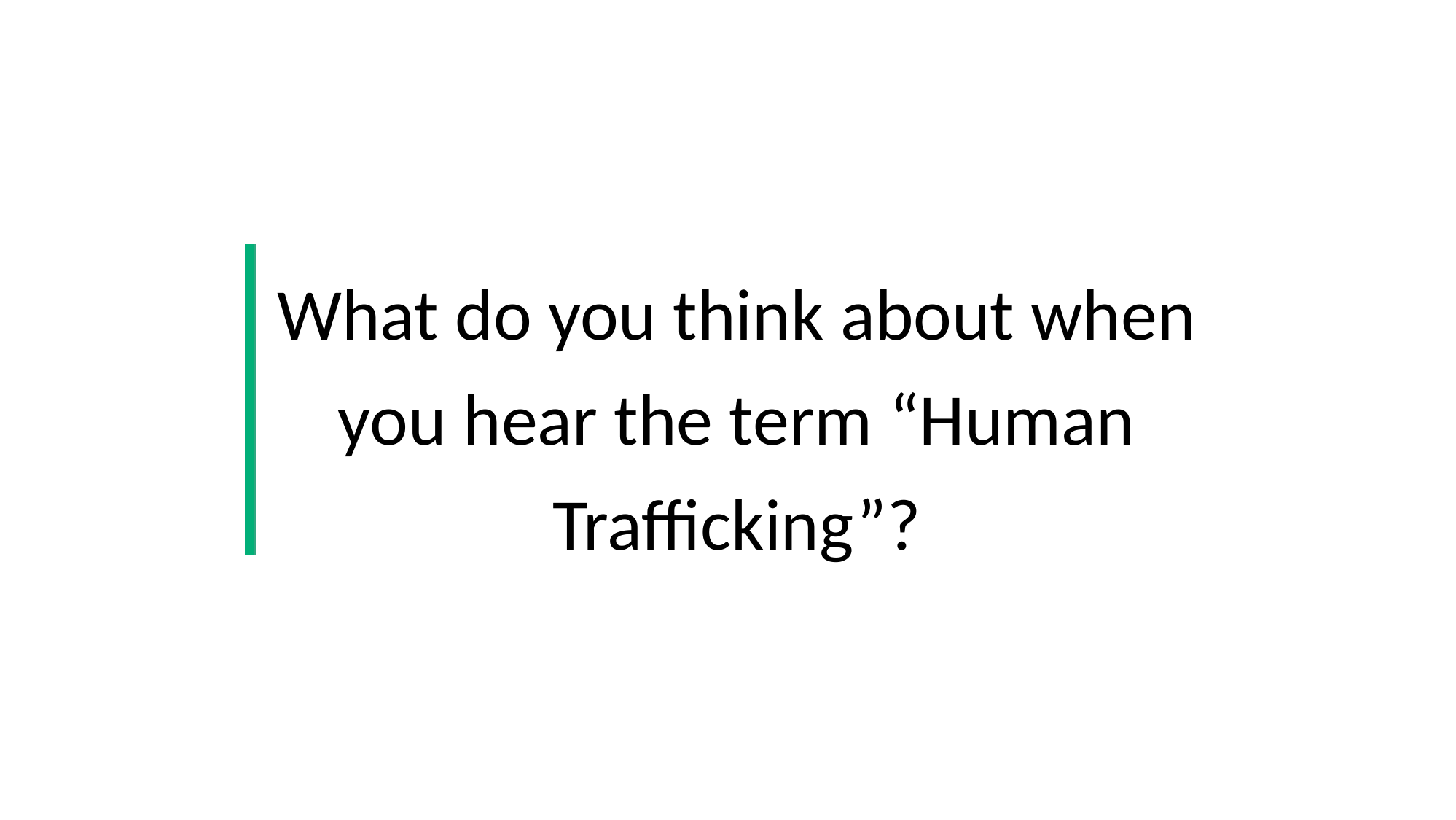

What do you think about when you hear the term “Human Trafficking”?

## Slide 6
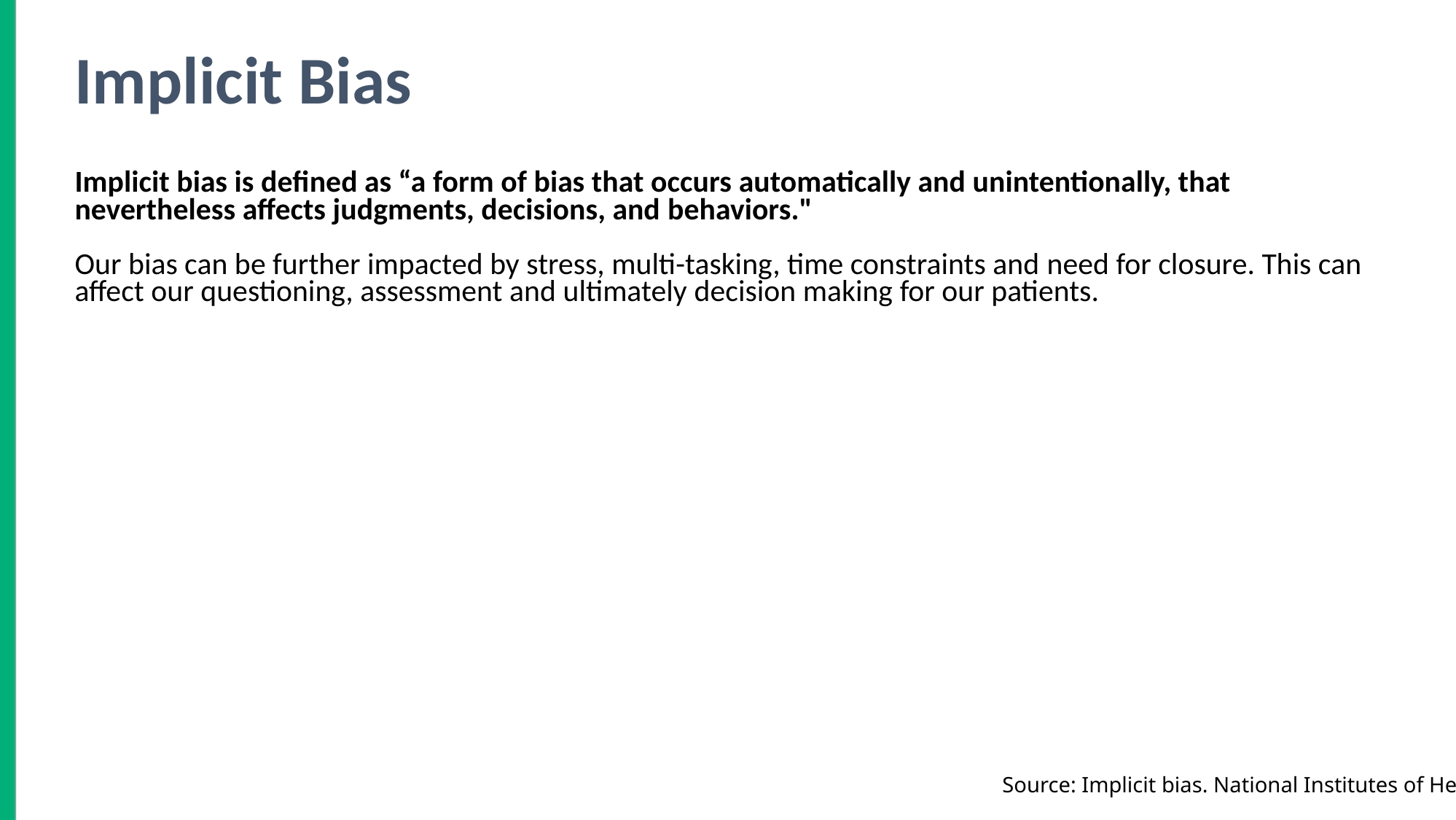

Implicit Bias
Implicit bias is defined as “a form of bias that occurs automatically and unintentionally, that nevertheless affects judgments, decisions, and behaviors."
Our bias can be further impacted by stress, multi-tasking, time constraints and need for closure. This can affect our questioning, assessment and ultimately decision making for our patients.
Source: Implicit bias. National Institutes of Health.

## Slide 7
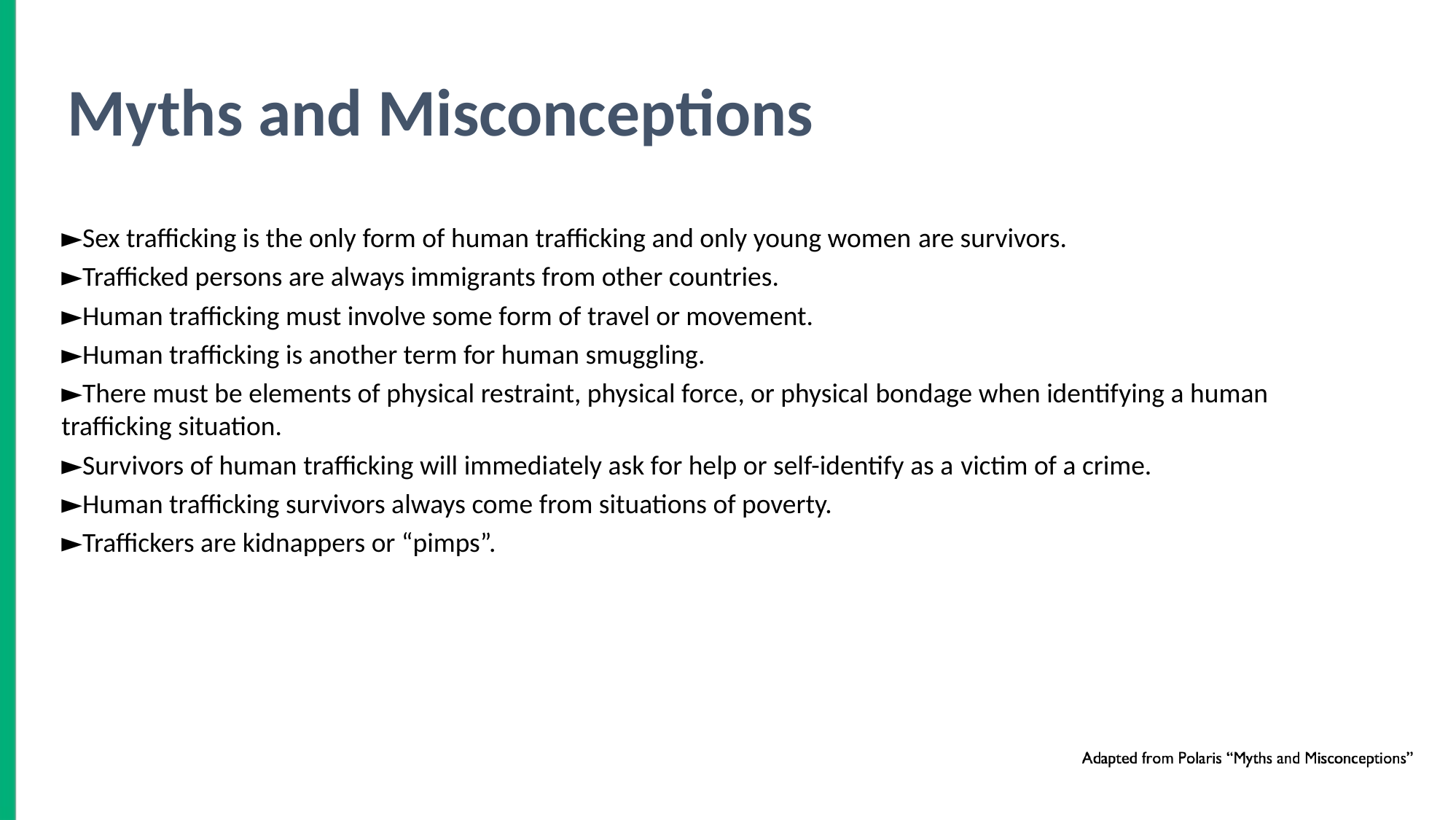

Myths and Misconceptions
►Sex trafficking is the only form of human trafficking and only young women are survivors.
►Trafficked persons are always immigrants from other countries.
►Human trafficking must involve some form of travel or movement.
►Human trafficking is another term for human smuggling.
►There must be elements of physical restraint, physical force, or physical bondage when identifying a human trafficking situation.
►Survivors of human trafficking will immediately ask for help or self-identify as a victim of a crime.
►Human trafficking survivors always come from situations of poverty.
►Traffickers are kidnappers or “pimps”.

## Slide 8
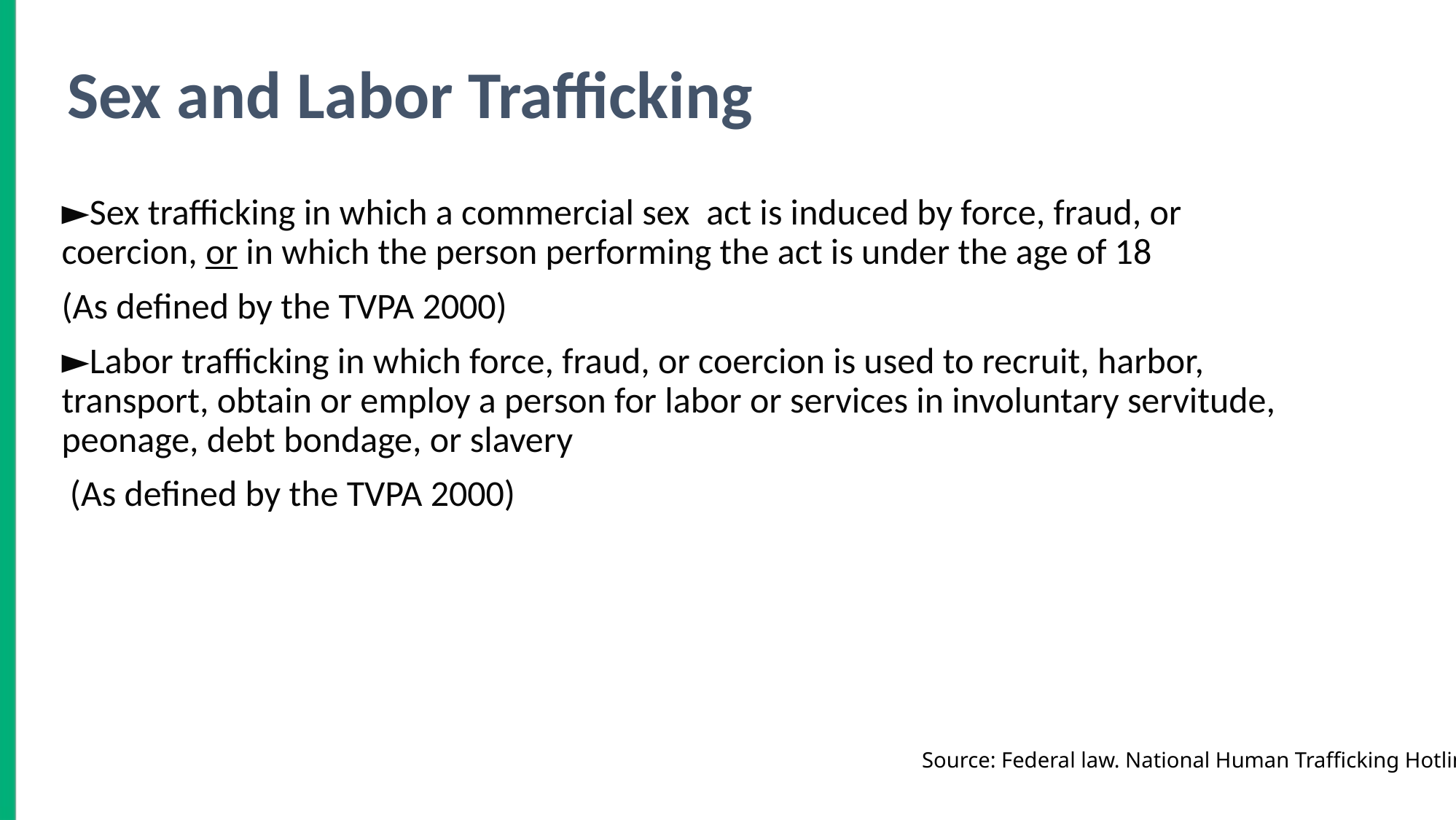

Sex and Labor Trafficking
►Sex trafficking in which a commercial sex  act is induced by force, fraud, or coercion, or in which the person performing the act is under the age of 18
(As defined by the TVPA 2000)
►Labor trafficking in which force, fraud, or coercion is used to recruit, harbor, transport, obtain or employ a person for labor or services in involuntary servitude, peonage, debt bondage, or slavery
 (As defined by the TVPA 2000)
Source: Federal law. National Human Trafficking Hotline

## Slide 9
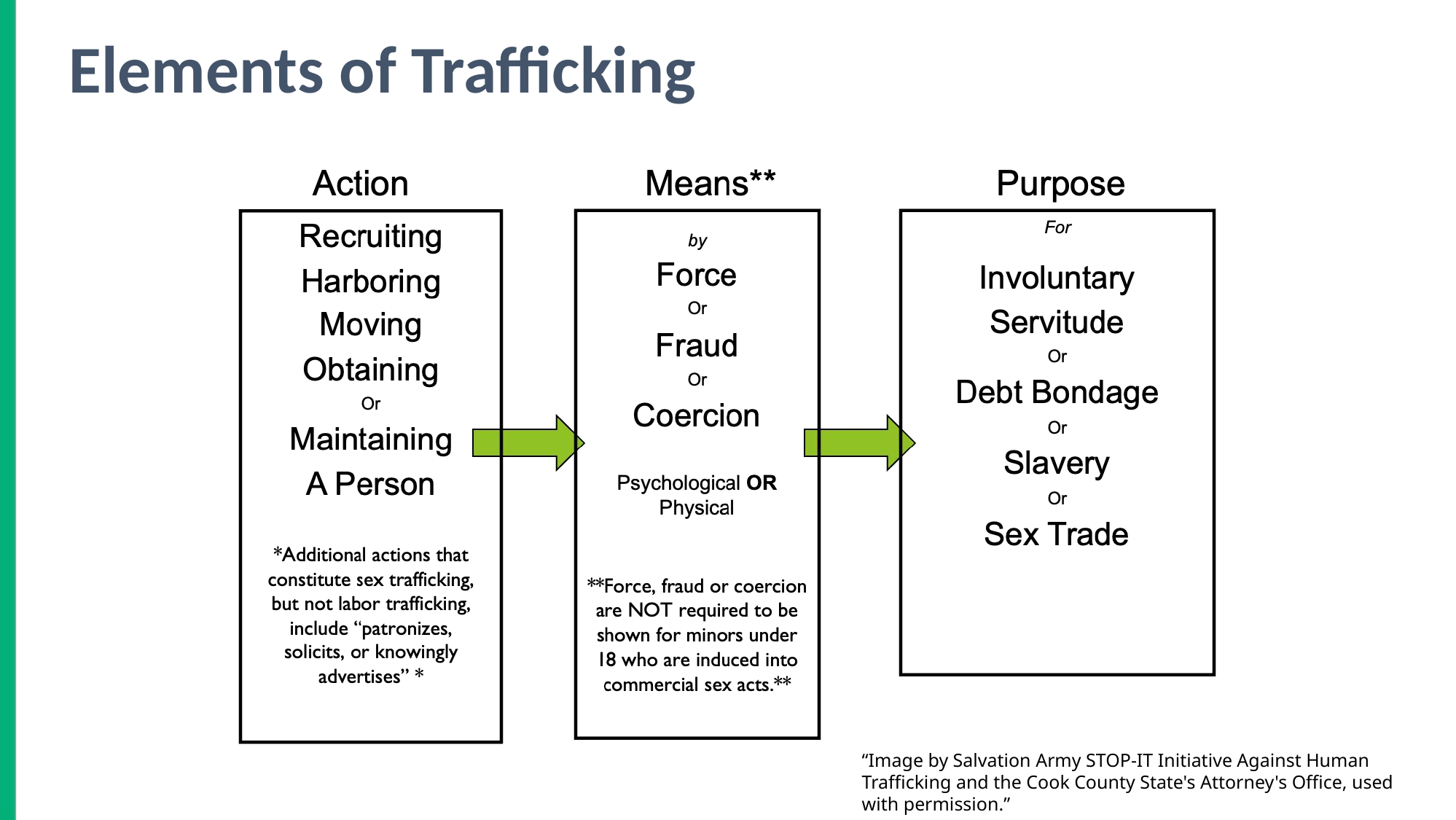

Elements of Trafficking
“Image by Salvation Army STOP-IT Initiative Against Human Trafficking and the Cook County State's Attorney's Office, used with permission.”

## Slide 10
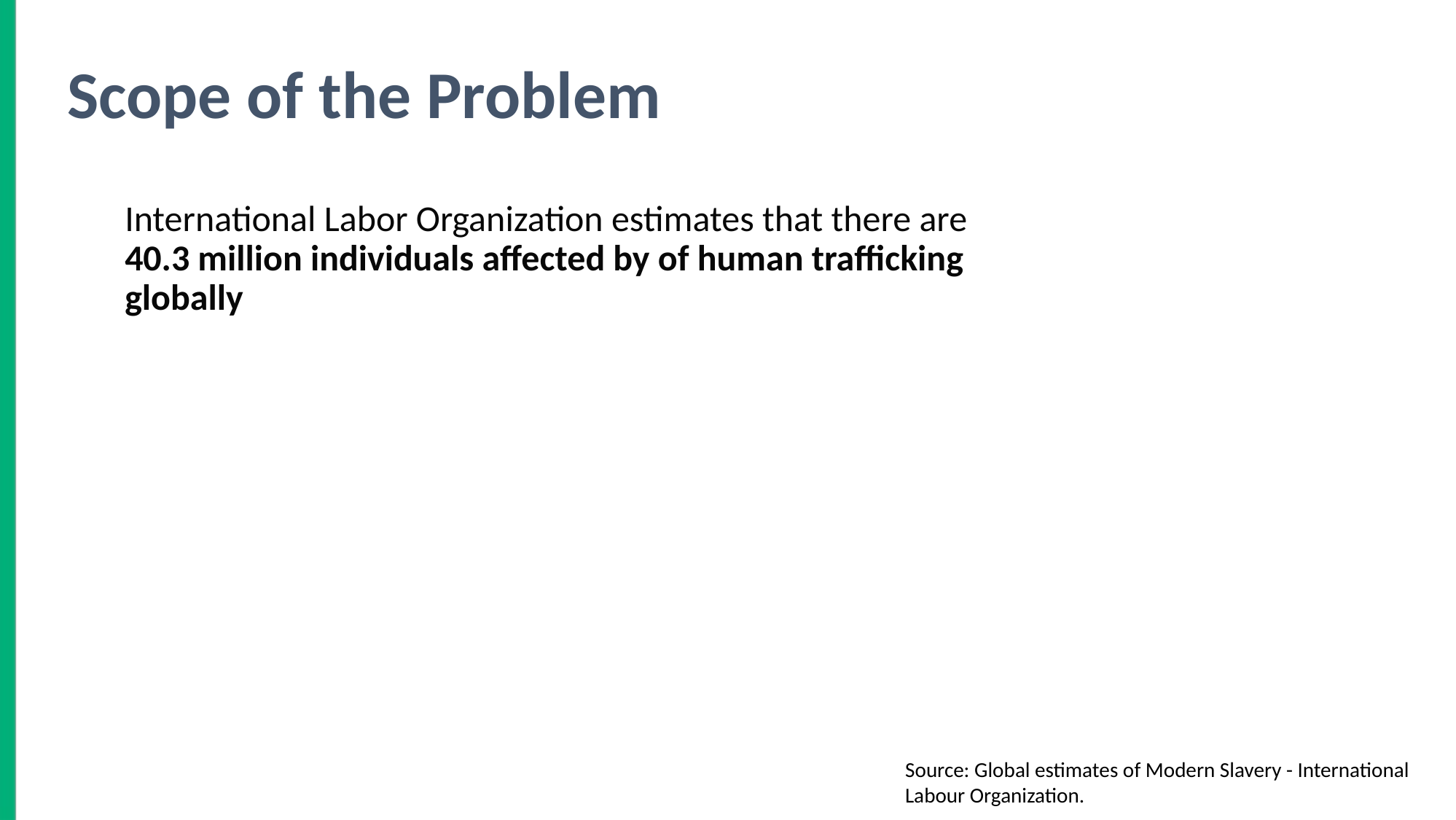

Scope of the Problem
International Labor Organization estimates that there are 40.3 million individuals affected by of human trafficking globally
Source: Global estimates of Modern Slavery - International Labour Organization.

## Slide 11
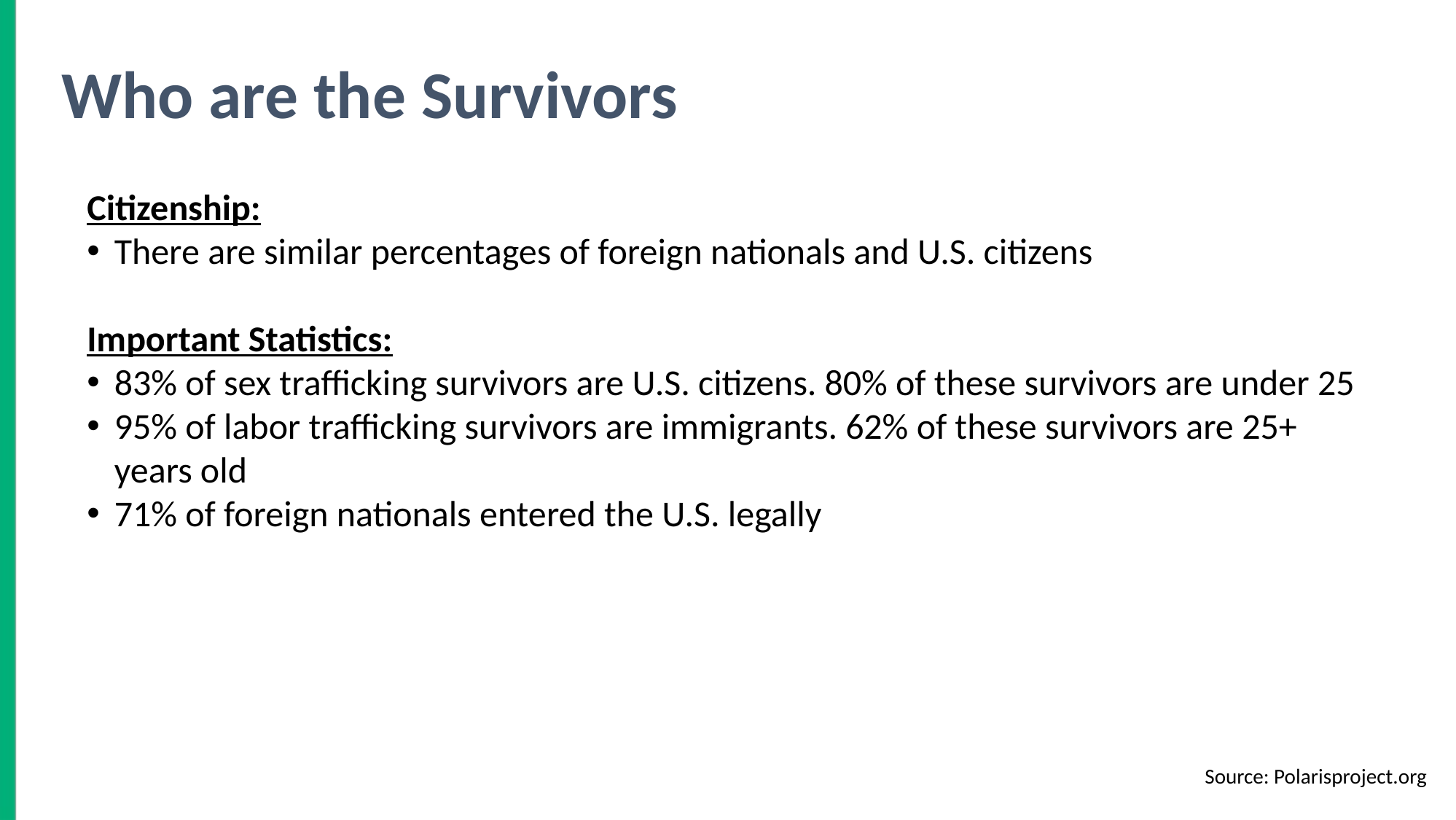

Who are the Survivors
Citizenship:
There are similar percentages of foreign nationals and U.S. citizens
Important Statistics:
83% of sex trafficking survivors are U.S. citizens. 80% of these survivors are under 25
95% of labor trafficking survivors are immigrants. 62% of these survivors are 25+ years old
71% of foreign nationals entered the U.S. legally
Source: Polarisproject.org​

## Slide 12
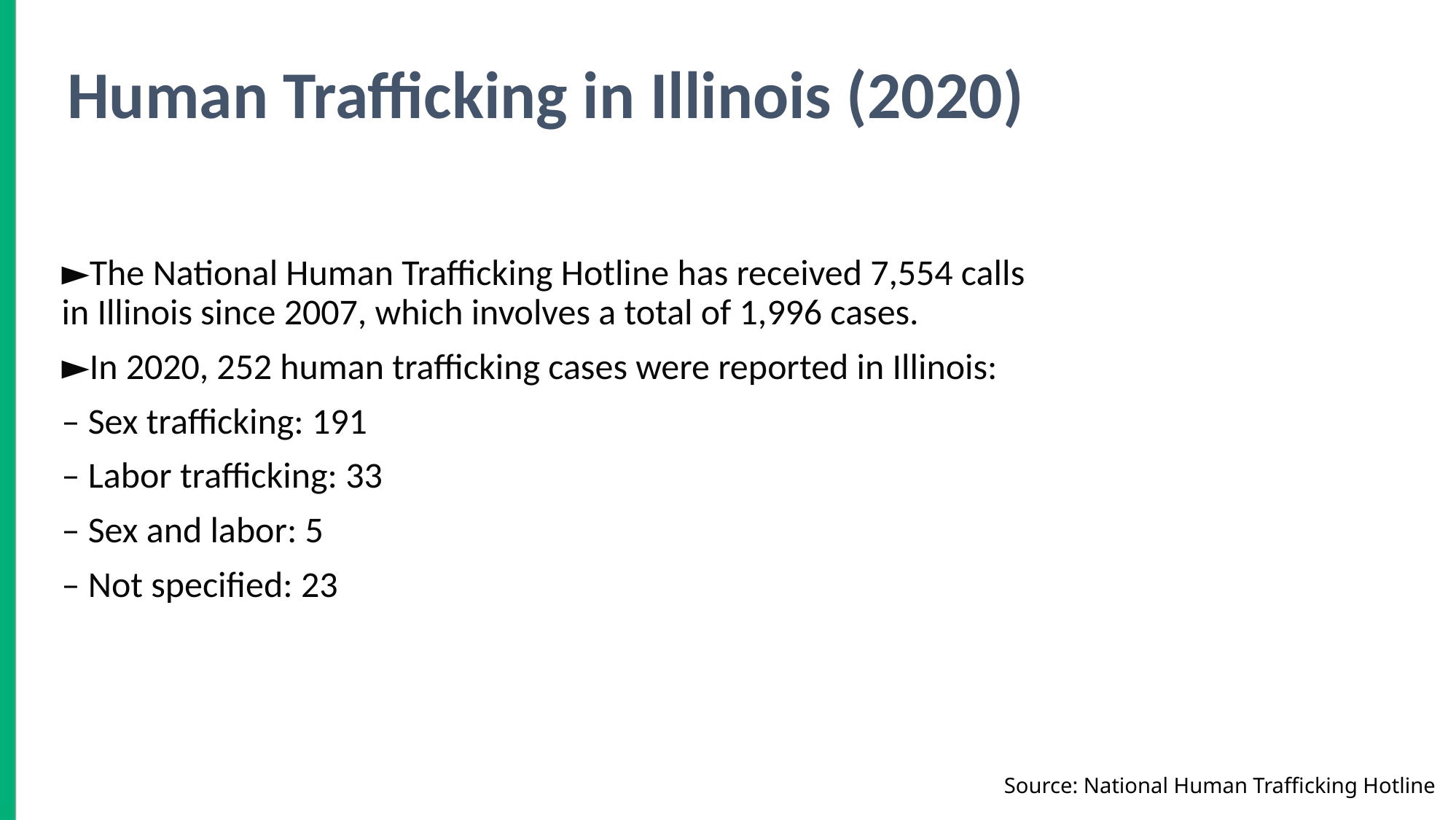

Human Trafficking in Illinois (2020)
►The National Human Trafficking Hotline has received 7,554 calls in Illinois since 2007, which involves a total of 1,996 cases.
►In 2020, 252 human trafficking cases were reported in Illinois:
– Sex trafficking: 191
– Labor trafficking: 33
– Sex and labor: 5
– Not specified: 23
Source: National Human Trafficking Hotline

## Slide 13
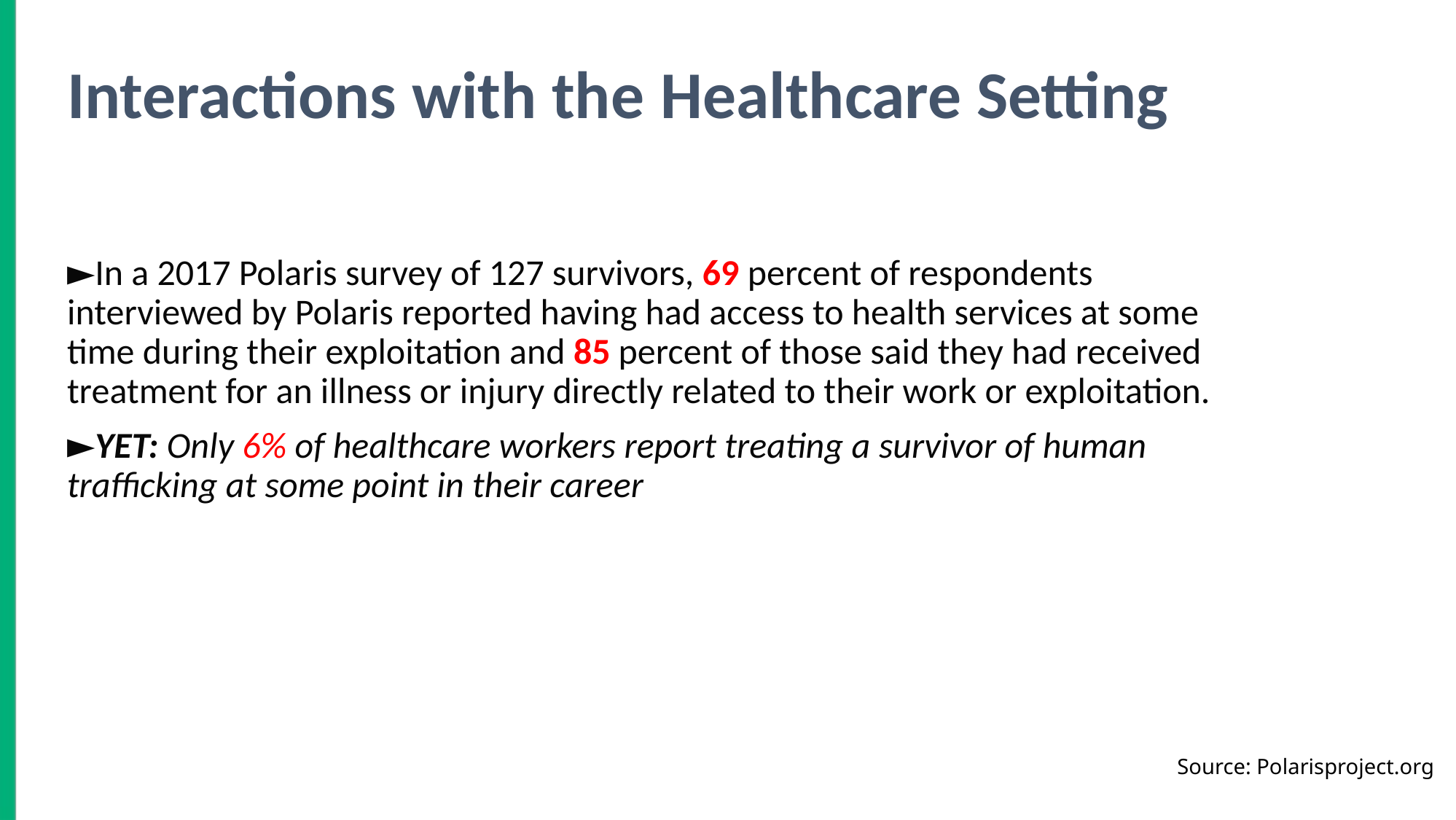

Interactions with the Healthcare Setting
►In a 2017 Polaris survey of 127 survivors, 69 percent of respondents interviewed by Polaris reported having had access to health services at some time during their exploitation and 85 percent of those said they had received treatment for an illness or injury directly related to their work or exploitation.
►YET: Only 6% of healthcare workers report treating a survivor of human trafficking at some point in their career
Source: Polarisproject.org

## Slide 14
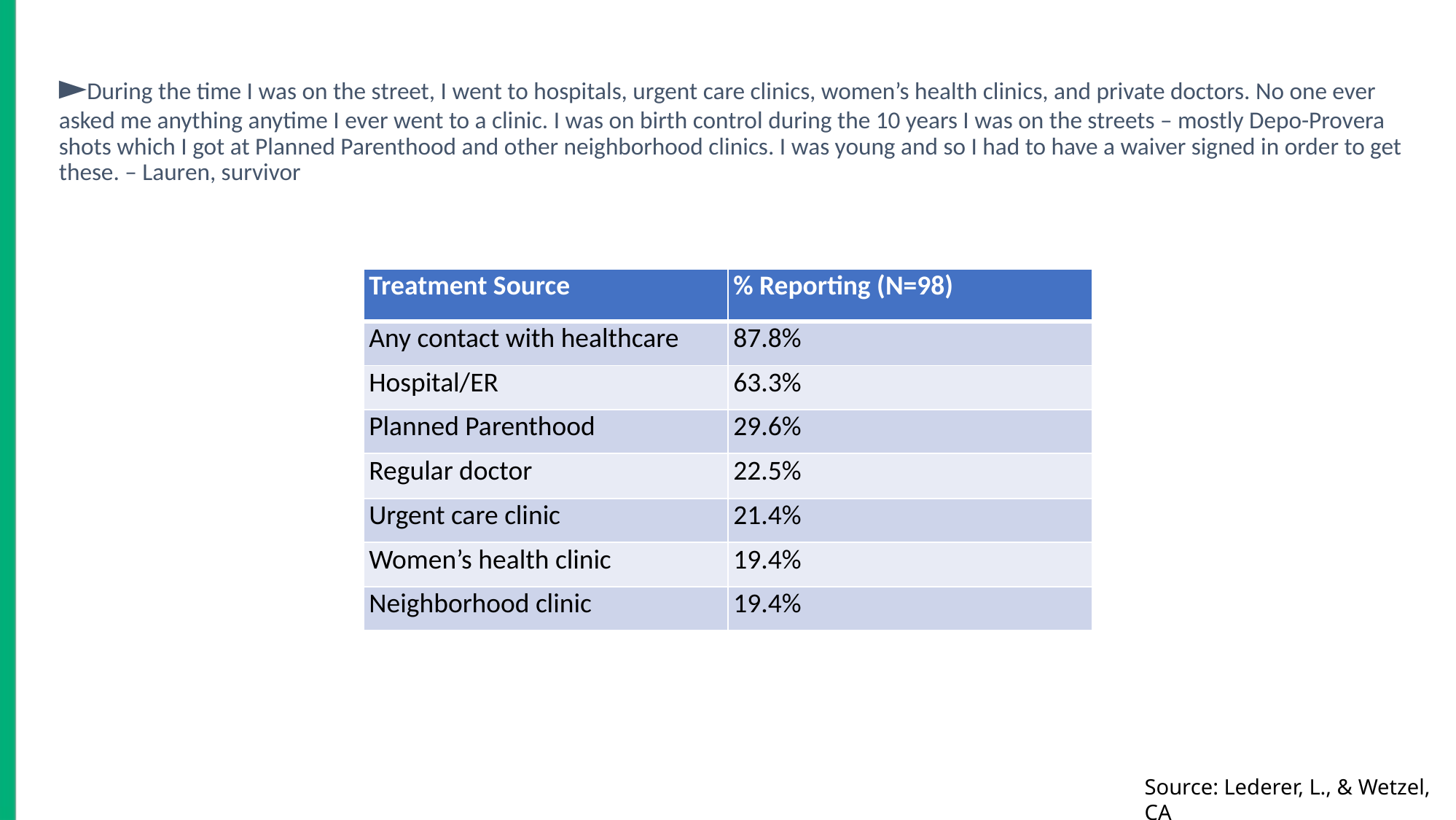

►During the time I was on the street, I went to hospitals, urgent care clinics, women’s health clinics, and private doctors. No one ever asked me anything anytime I ever went to a clinic. I was on birth control during the 10 years I was on the streets – mostly Depo-Provera shots which I got at Planned Parenthood and other neighborhood clinics. I was young and so I had to have a waiver signed in order to get these. – Lauren, survivor
| Treatment Source | % Reporting (N=98) |
| --- | --- |
| Any contact with healthcare | 87.8% |
| Hospital/ER | 63.3% |
| Planned Parenthood | 29.6% |
| Regular doctor | 22.5% |
| Urgent care clinic | 21.4% |
| Women’s health clinic | 19.4% |
| Neighborhood clinic | 19.4% |
Source: Lederer, L., & Wetzel, CA

## Slide 15
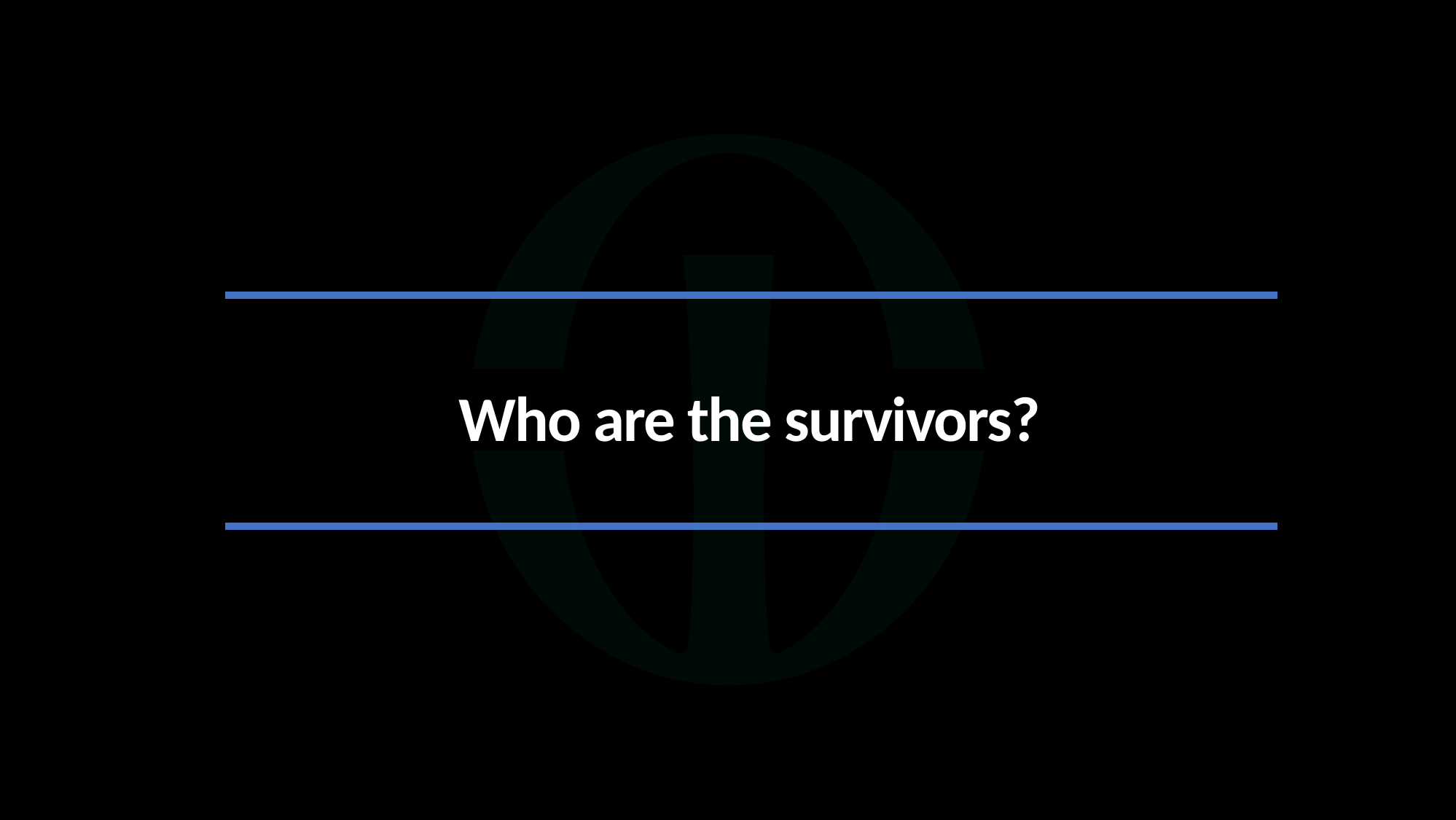

Who are the survivors?

## Slide 16
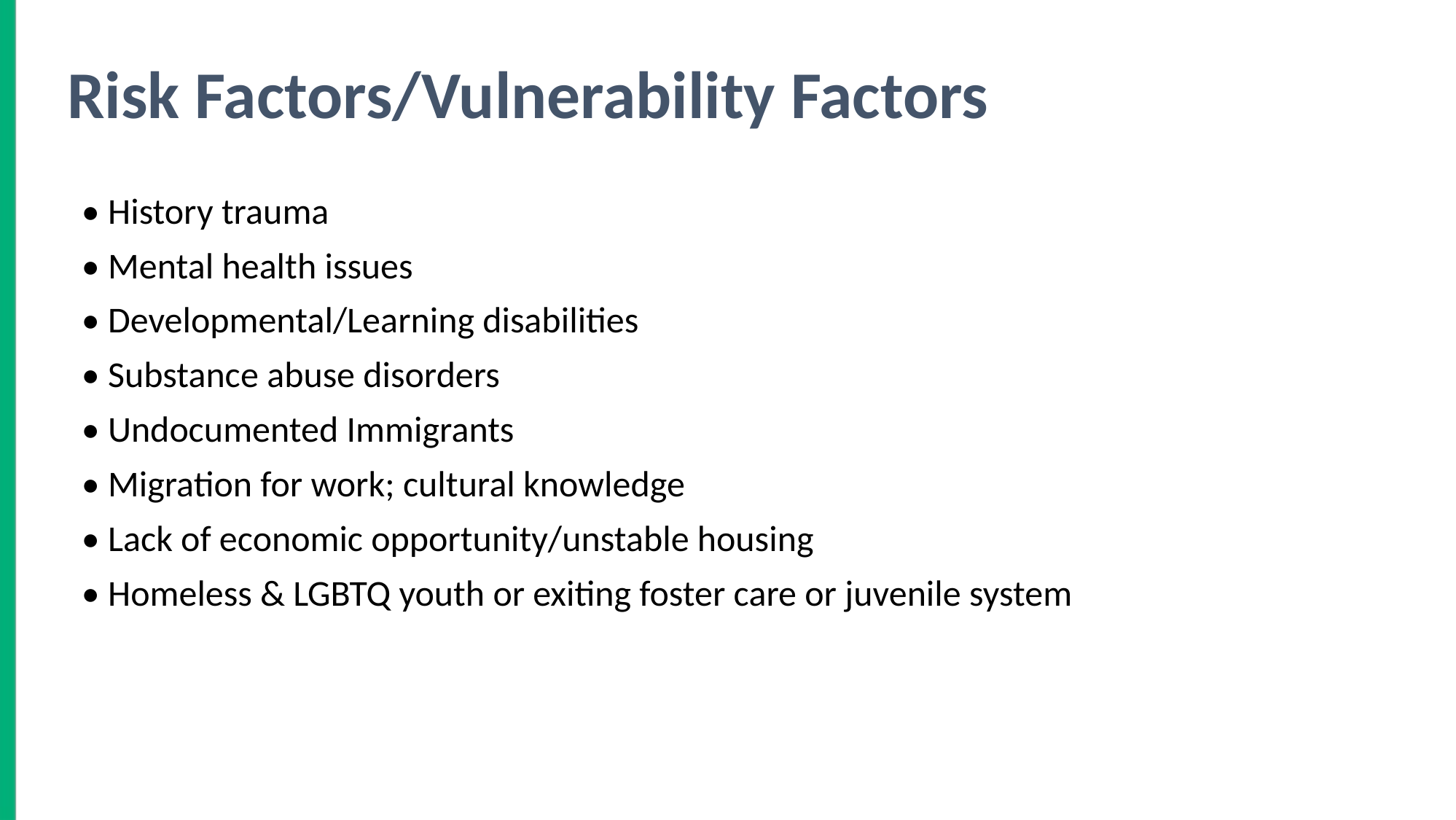

Risk Factors/Vulnerability Factors
• History trauma
• Mental health issues
• Developmental/Learning disabilities
• Substance abuse disorders
• Undocumented Immigrants
• Migration for work; cultural knowledge
• Lack of economic opportunity/unstable housing
• Homeless & LGBTQ youth or exiting foster care or juvenile system

## Slide 17
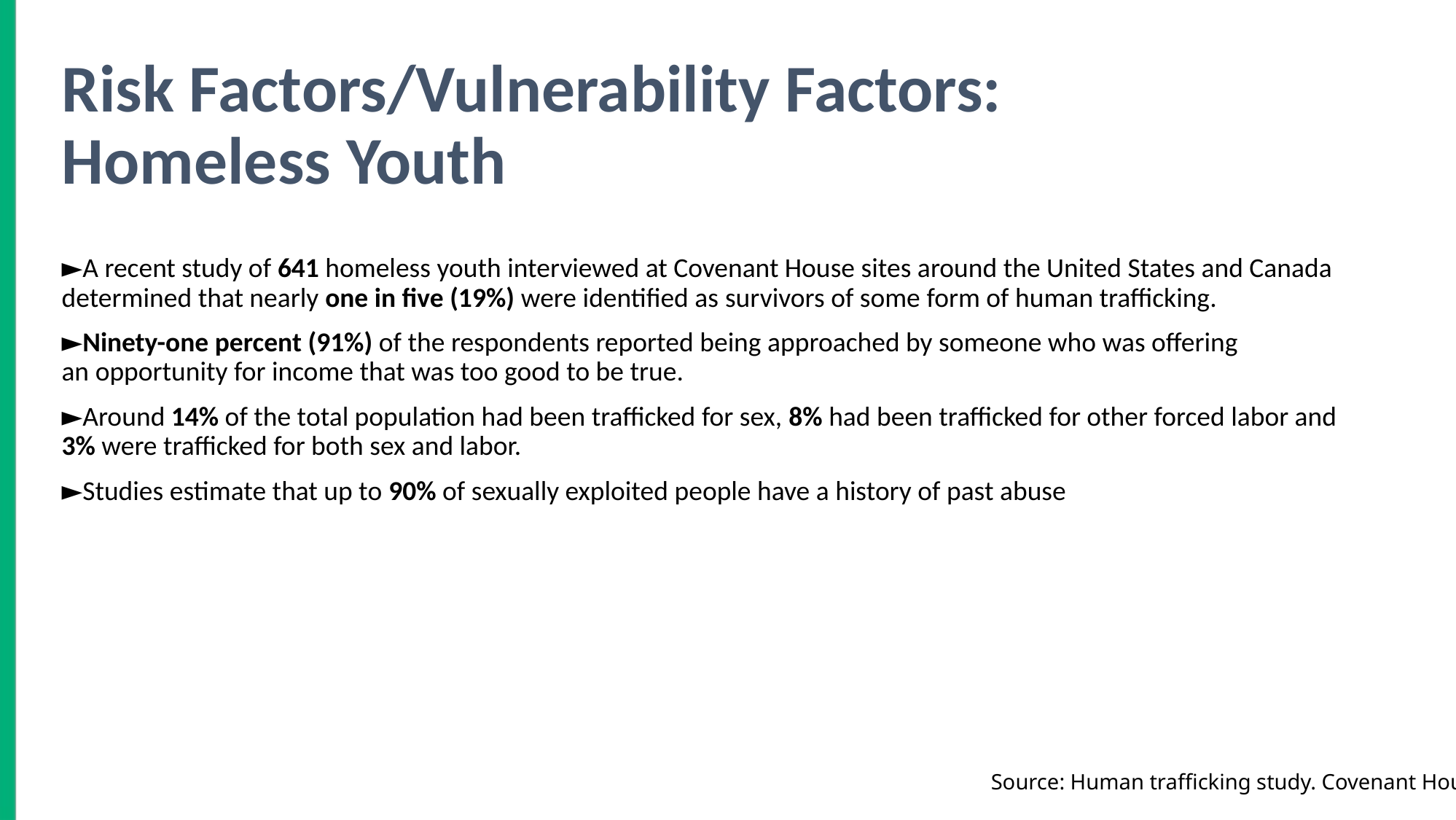

Risk Factors/Vulnerability Factors: Homeless Youth
►A recent study of 641 homeless youth interviewed at Covenant House sites around the United States and Canada determined that nearly one in five (19%) were identified as survivors of some form of human trafficking.
►Ninety-one percent (91%) of the respondents reported being approached by someone who was offering an opportunity for income that was too good to be true.
►Around 14% of the total population had been trafficked for sex, 8% had been trafficked for other forced labor and 3% were trafficked for both sex and labor.
►Studies estimate that up to 90% of sexually exploited people have a history of past abuse
Source: Human trafficking study. Covenant House.

## Slide 18
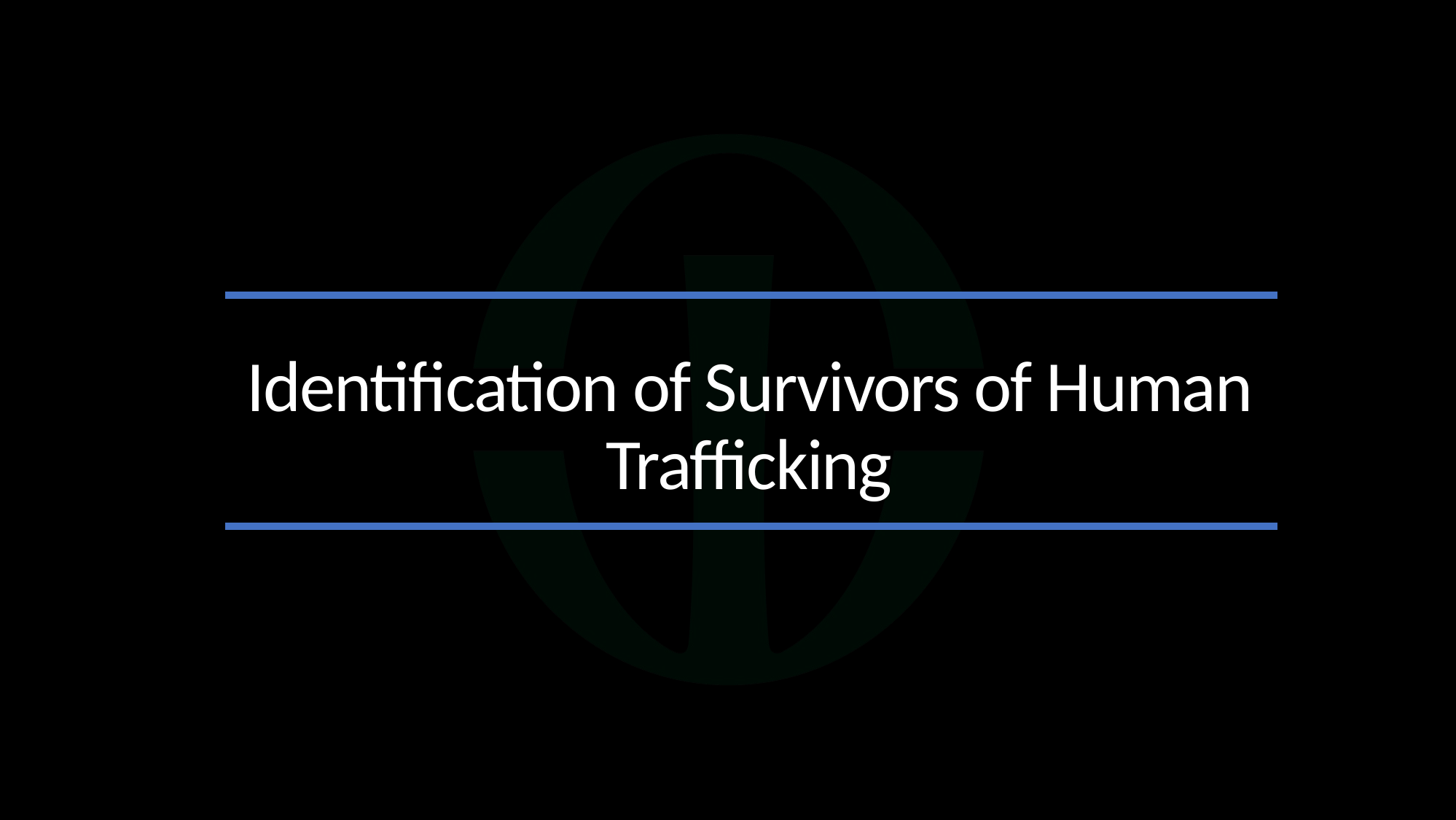

Identification of Survivors of Human Trafficking

## Slide 19
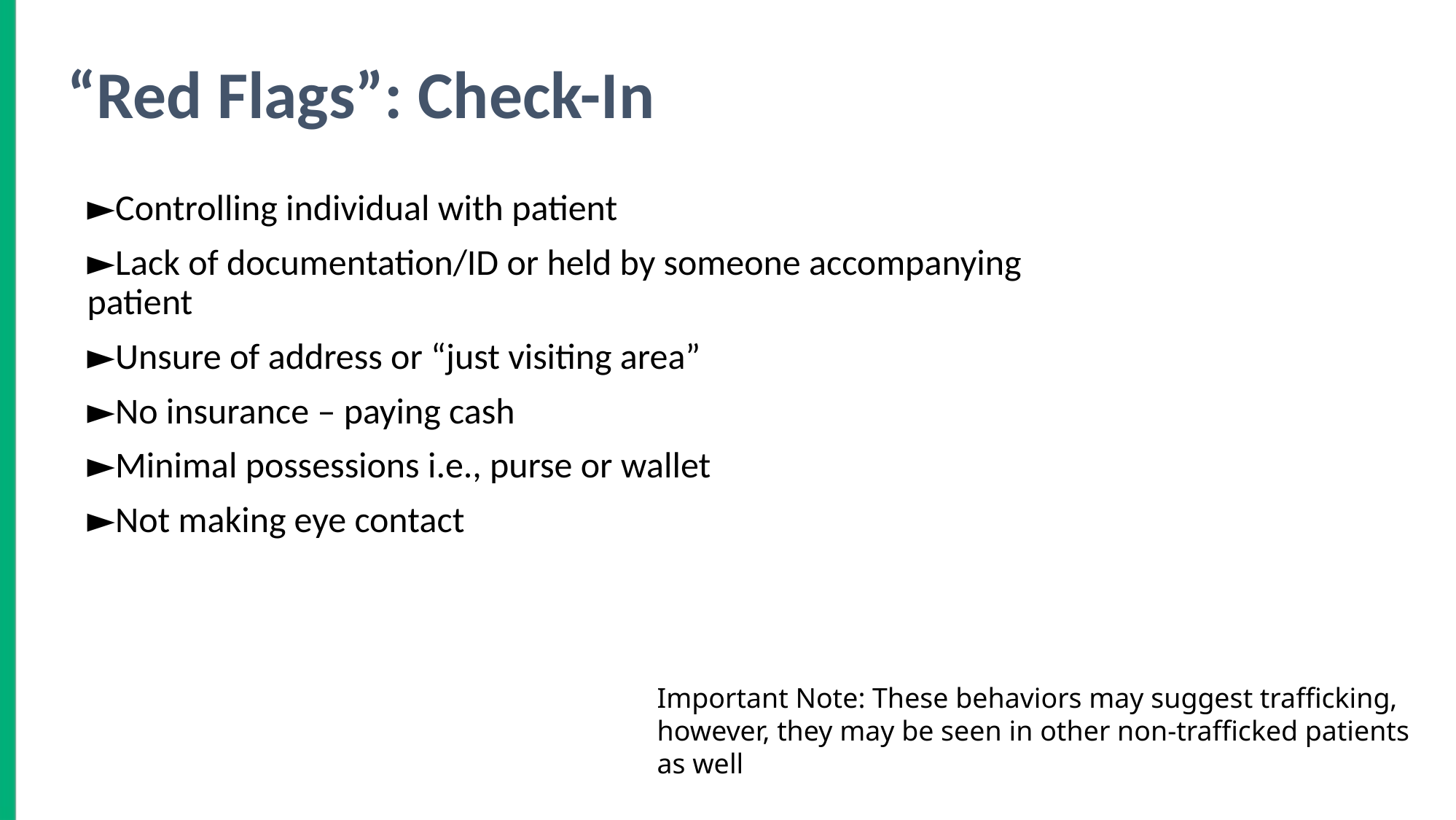

“Red Flags”: Check-In
►Controlling individual with patient
►Lack of documentation/ID or held by someone accompanying patient
►Unsure of address or “just visiting area”
►No insurance – paying cash
►Minimal possessions i.e., purse or wallet
►Not making eye contact
Important Note: These behaviors may suggest trafficking, however, they may be seen in other non-trafficked patients as well

## Slide 20
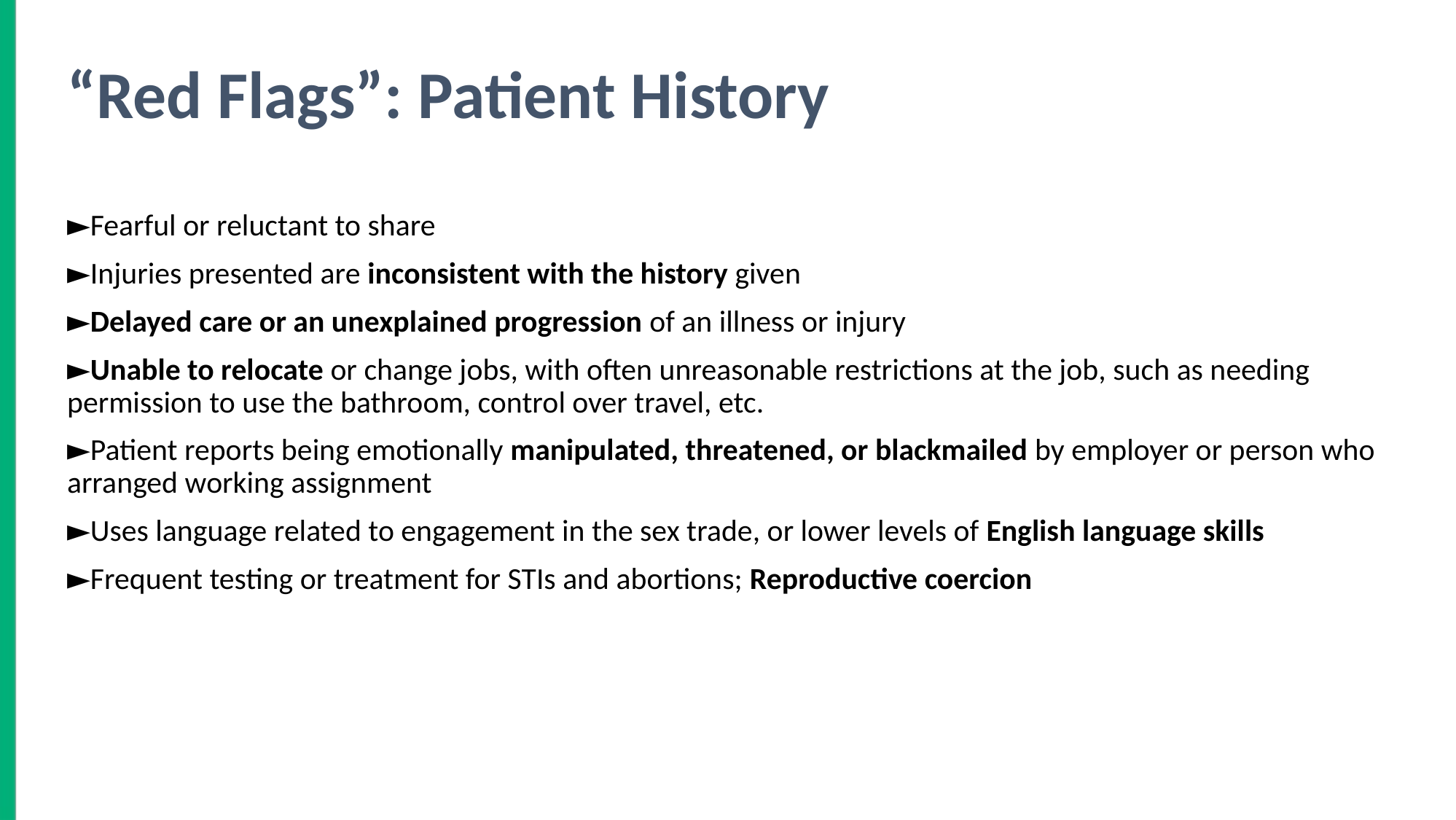

“Red Flags”: Patient History
►Fearful or reluctant to share
►Injuries presented are inconsistent with the history given
►Delayed care or an unexplained progression of an illness or injury
►Unable to relocate or change jobs, with often unreasonable restrictions at the job, such as needing permission to use the bathroom, control over travel, etc.
►Patient reports being emotionally manipulated, threatened, or blackmailed by employer or person who arranged working assignment
►Uses language related to engagement in the sex trade, or lower levels of English language skills
►Frequent testing or treatment for STIs and abortions; Reproductive coercion

## Slide 21
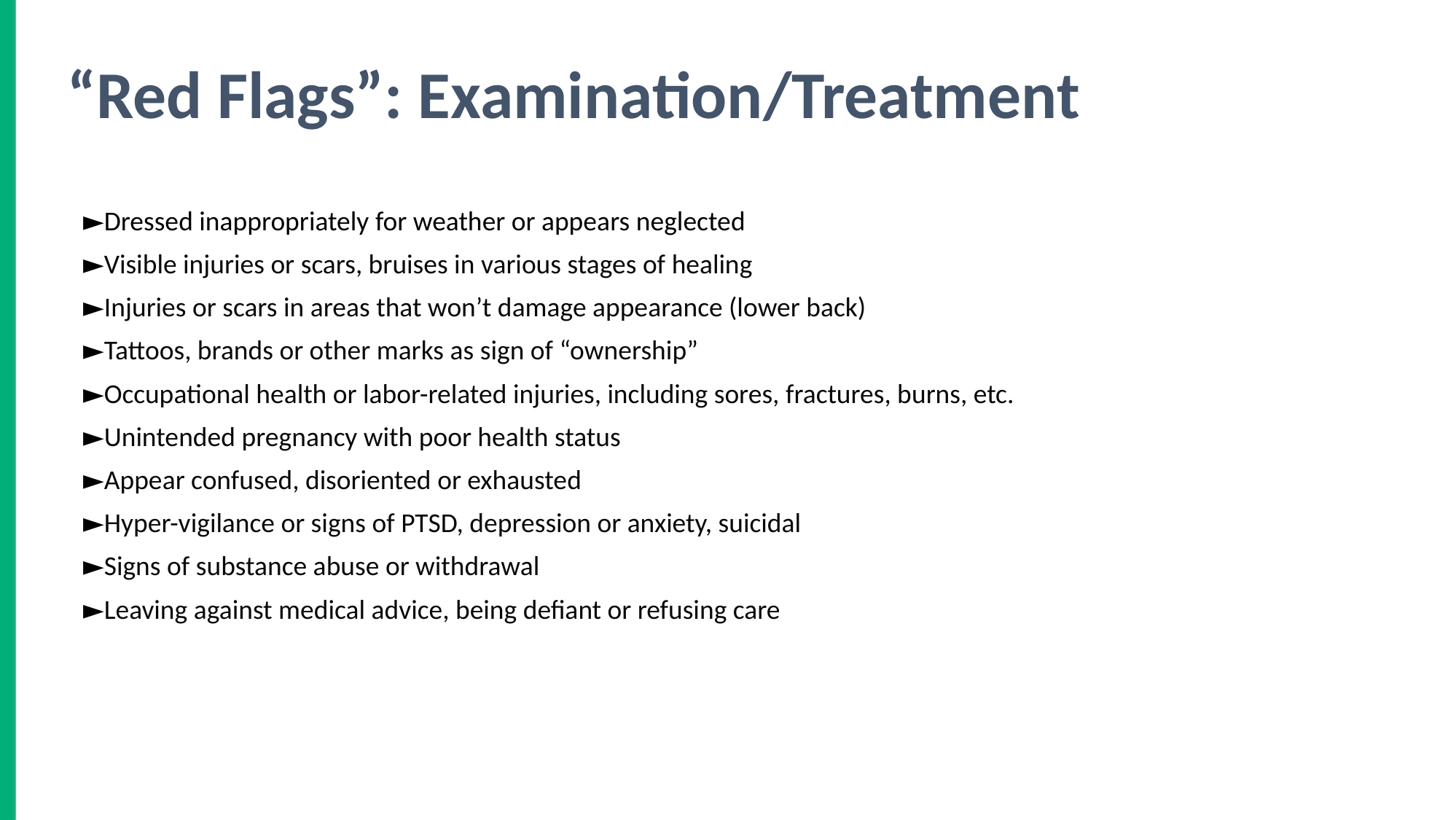

“Red Flags”: Examination/Treatment
►Dressed inappropriately for weather or appears neglected
►Visible injuries or scars, bruises in various stages of healing
►Injuries or scars in areas that won’t damage appearance (lower back)
►Tattoos, brands or other marks as sign of “ownership”
►Occupational health or labor-related injuries, including sores, fractures, burns, etc.
►Unintended pregnancy with poor health status
►Appear confused, disoriented or exhausted
►Hyper-vigilance or signs of PTSD, depression or anxiety, suicidal
►Signs of substance abuse or withdrawal
►Leaving against medical advice, being defiant or refusing care

## Slide 22
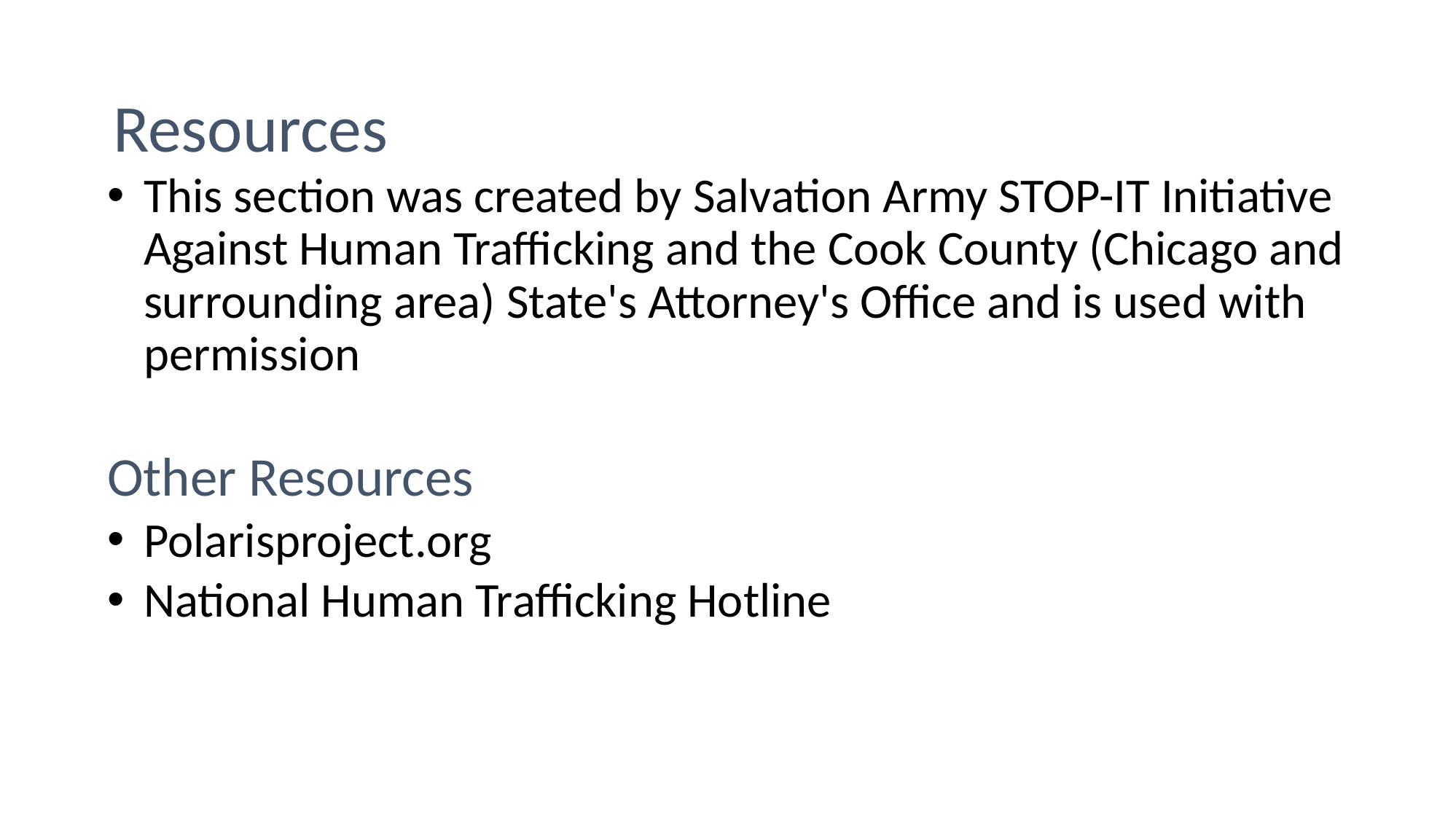

# Resources
This section was created by Salvation Army STOP-IT Initiative Against Human Trafficking and the Cook County (Chicago and surrounding area) State's Attorney's Office and is used with permission
Other Resources
Polarisproject.org
National Human Trafficking Hotline

## Slide 23
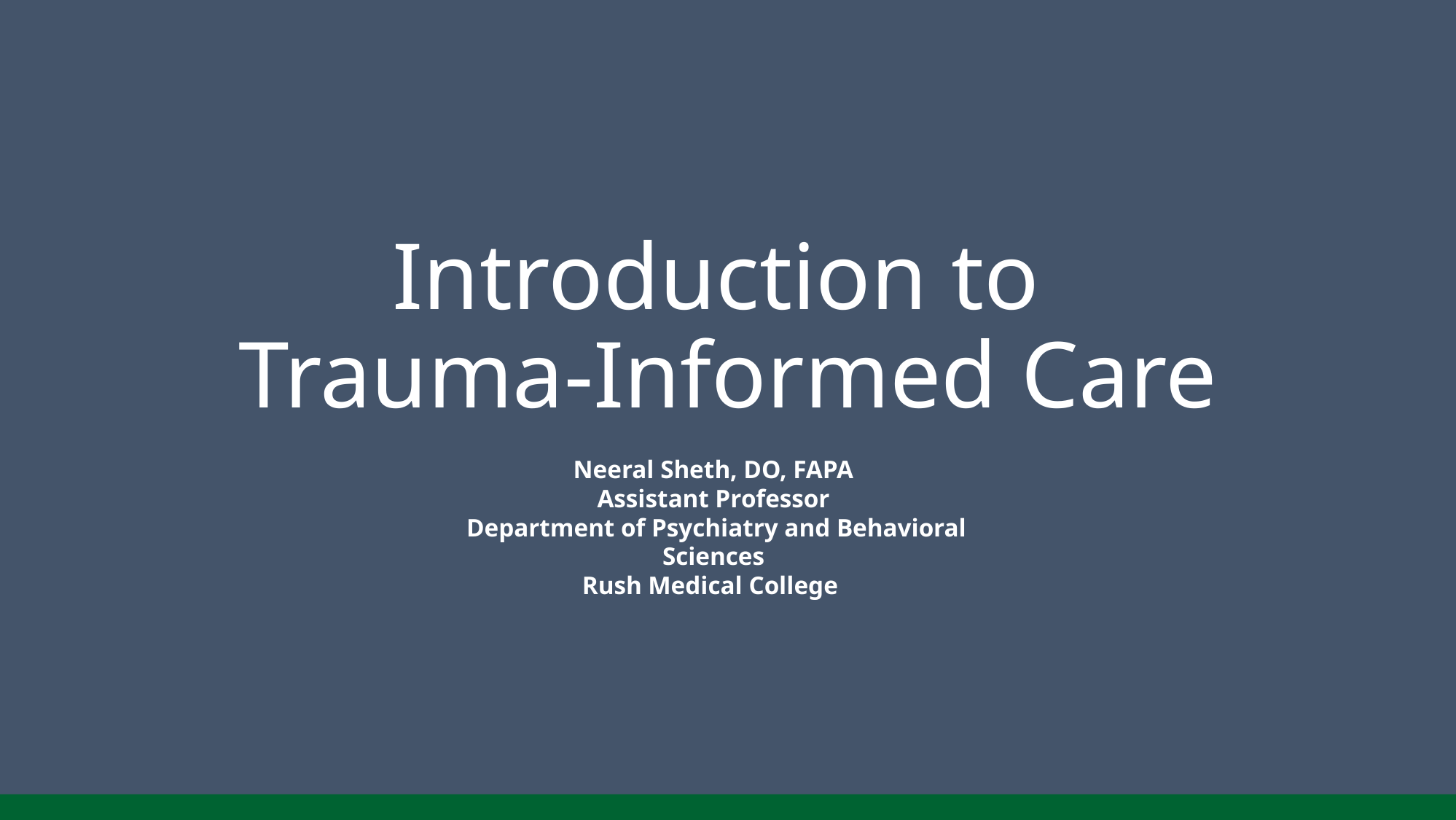

# Introduction to  Trauma-Informed Care
Neeral Sheth, DO, FAPA
Assistant Professor
Department of Psychiatry and Behavioral Sciences
Rush Medical College

## Slide 24
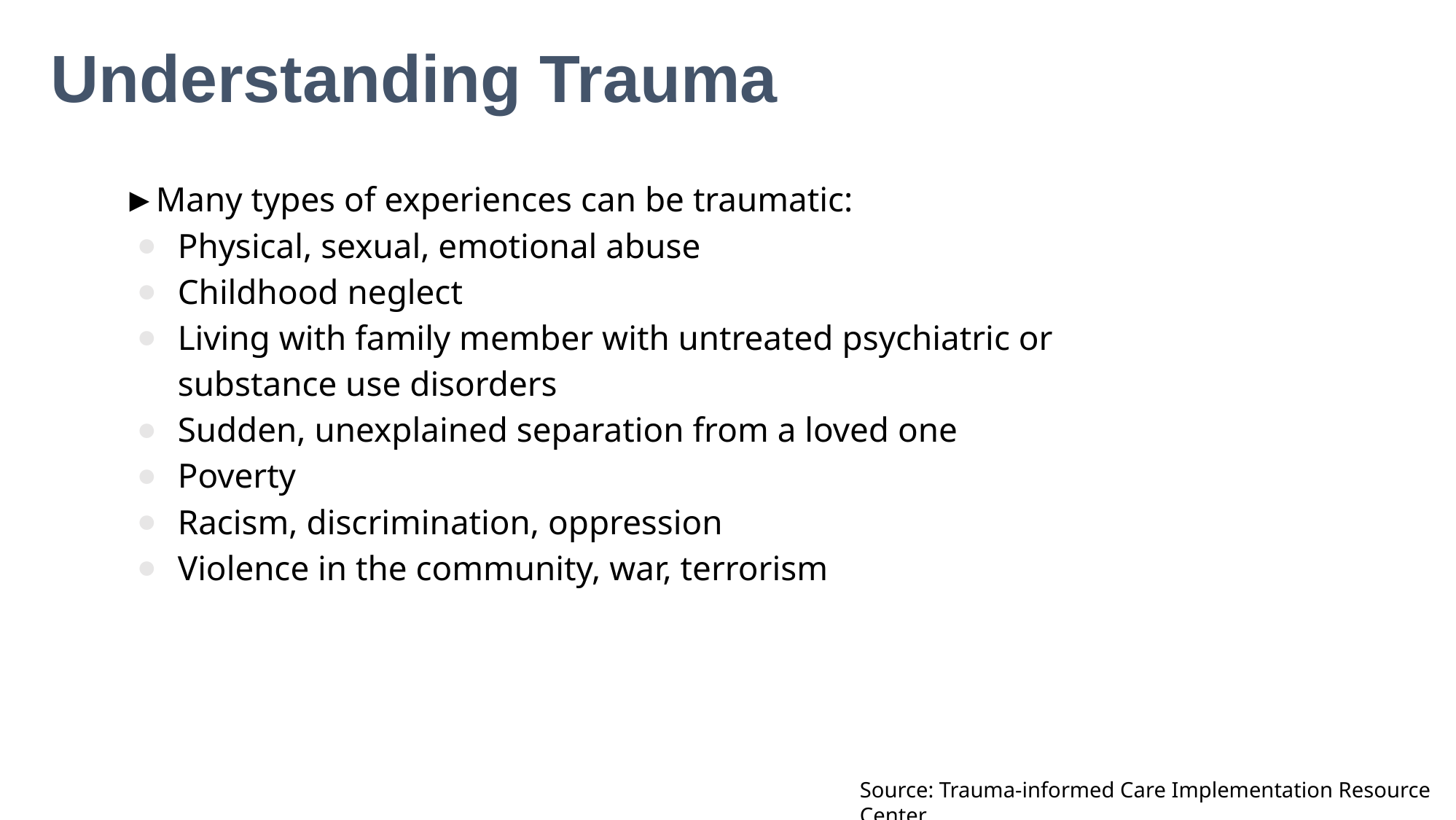

Understanding Trauma
►Many types of experiences can be traumatic:
Physical, sexual, emotional abuse
Childhood neglect
Living with family member with untreated psychiatric or substance use disorders
Sudden, unexplained separation from a loved one
Poverty
Racism, discrimination, oppression
Violence in the community, war, terrorism
Source: Trauma-informed Care Implementation Resource Center

## Slide 25
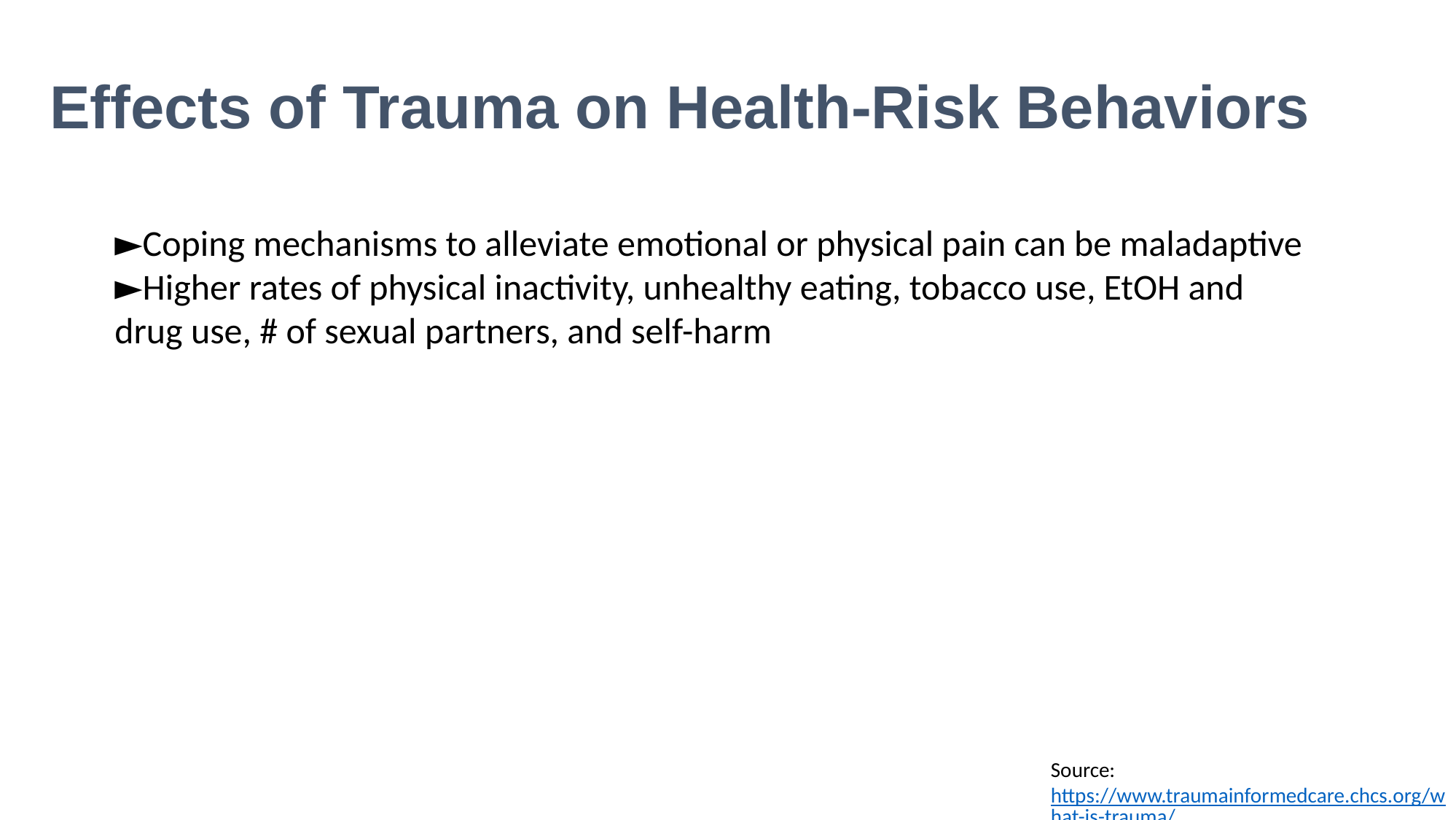

Effects of Trauma on Health-Risk Behaviors
​
►Coping mechanisms to alleviate emotional or physical pain can be maladaptive​
►Higher rates of physical inactivity, unhealthy eating, tobacco use, EtOH and drug use, # of sexual partners, and self-harm
Source: https://www.traumainformedcare.chcs.org/what-is-trauma/

## Slide 26
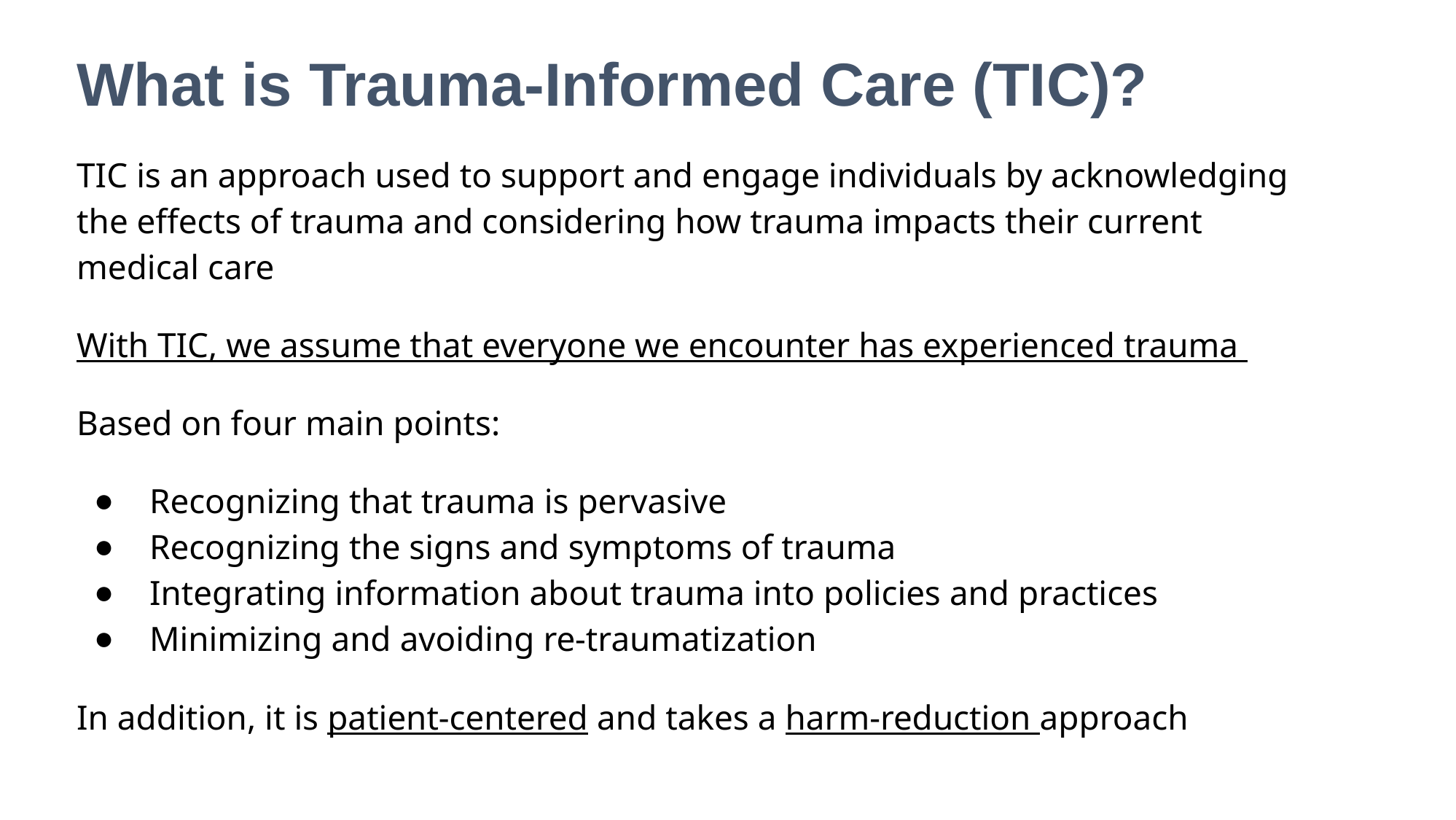

What is Trauma-Informed Care (TIC)?
TIC is an approach used to support and engage individuals by acknowledging the effects of trauma and considering how trauma impacts their current medical care
With TIC, we assume that everyone we encounter has experienced trauma
Based on four main points:
Recognizing that trauma is pervasive
Recognizing the signs and symptoms of trauma
Integrating information about trauma into policies and practices
Minimizing and avoiding re-traumatization
In addition, it is patient-centered and takes a harm-reduction approach

## Slide 27
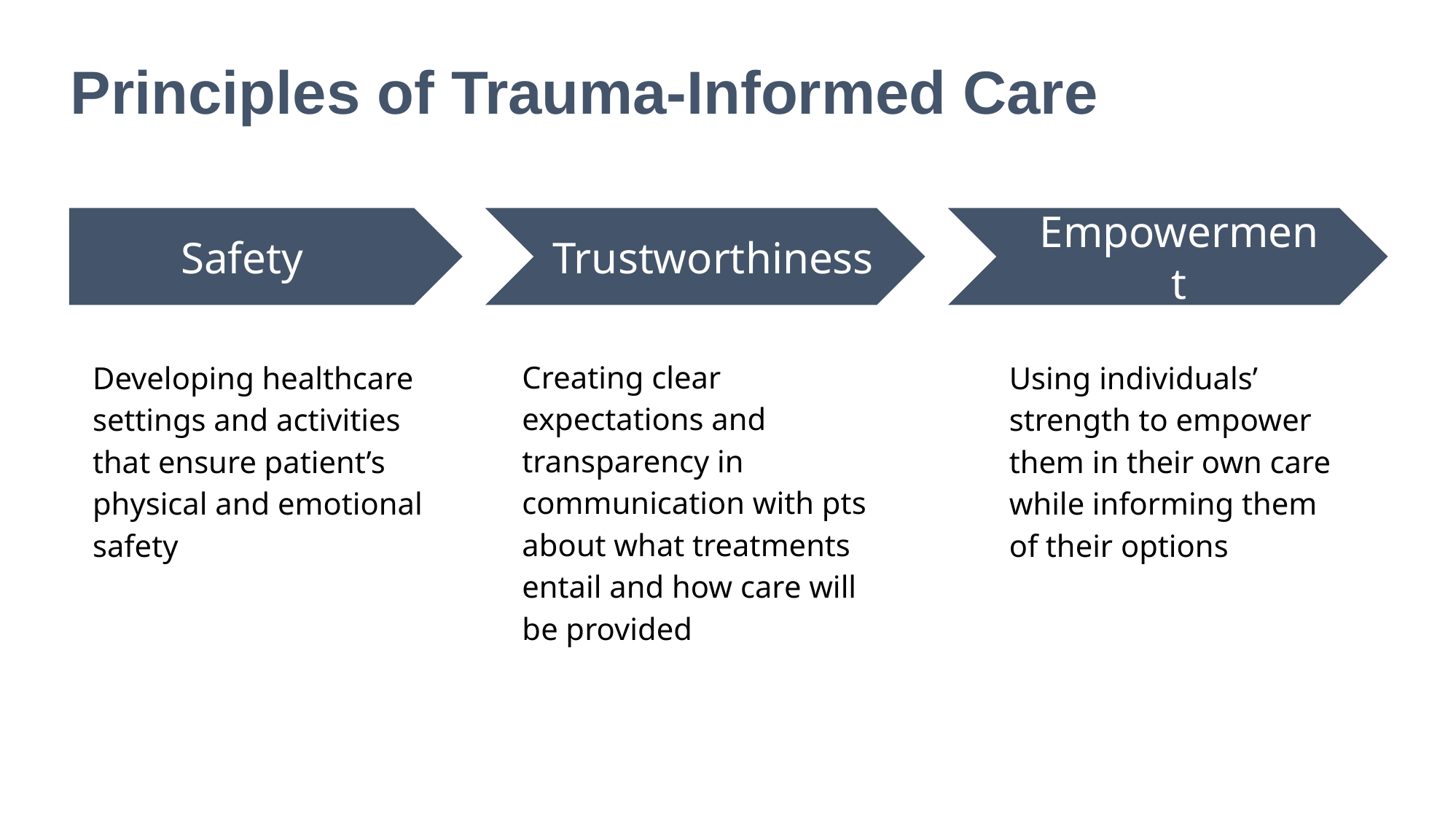

Principles of Trauma-Informed Care
Trustworthiness
Empowerment
Safety
Creating clear expectations and transparency in communication with pts about what treatments entail and how care will be provided
Developing healthcare settings and activities that ensure patient’s physical and emotional safety
Using individuals’ strength to empower them in their own care while informing them of their options

## Slide 28
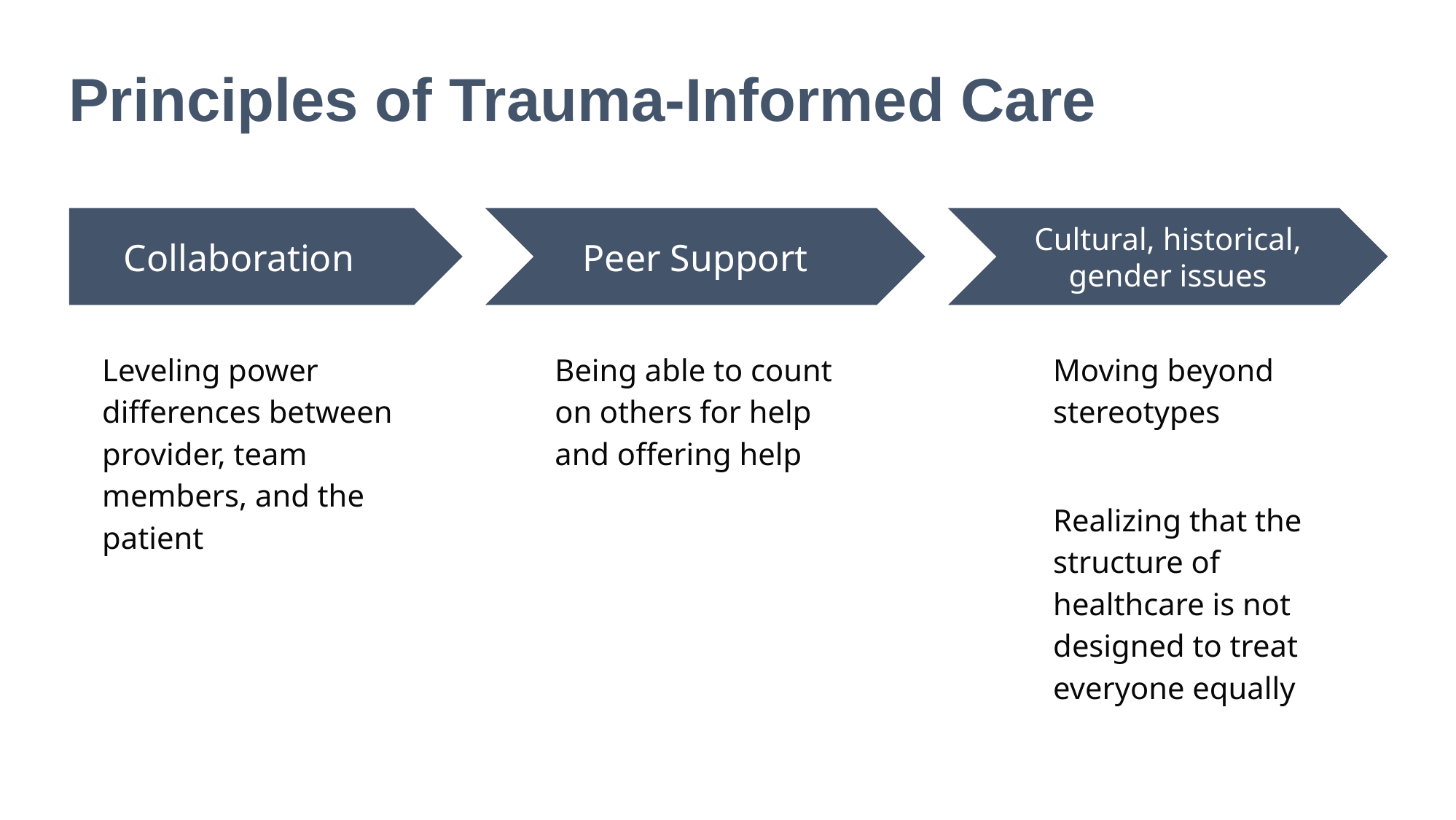

Principles of Trauma-Informed Care
Cultural, historical, gender issues
Collaboration
Peer Support
Leveling power differences between provider, team members, and the patient
Being able to count on others for help and offering help
Moving beyond stereotypes
Realizing that the structure of healthcare is not designed to treat everyone equally

## Slide 29
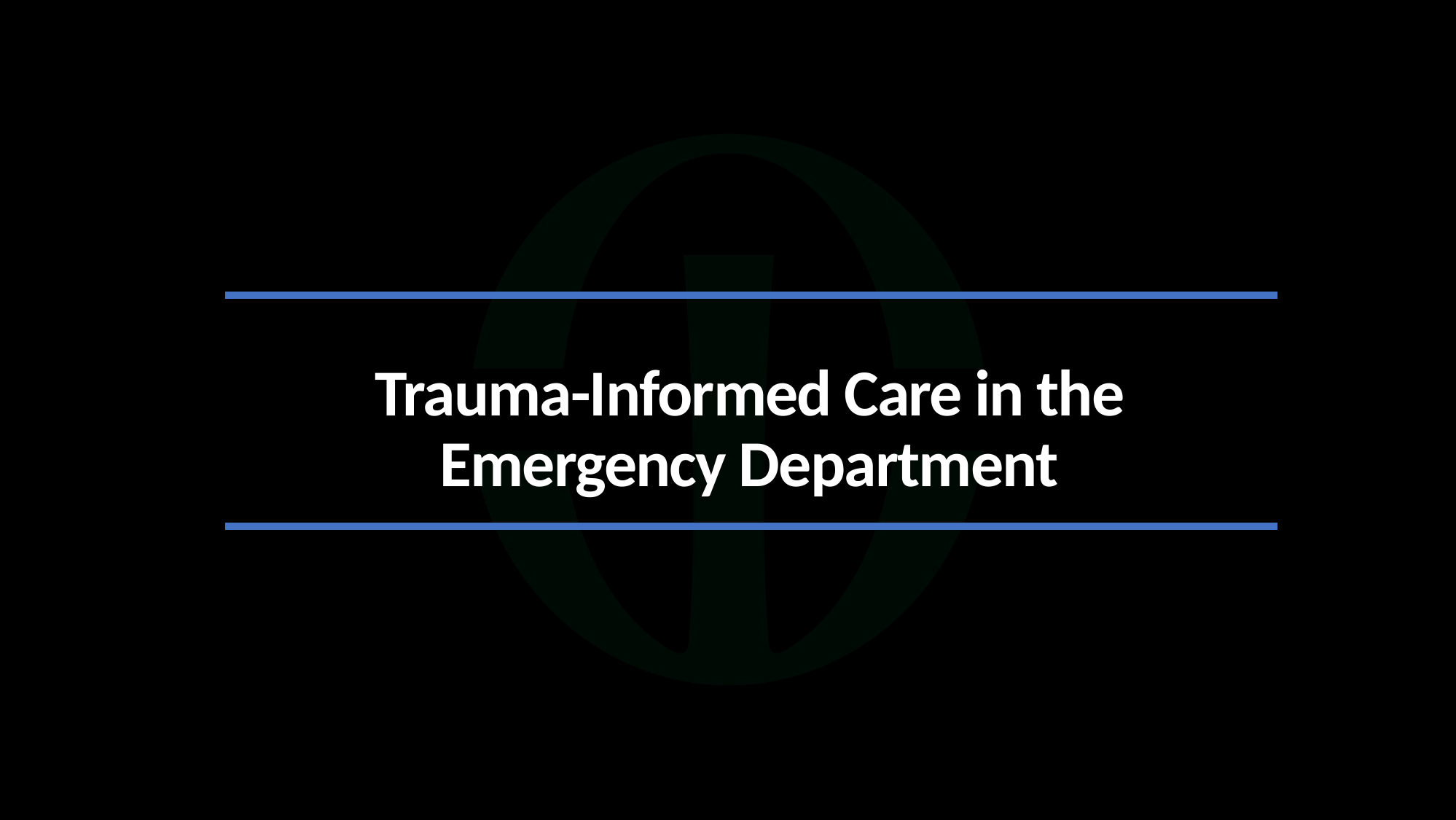

Trauma-Informed Care in the Emergency Department

## Slide 30
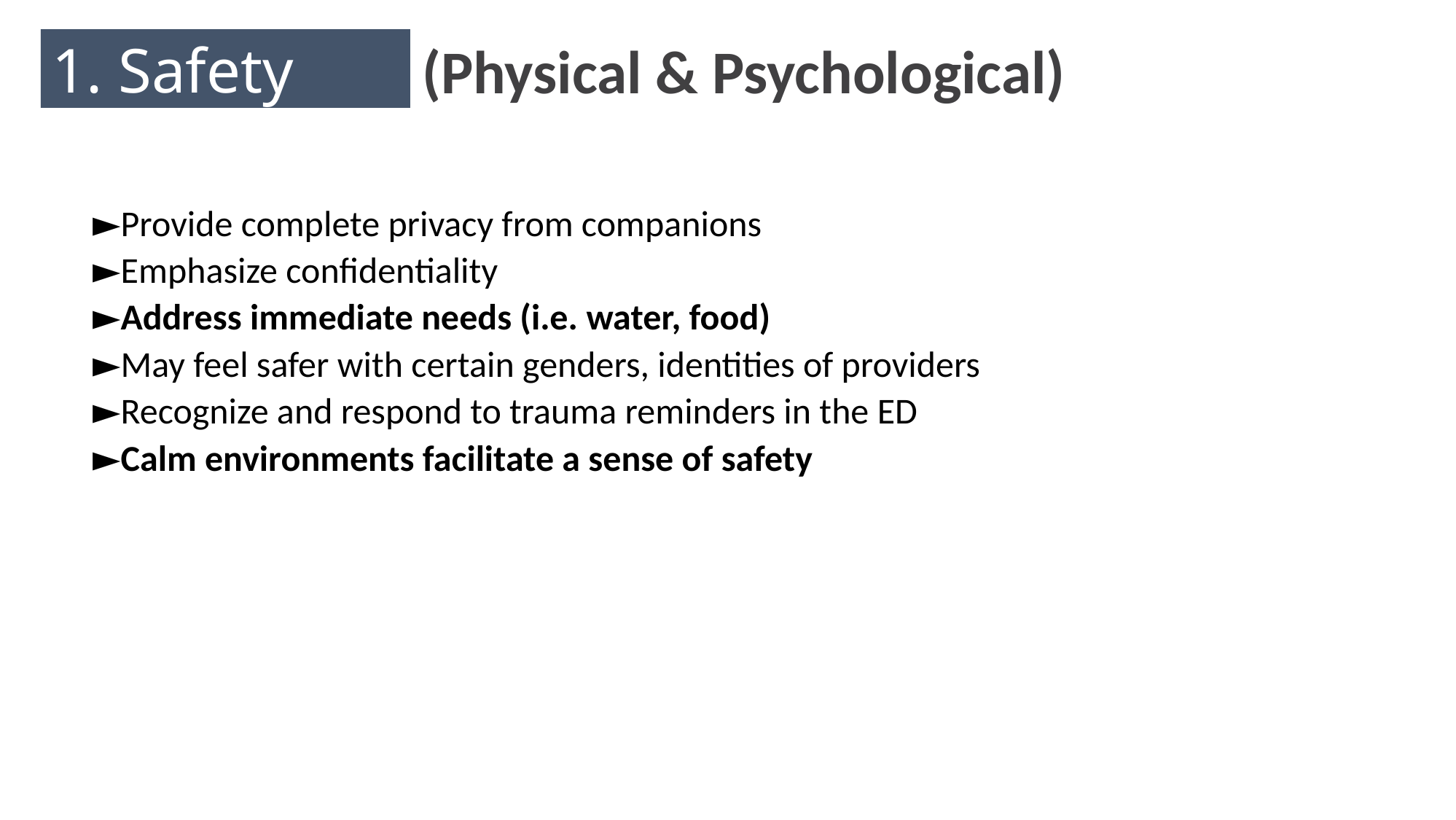

1. Safety
(Physical & Psychological)
►Provide complete privacy from companions
►Emphasize confidentiality
►Address immediate needs (i.e. water, food)
►May feel safer with certain genders, identities of providers
►Recognize and respond to trauma reminders in the ED
►Calm environments facilitate a sense of safety

## Slide 31
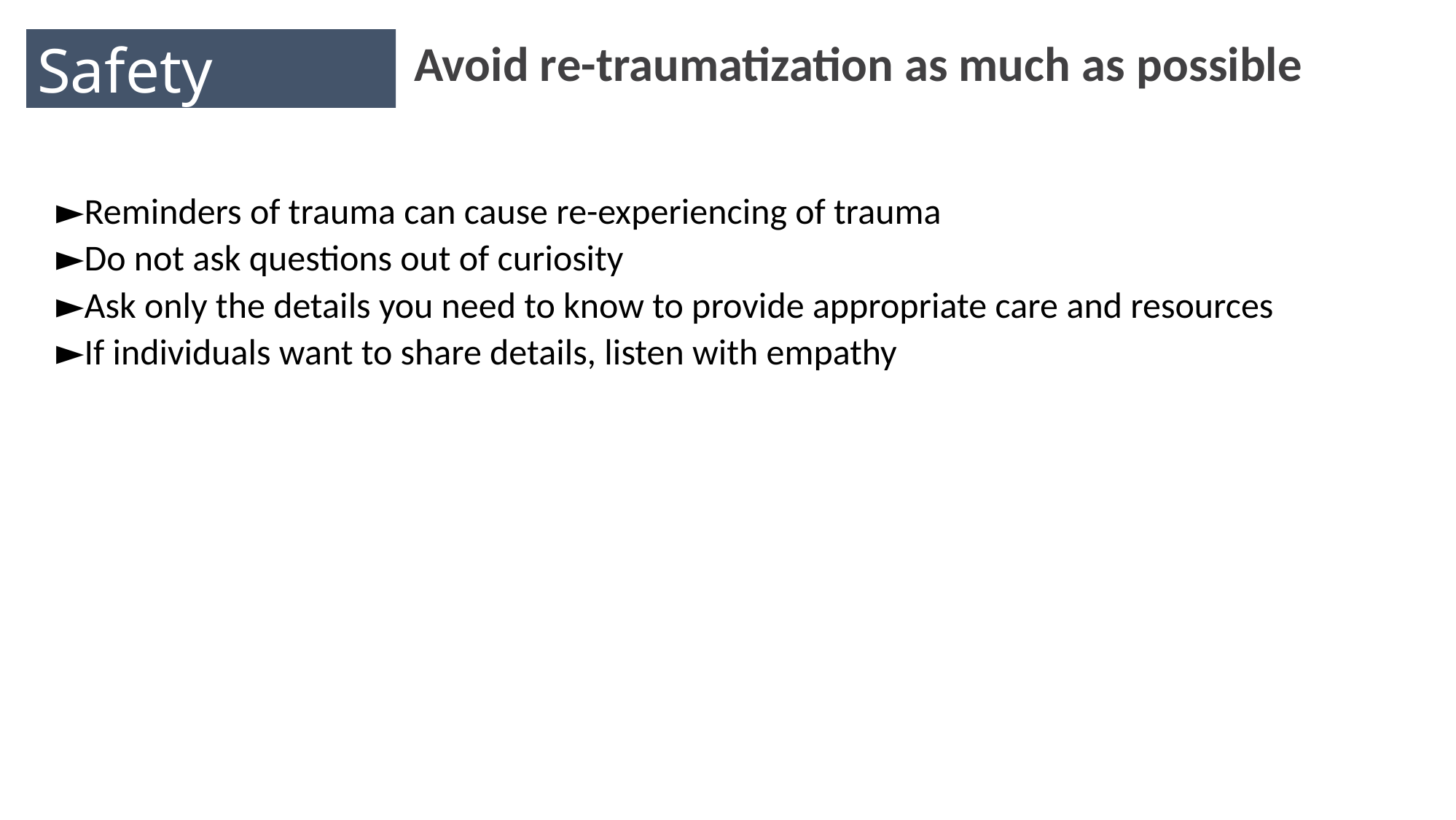

Safety
Avoid re-traumatization as much as possible
►Reminders of trauma can cause re-experiencing of trauma
►Do not ask questions out of curiosity
►Ask only the details you need to know to provide appropriate care and resources
►If individuals want to share details, listen with empathy

## Slide 32
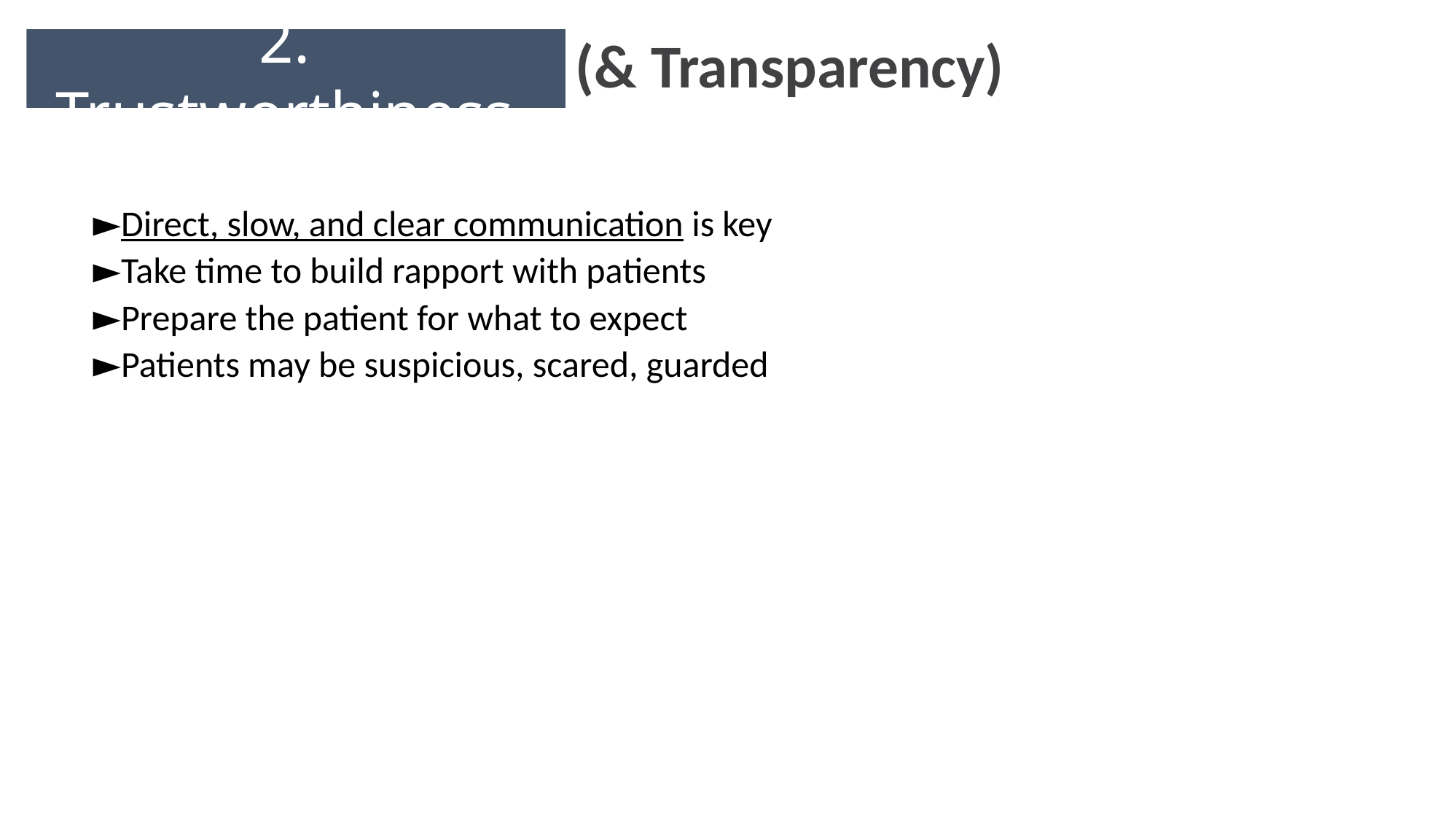

2. Trustworthiness
(& Transparency)
►Direct, slow, and clear communication is key
►Take time to build rapport with patients
►Prepare the patient for what to expect
►Patients may be suspicious, scared, guarded

## Slide 33
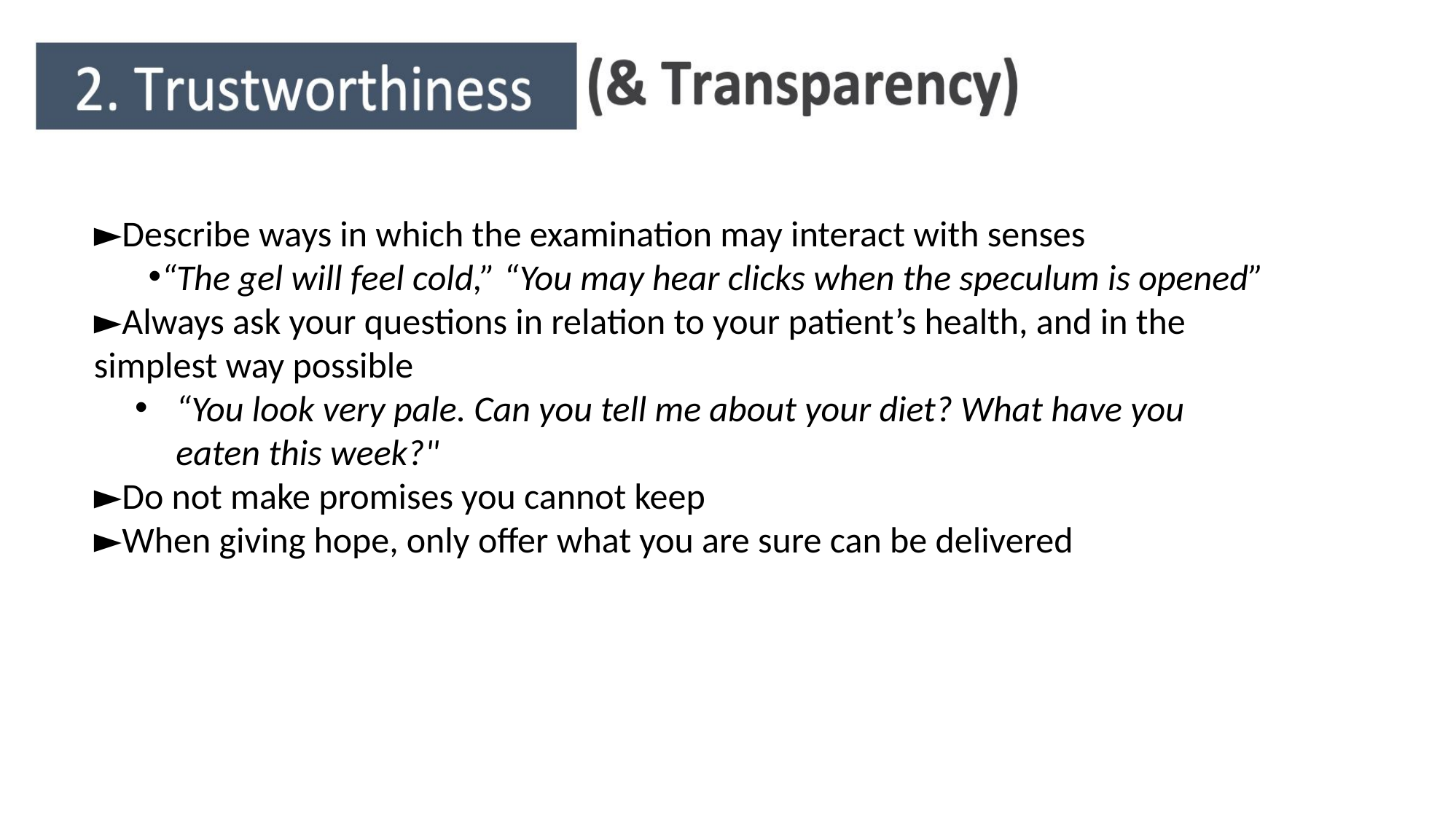

►Describe ways in which the examination may interact with senses​
“The gel will feel cold,” “You may hear clicks when the speculum is opened”
►Always ask your questions in relation to your patient’s health, and in the simplest way possible​
“You look very pale. Can you tell me about your diet? What have you eaten this week?"
​►Do not make promises you cannot keep​
►When giving hope, only offer what you are sure can be delivered​

## Slide 34
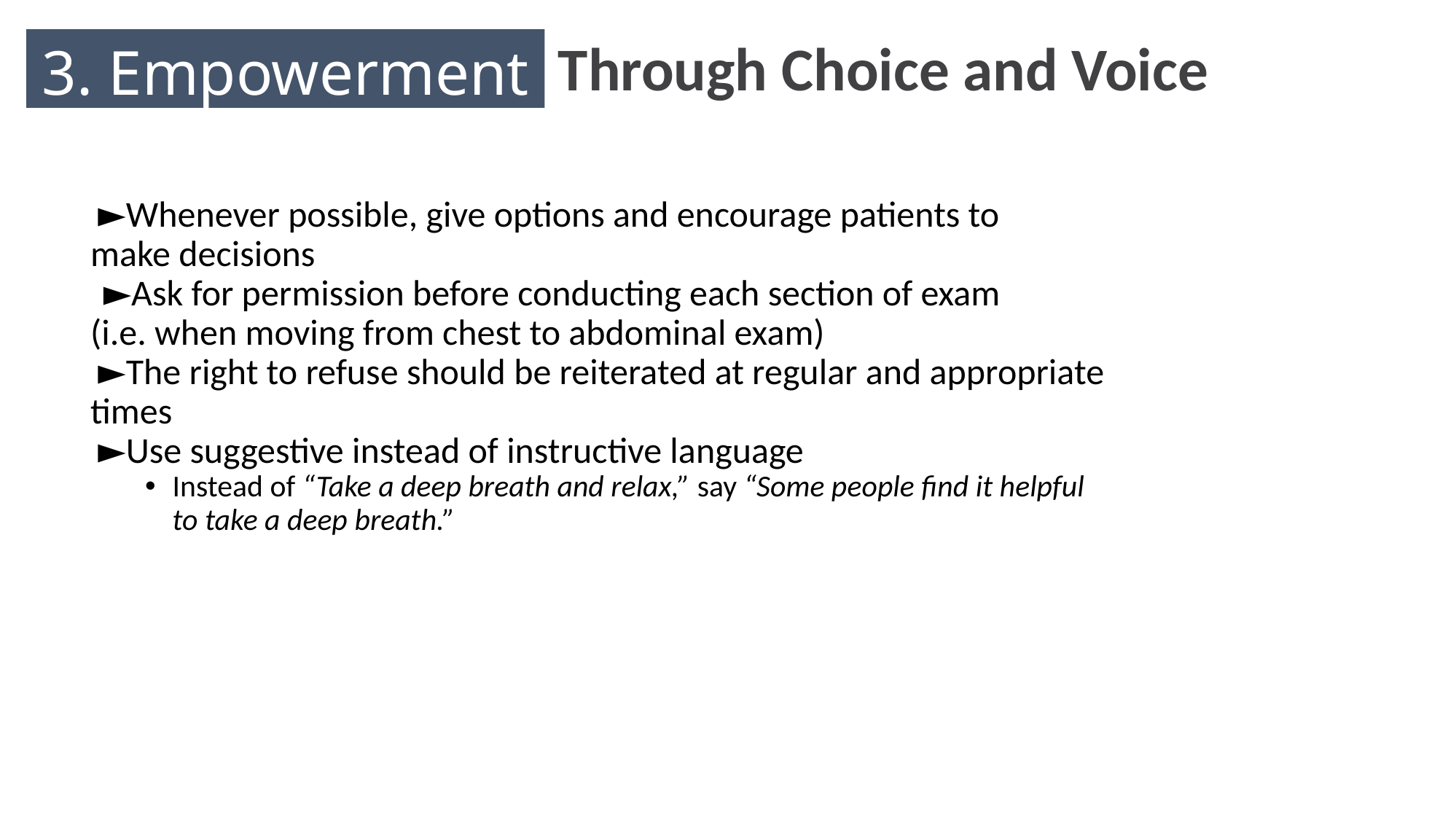

3. Empowerment
 Through Choice and Voice
►Whenever possible, give options and encourage patients to make decisions
►Ask for permission before conducting each section of exam (i.e. when moving from chest to abdominal exam)
►The right to refuse should be reiterated at regular and appropriate times
►Use suggestive instead of instructive language
Instead of “Take a deep breath and relax,” say “Some people find it helpful to take a deep breath.”

## Slide 35
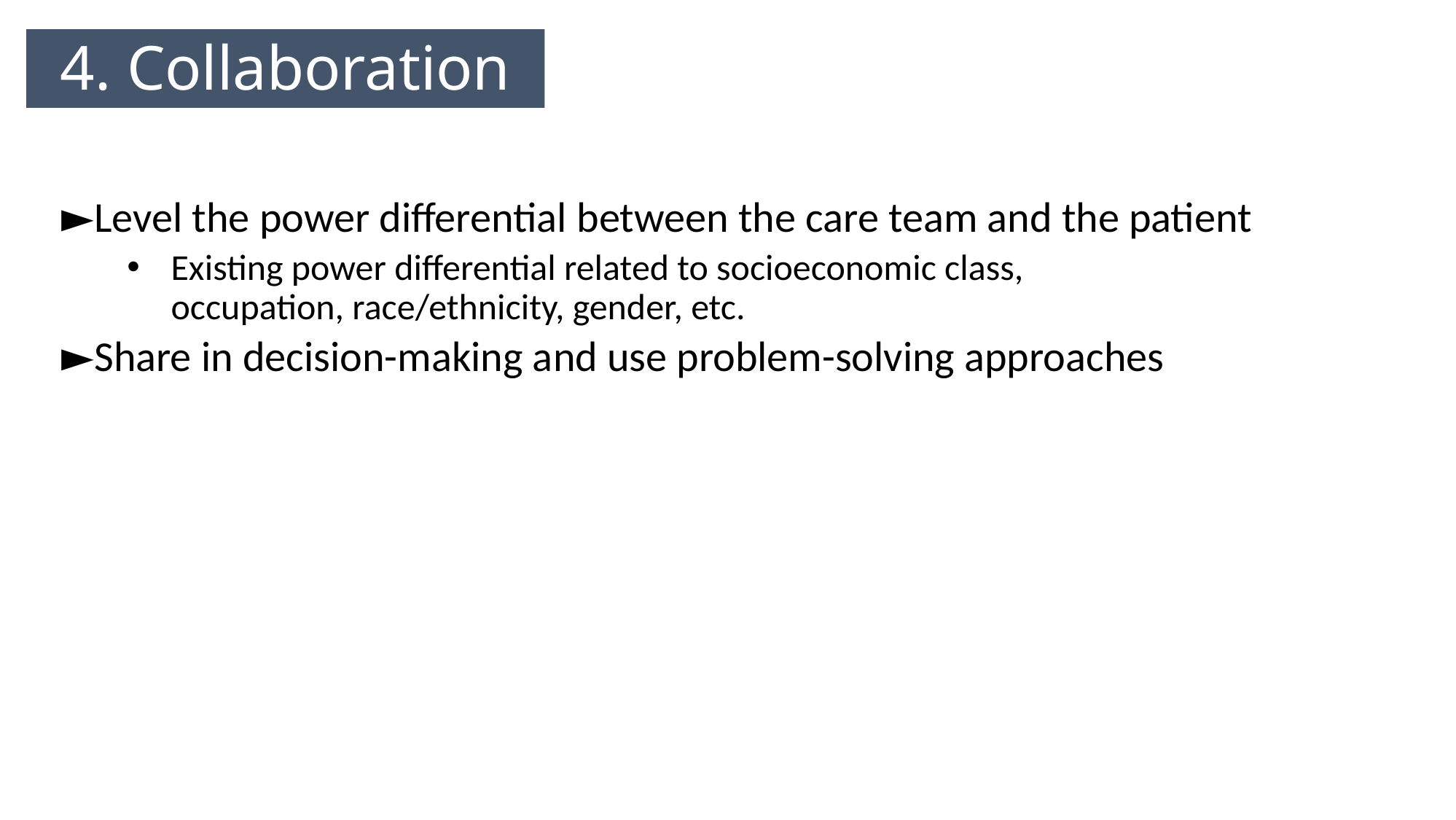

4. Collaboration
►Level the power differential between the care team and the patient
Existing power differential related to socioeconomic class, occupation, race/ethnicity, gender, etc.
►Share in decision-making and use problem-solving approaches

## Slide 36
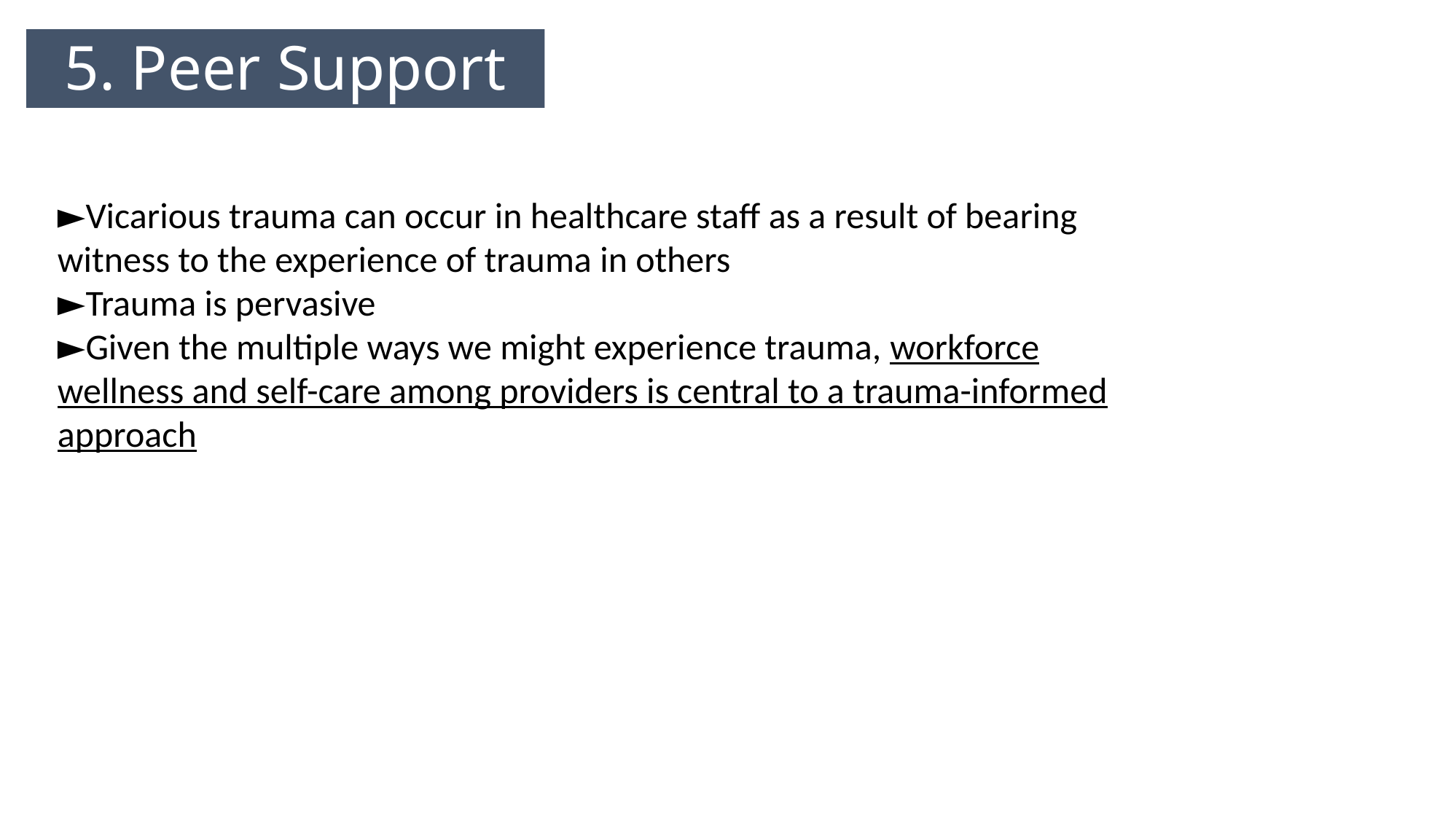

5. Peer Support
►Vicarious trauma can occur in healthcare staff as a result of bearing witness to the experience of trauma in others​
►Trauma is pervasive
►Given the multiple ways we might experience trauma, workforce wellness and self-care among providers is central to a trauma-informed approach​

## Slide 37
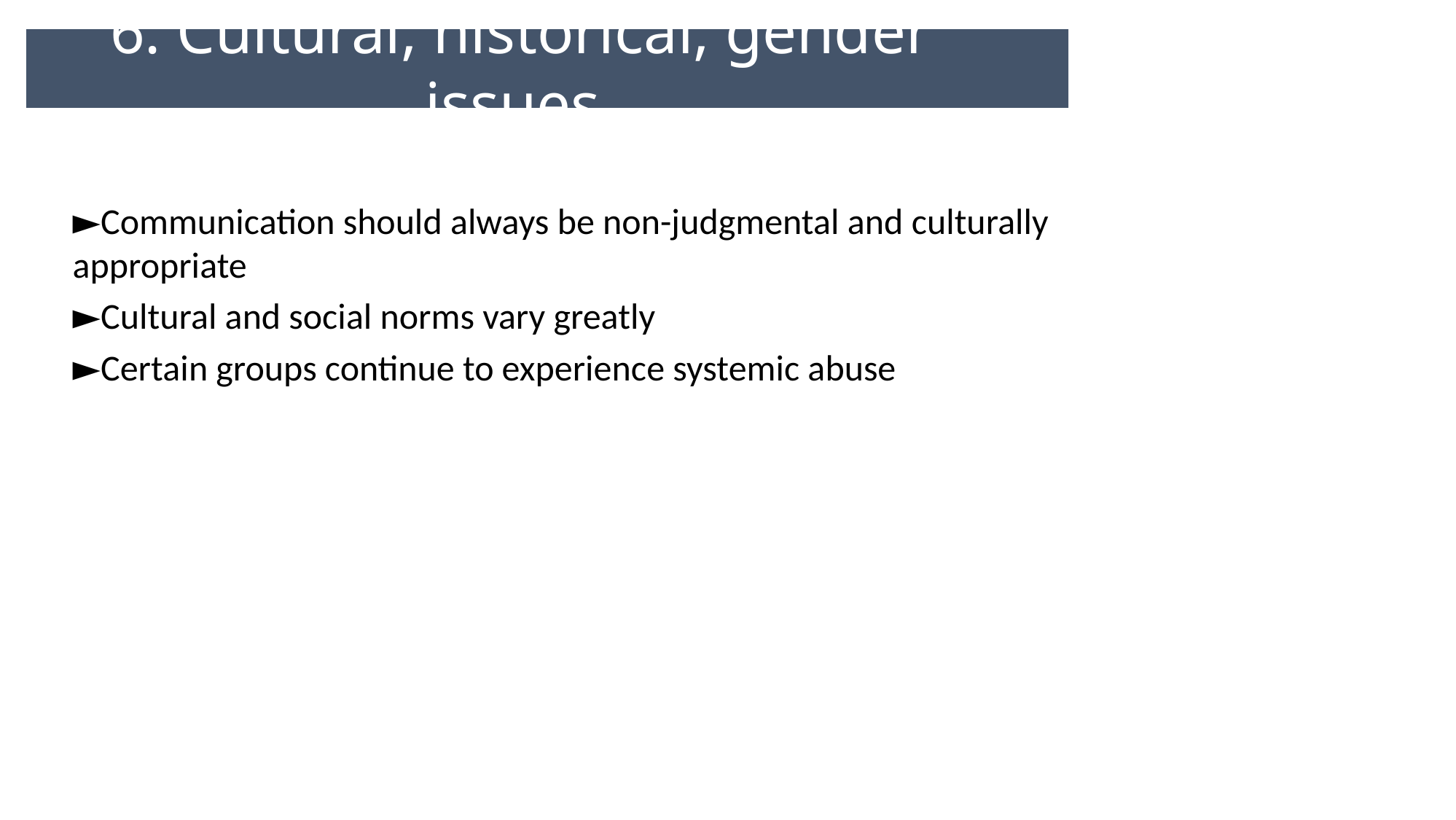

6. Cultural, historical, gender issues
►Communication should always be non-judgmental and culturally appropriate
►Cultural and social norms vary greatly
►Certain groups continue to experience systemic abuse

## Slide 38
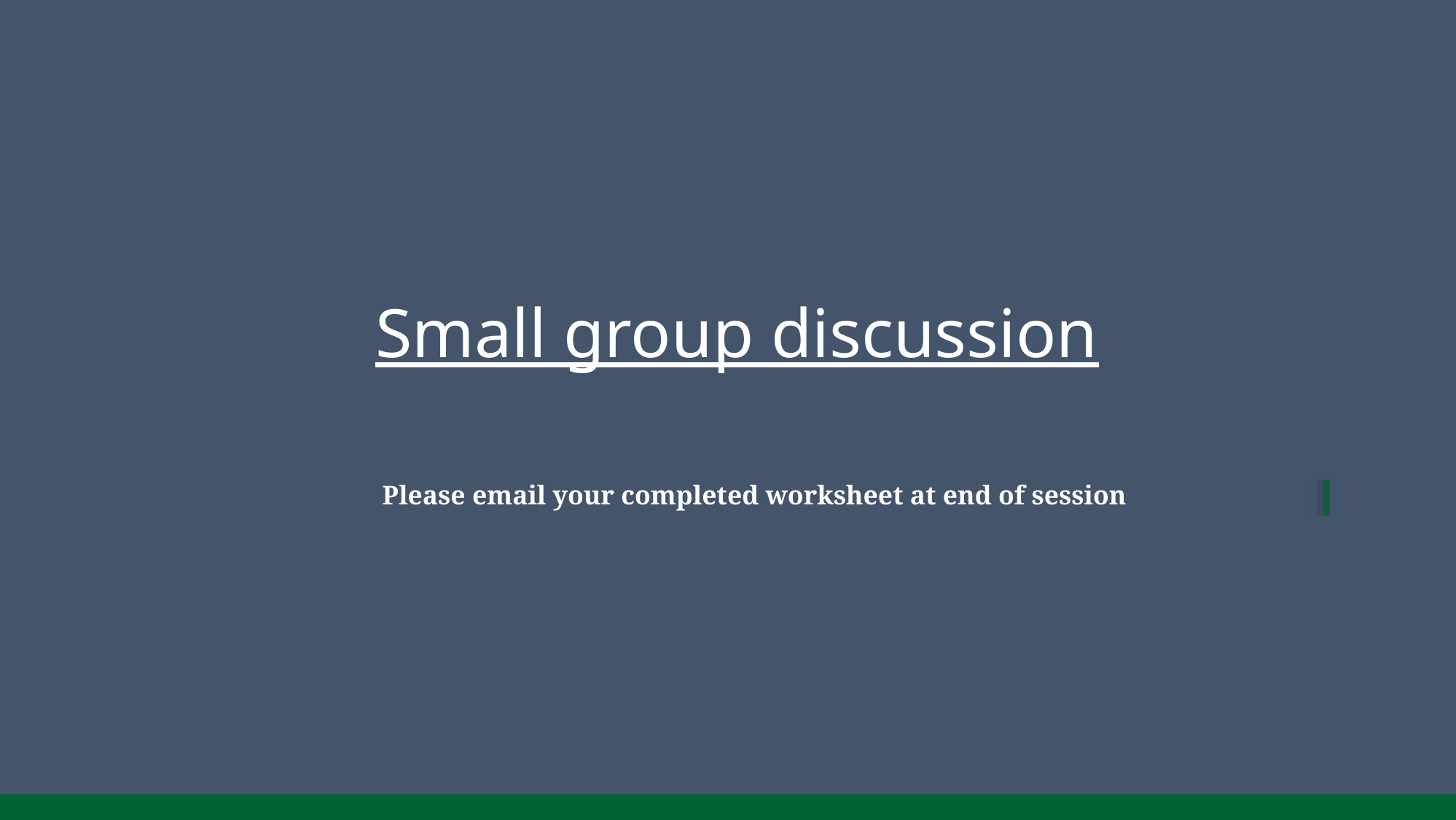

# Small group discussion
Please email your completed worksheet at end of session

## Slide 39
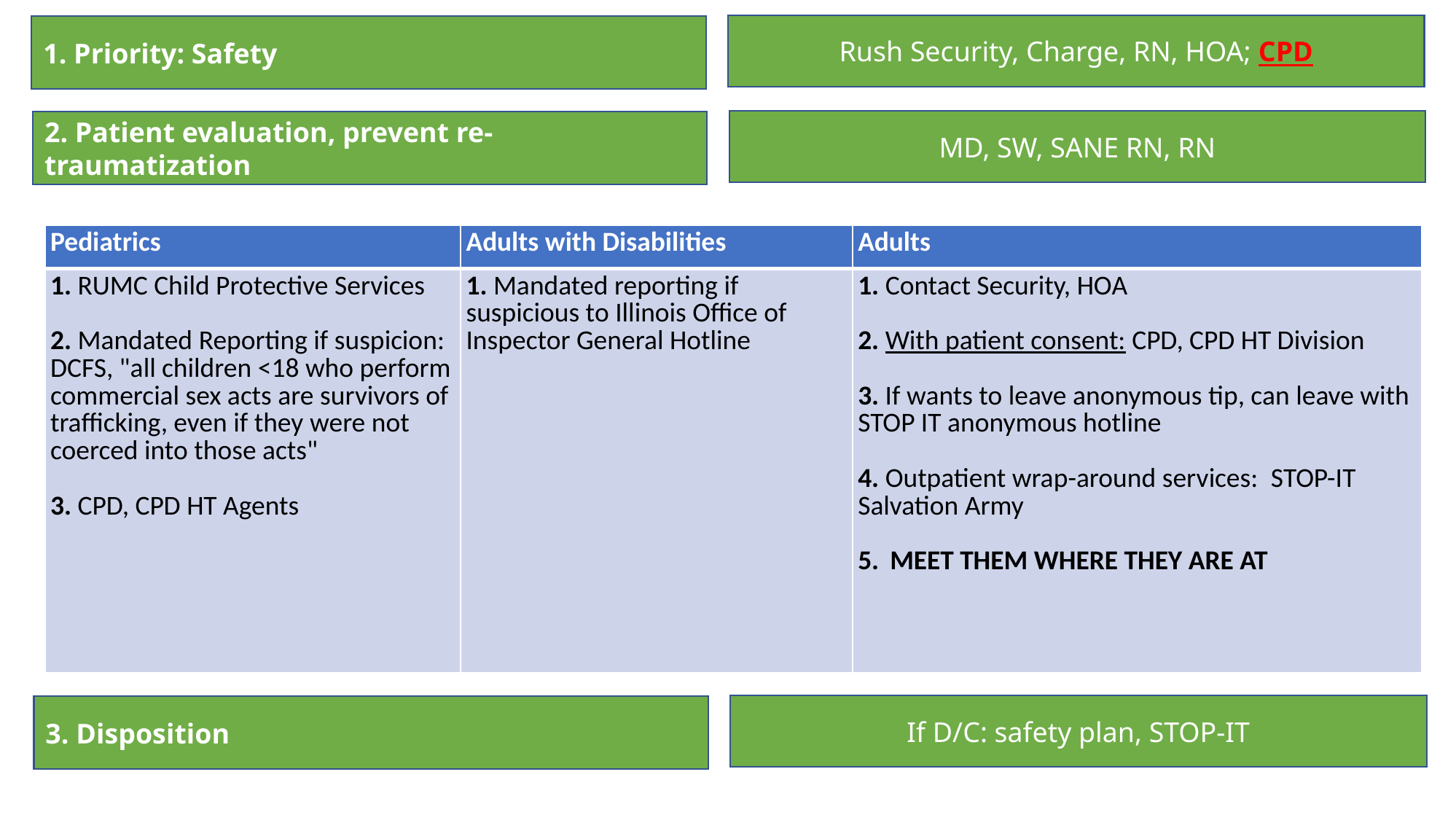

Rush Security, Charge, RN, HOA; CPD
1. Priority: Safety
MD, SW, SANE RN, RN
2. Patient evaluation, prevent re-traumatization
| Pediatrics | Adults with Disabilities | Adults |
| --- | --- | --- |
| 1. RUMC Child Protective Services 2. Mandated Reporting if suspicion: DCFS, "all children <18 who perform commercial sex acts are survivors of trafficking, even if they were not coerced into those acts" 3. CPD, CPD HT Agents | 1. Mandated reporting if suspicious to Illinois Office of Inspector General Hotline | 1. Contact Security, HOA 2. With patient consent: CPD, CPD HT Division 3. If wants to leave anonymous tip, can leave with STOP IT anonymous hotline 4. Outpatient wrap-around services:  STOP-IT Salvation Army 5.  MEET THEM WHERE THEY ARE AT |
If D/C: safety plan, STOP-IT
3. Disposition

## Slide 40
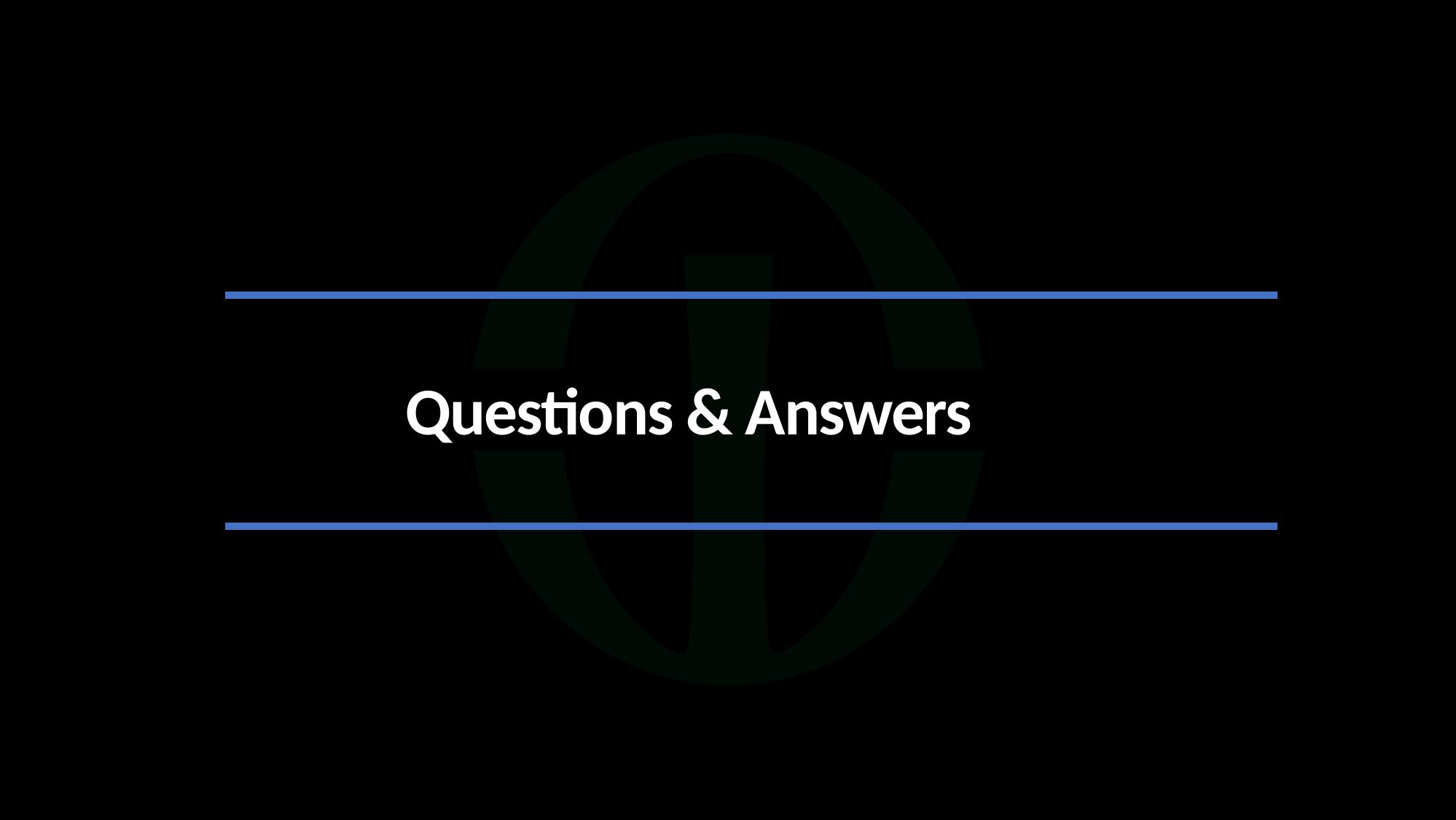

Questions & Answers

## Slide 41
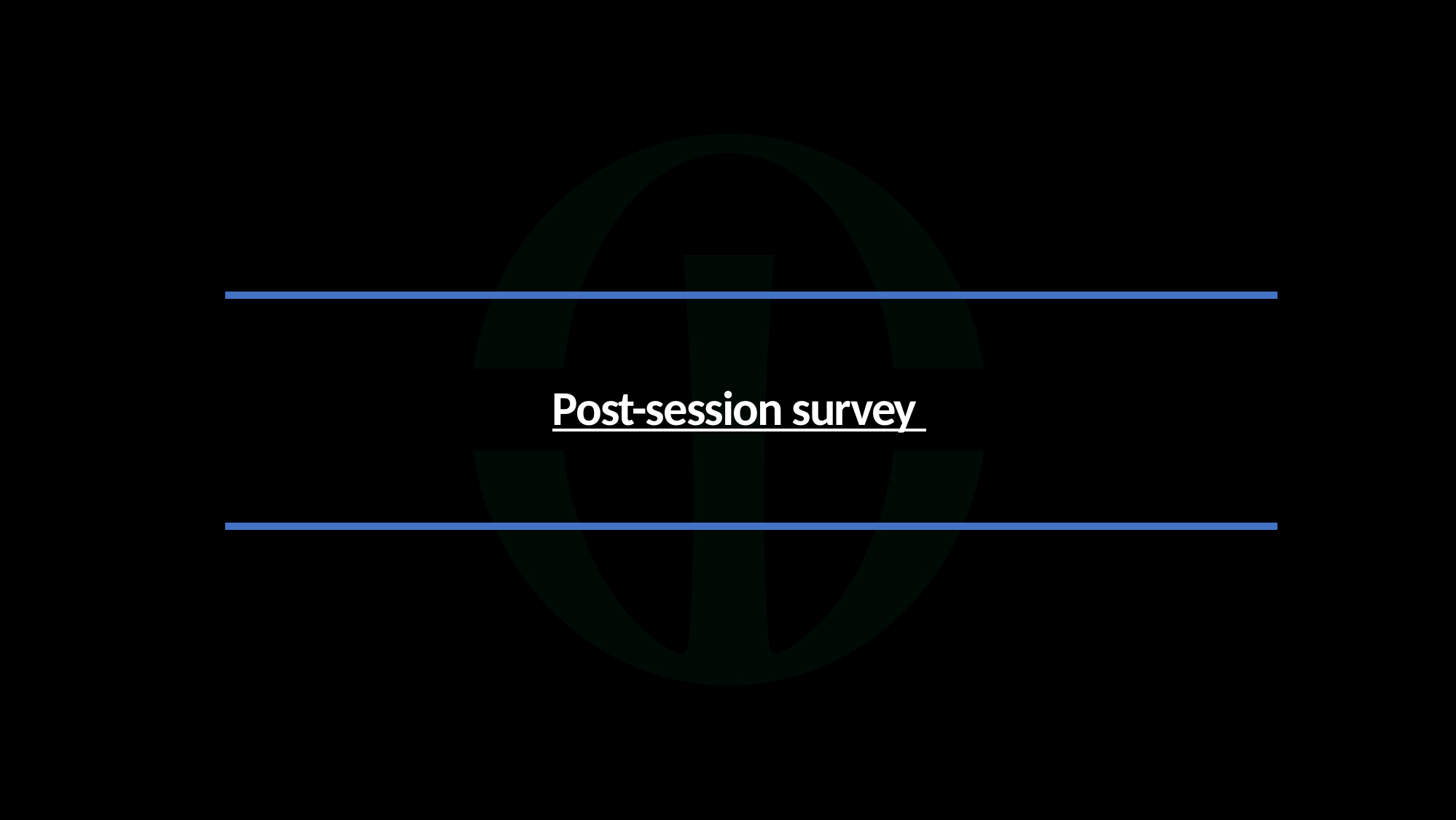

Post-session survey
