# Supplementary material for: Addressing a Gap in Medical School Training: Identifying and Caring for Human Trafficking Survivors Using Trauma-Informed Care
Source: MedEdPORTAL. 2023 Mar 14;19:11304. doi: 10.15766/mep_2374-8265.11304 (PMC10011204; doi:10.15766/mep_2374-8265.11304)
Supplement: Supplementary file 1 — Didactic Lecture.pptxFacilitation Guide.docxStudent Worksheet Without Answers.docxStudent Worksheet With Suggested Answers.docxTool Kit.docxPre- and Postsession Survey Questions.docxExtra Scenarios.docx [file mep_2374-8265.11304-s001.zip › C. Student Worksheet Without Answers.docx]

**PATIENT VIGNETTE**

You are currently a fourth-year medical student participating in your emergency medicine rotation. Your next task is to collect a history from a new patient: Max, a 20-year-old male who appears younger than his stated age. He is accompanied by an adult male, who will not let Max leave his sight. Max presents with multiple shallow wounds on his back and bruises in multiple different stages of healing. Many of the wounds look infected. He is disheveled and avoids eye contact with you. You begin to ask Max some questions, but he remains silent and allows the accompanying man to answer. The visitor states that Max fell while at work.

1. **What are some red flags for human trafficking in this scenario?**
2. **Do you think it’s appropriate and/or important to get Max alone to ask him some questions? Why or why not?**
3. **If so, how may you do that?**

You were able to get Max alone.

1. **What kinds of questions would you ask? How would you utilize trauma-informed practices in this scenario?**
2. **What are some inappropriate questions or things people may say in an attempt to be empathetic that are inappropriate and re-traumatizing?**

After building rapport with Max, he discloses that he entered "the life" (a reference to the commercial sex industry) to get by. He explains that he came out to his parents when he was 15 years old, thinking they would be accepting, but instead, they alienated him. He ran away from home and was quickly recruited into "the life." Max states that when he first met his trafficker, he promised to give him a job and provide food and housing for him. The trafficker did provide food and housing, but Max never received any money for his work.

1. **How do you appropriately respond, utilizing trauma-informed care practices?**
2. **What are some examples of inappropriate responses?**

Max abruptly stops the conversation and appears acutely anxious and frightened. "I need to get back. Where is he (trafficker)? I want to see him."

1. **So, Max may not be able to leave his trafficking situation. Why might this be?**
2. **What can you do to plan for when he is ready to leave safely?**
