# Supplementary material for: Addressing a Gap in Medical School Training: Identifying and Caring for Human Trafficking Survivors Using Trauma-Informed Care
Source: MedEdPORTAL. 2023 Mar 14;19:11304. doi: 10.15766/mep_2374-8265.11304 (PMC10011204; doi:10.15766/mep_2374-8265.11304)
Supplement: Supplementary file 1 — Didactic Lecture.pptxFacilitation Guide.docxStudent Worksheet Without Answers.docxStudent Worksheet With Suggested Answers.docxTool Kit.docxPre- and Postsession Survey Questions.docxExtra Scenarios.docx [file mep_2374-8265.11304-s001.zip › F. Pre- and Postsession Survey Questions.docx]

We used Microsoft Office Forms to create surveys with these questions which we linked to a QR code and displayed on PowerPoint for participants to access the survey before and after the session.

Pre-Session Survey

1. Unique identifier (ID number (Initials/Month of Birthday/Day): example, Mary Smith 12/15/91, (MS1215)
2. What residency are you applying to? **(Drop-down question)**
3. During your medical school clinical training have you ever suspected that any of your patients may have been trafficked?
   1. Yes
   2. No
4. Have you had any formal education on red flags of Human Trafficking?
   1. Yes
   2. No
5. Have you had any formal education on trauma-informed care?
   1. Yes
   2. No
6. Please indicate your level of comfort regarding the following, where:

*1 indicates “very uncomfortable,”*

*2 indicates “slightly uncomfortable,”*

*3 indicates “neither uncomfortable nor comfortable,”*

*4 indicates “slightly comfortable,”*

*5 indicates “very comfortable.”*

a) I am aware of the issue of human trafficking in the United States.

b) I understand what could make someone vulnerable to being trafficked.

c) I feel comfortable with what screening questions to ask to facilitate a disclosure and how to ask them sensitively.

d) I am confident in my ability to respond in a trauma-informed, patient centered way to disclosures of human trafficking.

e) I feel able to safety plan with a patient who is not yet ready to leave their human trafficking situation.

f) I know what situations call for mandated reporting of human trafficking.

g) I know who to call should my patient be ready to leave their human trafficking situation.

h) I am aware of the red flags of human trafficking.

1. What are you hoping to learn from this session? **(Free response)**

Post-Session Survey

1. Unique identifier (ID number (Initials/Month of Birthday/Day): example, Mary Smith 12/15/91, (MS1215)
2. Please indicate your level of comfort regarding the following, where:

*1 indicates “very uncomfortable,”*

*2 indicates “slightly uncomfortable,”*

*3 indicates “neither uncomfortable nor comfortable,”*

*4 indicates “slightly comfortable,”*

*5 indicates “very comfortable.”*

a) I am aware of the issue of human trafficking in the United States.

b) I understand what could make someone vulnerable to being trafficked.

c) I feel comfortable with what screening questions to ask to facilitate a disclosure and how to ask them sensitively.

d) I am confident in my ability to respond in a trauma-informed, patient centered way to disclosures of human trafficking.

e) I feel able to safety plan with a patient who is not yet ready to leave their human trafficking situation.

f) I know what situations call for mandated reporting of human trafficking.

g) I know who to call should my patient be ready to leave their human trafficking situation.

h) I am aware of the red flags of human trafficking.

1. How satisfied are you with this training? **(1-5)**
2. Would you recommend this to a classmate at another medical school? **(1-5)**
3. How relevant is this training to your future role as a resident physician? **(1-5)**
4. Do you think this should be part of the required medical school curriculum at Rush?
   1. Yes
   2. No
5. Please rate the brief didactic session in the beginning. **(1-5)**
6. Please rate the small group sessions. **(1-5)**
7. What did you like the most about this training? **(Free response)**
8. What are the opportunities for improvement for this training? **(Free response)**
9. Any other comments? **(Free response)**
