# Supplementary material for: Addressing a Gap in Medical School Training: Identifying and Caring for Human Trafficking Survivors Using Trauma-Informed Care
Source: MedEdPORTAL. 2023 Mar 14;19:11304. doi: 10.15766/mep_2374-8265.11304 (PMC10011204; doi:10.15766/mep_2374-8265.11304)
Supplement: Supplementary file 1 — Didactic Lecture.pptxFacilitation Guide.docxStudent Worksheet Without Answers.docxStudent Worksheet With Suggested Answers.docxTool Kit.docxPre- and Postsession Survey Questions.docxExtra Scenarios.docx [file mep_2374-8265.11304-s001.zip › G. Extra Scenarios.docx]

**Scenario 1**

S.M is a 26 y/o female who comes to the Emergency Department (ED) for a one-month h/o increased vaginal discharge and pelvic pain. She does not speak English well, and the patient’s boyfriend offers to act as the interpreter. During the interview, S.M is nervously shifting her gaze between you and her boyfriend. After taking cervical cultures for gonorrhea and chlamydia, the clinician makes a presumptive diagnosis of pelvic inflammatory disease (PID) and gives S.M. a prescription for antibiotics. On chart review, it appears S.M has been diagnosed with several sexually transmitted infections (STIs) at various EDs in the area over the past several years. The clinician explains the serious nature of PID to S.M. and the importance of having her sexual partner(s) tested. The patient is given an appointment to follow up in an outpatient clinic. The clinician thanks the boyfriend for interpreting, and S.M. leaves the clinic with him. The following week, S.M. does not show up for her scheduled outpatient visit.

**Scenario 2**

D.G is a 31 y/o woman who presents to the ED accompanied by a 29 y/o female friend. Neither of the women have forms of identification. The patient reports she and her friend got into a fight last night and her friend punched her in the face. She denies wanting to press charges against this friend. On exam, there is ecchymosis around bilateral eyes and an obvious deformity of the nasal bridge. You also notice various bruises on the patient’s neck and upper extremities; some seem to be from days ago, others from even longer. Throughout the visit, the patient and her friend avoid eye contact with the male nurse and male physician.

**Scenario 3**

J.S is a 20 y/o male who presents to the ED alone, late at night, with identification that shows he is from Louisiana. He complains of an injury to his rectum after falling off his bicycle. He appears younger than his stated age, and he is disheveled and dirty. You are concerned he is using drugs and is possibly homeless. When asked, he reports occasionally using methamphetamine intravenously. Upon further questioning about his injuries, he becomes aggressive and lashes out at the staff.

**Scenario 4**

A.O is a 48 y/o male who presents to the ED with a painful reddened blister on his left forearm. The manager from the restaurant where the patient is employed is on the phone on speaker. The patient does not speak English, and his manager explains that the patient burned his arm on a hot dishwasher. By this point, you get a licensed medical interpreter and hang-up the phone with the manager. The patient appears frightened and is hesitant to answer questions, but ultimately explains he is from Venezuela and moved to Chicago a few years ago for work. On exam, you see a few other healing burns visible on his right arm. A physician assistant working in the ED orders a tetanus booster and antibiotic ointment.

**Scenario 5**

It is 1 AM in a single coverage ED when a 17 y/o female presents complaining of stomach pain and desiring a pregnancy test. The ED is busy that day and the patient repeatedly asks how long this will take. After an hour of waiting, she pleads with staff saying she “really needs to get back,” and she continues to look at the clock in the room and appears anxious. When the staff try to ask her other questions about her history, she responds with one-word answers. She appears exhausted. Eventually the patient becomes impatient and leaves without the test results.
