# Supplementary material for: Addressing a Gap in Medical School Training: Identifying and Caring for Human Trafficking Survivors Using Trauma-Informed Care
Source: MedEdPORTAL. 2023 Mar 14;19:11304. doi: 10.15766/mep_2374-8265.11304 (PMC10011204; doi:10.15766/mep_2374-8265.11304)
Supplement: Supplementary file 1 — Didactic Lecture.pptxFacilitation Guide.docxStudent Worksheet Without Answers.docxStudent Worksheet With Suggested Answers.docxTool Kit.docxPre- and Postsession Survey Questions.docxExtra Scenarios.docx [file mep_2374-8265.11304-s001.zip › B. Facilitation Guide.docx]

**Faculty Facilitation Guide**

Identification of and Care for Survivors of Human Trafficking: A Trauma-Informed Care Approach

**Objectives**

By the end of this session, students will be able to:

1. By the end of this activity, learners will be able to:
2. Discuss the prevalence of human trafficking and the associated vulnerability factors.
3. Identify the major red flags of human trafficking in a clinical setting.
4. Establish comfort in asking appropriate screening questions to identify human trafficking.
5. Respond with increased confidence to human trafficking disclosures using trauma-informed practices (including use of appropriate language).
6. Describe medical care options, safety planning, and state mandated reporting guidelines in the context of human trafficking.

**Email Communication Pre- and Post-Session**

- Email students beforehand (Two days prior to session):
  - Didactic lecture slides
  - Student worksheet without answers
- Email facilitators beforehand (One week prior to session):
  - Didactic lecture slides
  - Facilitation guide
  - Student worksheet with sample answers
  - Tool kit
  - *Encourage facilitators to periodically ask the students how they are doing and encourage self-care after the session
- Email students post-training (One day following session):
  - Tool kit
  - Student worksheet with sample answers

**Timeline/Agenda**

| Time to complete and personnel required | Content to be covered | Resources needed |
| --- | --- | --- |
| 5 minutes | Pre-session survey  Introduction to training (including a trigger warning) | Pre-session survey link or QR code |
| 20 mins  Speaker options: Expert on human trafficking (SANE nurse, local nonprofit social worker that specializes in interacting with human trafficking survivors, physician, RN, PA, NP) | **Intro of Human Trafficking**  -Prevalence  -Implicit bias  -Myths and misconceptions  -Vulnerability factors  -Red flags | Didactic lecture slides |
| 10 mins  Speaker: Psychiatrist or clinician who practices trauma-informed care | **Intro to Trauma-Informed Care**  -Effects of trauma  -Principles of Trauma-Informed care  -Use of appropriate language  -History taking skills  -Examination skills | Didactic lecture slides |
| 70 minutes  Co-facilitators:  -Expert speakers | Small group instruction for activity:   1. Identify a recorder 2. Encourage participation from all group members 3. Move into pre-set groups (six-eight) to work on clinical vignette questions   First 20 minutes:  Students discuss clinical vignette in small groups with expert speakers entering rooms to facilitate discussion and answer questions  Remaining 50 minutes: Students return to large group to discuss with expert speakers  *Consider taking a break part way through to encourage self-care techniques | Break out room groups  Student worksheet (Clinical Vignette) |
| 15 mins  Speaker options: Expert on human trafficking (SANE nurse, local nonprofit social worker that specializes in interacting with human trafficking survivors, physician, RN, PA, NP) | Mandated reporting  Hospital protocol (if available)  Discussion of self-care  Q&A  Post-session survey | Post-session survey link or QR code |

**Pre-Session Survey**:

- Find the pre-session survey questions attached
- Make your own online survey form and send the link to the pre-session survey to students
- Ask students to make an identifying code they will use for pre-session and post-session survey

**Introductory Didactic Lecture (30 minutes)**

- Find attached lecture slides
- Students will listen to expert speaker(s) with the objective of educating the students on human trafficking prevalence, vulnerability factors, potential red flags in a clinical setting, and trauma-informed care including the use of appropriate language, history taking skills, and examination skills.

**Student Worksheet (Clinical Vignette) (70 minutes)**

- Students will be provided with the student worksheet with one clinical vignette followed by four discussion prompts. The subject of the vignette will be a young adult LGBTQ+ male who presents to the emergency department. The vignette will walk the students through a progression of clinical situations and ask the students to reflect on how they would navigate each presenting situation.
- There are four total discussion questions regarding this patient. The students will be split into groups of six-eight and will be allotted 20 minutes to discuss each prompt before entering back into the large group to debrief.
- When it is time to debrief each prompt, facilitators with experience and training in caring for survivors of human trafficking in a trauma-informed manner will debrief each scenario. They will provide insight on language, history-taking skills, and physical exam skills meant to avoid re-traumatizing these patients.
- **Note**: Please explain to students that this activity would not be approached any differently with human trafficking survivors with different gender and sexuality identities. This worksheet focuses on a young LGBTQ+ male because (a) the LGBTQ+ community is at disproportionately elevated risk for human trafficking and because (b) males are underrepresented as survivors in human trafficking education and training.
- The student worksheet (clinical vignette) is complete with sample answers, attached in the appendices (Appendix C: worksheet without answers; Appendix D: worksheet with answers).

**Additional question prompts for facilitators after debriefing the scenario as a large group**

- Can you think of any additional red flags not presented in this case?
- Can you think of any cases you have seen clinically that may have shown concerning signs and symptoms for human trafficking?
- How may implicit bias be at work in this scenario?
- If this were a female patient and you had to perform a pelvic exam, how would you do this using trauma-informed care practices?

**State Mandated Reporting and General Protocol at your Hospital or Clinic (15 minutes)**

- Educate students on cases in which mandated reporting is indicated in your state. Consider if you have the following requirements for mandated reporting:
  - Patients <18 years old
  - Patients with disabilities
  - Elderly patients
- Speak about your hospital or clinic human trafficking protocol (if available)
  - If no protocol is available, utilize a protocol from another organization as an example for what could be done
- Include National Human Trafficking Resource Center (NHTRC) protocol

**Facilitator Wrap-Up (15 minutes)**

- Motivate students by encouraging them to be a self-starter at the hospital in developing a human trafficking protocol at their future places of work.
- Leave time to answer questions the students may have; elicit feedback and reinforce major takeaways from the session
- Encourage self-care following the session and provide resources for available counseling services and text-line

**Post-session survey**

- Find the post-session survey attached
- Send the link to the post-session survey to students
- Ask students to use the same identification code that they used for the pre-survey

**Resources for Facilitators**

- <https://polarisproject.org/>
- <https://humantraffickinghotline.org/>
- <https://www.traumainformedcare.chcs.org/what-is-trauma-informed-care/>
- <https://healtrafficking.org/>

**Resources**

1. National Human Trafficking Resource Center (NHTRC) protocol
   1. https://humantraffickinghotline.org/resources/framework-human-trafficking-protocol-healthcare-settings
