# Supplementary material for: Addressing a Gap in Medical School Training: Identifying and Caring for Human Trafficking Survivors Using Trauma-Informed Care
Source: MedEdPORTAL. 2023 Mar 14;19:11304. doi: 10.15766/mep_2374-8265.11304 (PMC10011204; doi:10.15766/mep_2374-8265.11304)
Supplement: Supplementary file 1 — Didactic Lecture.pptxFacilitation Guide.docxStudent Worksheet Without Answers.docxStudent Worksheet With Suggested Answers.docxTool Kit.docxPre- and Postsession Survey Questions.docxExtra Scenarios.docx [file mep_2374-8265.11304-s001.zip › D. Student Worksheet With Suggested Answers.docx]

Patient Vignette with Suggested Answers - Identification of and Care for Survivors of Human Trafficking: A Trauma-Informed Care Approach

You are currently a fourth-year medical student participating in your emergency medicine rotation. Your next task is to collect a history from a new patient: Max, a 20-year-old male who appears younger than his stated age. He is accompanied by an adult male, who will not let Max leave his sight. Max presents with multiple shallow wounds on his neck, arms, and legs, along with bruises in multiple different stages of healing. Many of the wounds look infected. He is disheveled and avoids eye contact with you. You begin to ask Max some questions, but he remains silent and allows the accompanying man to answer. The visitor states that Max fell while at work.

1. **What are some red flags for human trafficking in this scenario?**

- *Red flags for human trafficking:*
  - *Accompanied by another person who is speaking on his behalf*
  - *Multiple wounds in different stages of healing*
  - *Appears younger than stated age (could he be a minor?)*
    - *Note that in these situations, the patients may not have identification available, and in this case, we cannot confirm if the patient is a minor or not.*
  - *Appearance – disheveled and avoiding eye contact*
  - *Wounds seem to be incongruent with stated mechanism of “falling at work”*

1. **Do you think it’s appropriate and/or important to get Max alone to ask him some questions? Why or why not?**

- *It is very important that you get Max, and other patients you may suspect to be experiencing human trafficking, alone to ask them questions.*
  - *Patient autonomy is very important in these cases. With the potential trafficker in the room, Max does not have full autonomy to share his story, express his concerns, or ask questions. All patients deserve the opportunity to be honest and candid in a safe environment -- especially if you have high suspicion for human trafficking or other kinds of abuse.*
  - *If you get the patient alone and ask them about their situation and they cannot disclose this time, at least you gave them the opportunity to and make it clear that you care about their health and safety. This might make the patient feel like they can come back to your care when it is safer for them to leave their situation.*
- *While we do suggest that the students attempt to get the patient alone to ask further questions, here are some valid points students may bring up in support of not getting the patient alone:*
  - *Asking to see the patient alone may compromise the patient’s safety.*
  - *More likely than not, the patient will be leaving your care to go home with the trafficker, so it is important that you ensure the patient’s safety when they do leave your care.*

1. **If so, how may you do that?**

- *Normalizing the institution’s procedure of interviewing or examining the patient alone.*
- *Tell the patient and their visitor that you must do further imaging to get patient alone.*
- *Depending on the scenario, you can say something along the lines of “It’s hospital policy for this exam to be done in private.”*
- *At some institutions across the country, they are employing a “blue sticker” program, where blue stickers are left in the emergency department bathrooms and patients are encouraged to place a sticker on their urine cup if they feel unsafe and want to talk to staff about it.*

You were able to get Max alone.

1. **What kinds of questions would you ask? How would you utilize trauma-informed practices in this scenario?**

- *Trauma-informed care (and good practices in general):*
  - *Preface the conversation with the fact that the patient has the right to not answer any question that they do not want to / are not comfortable answering.*
    - *Reliving details can be extremely re-traumatizing, so it is important that you do not make the patient tell their story if they are not ready.*
  - *Make sure the patient feels safe and comfortable.*
    - *Simple suggestions such as sitting up the bed to a comfortable position, asking if temperature in the room is okay, etc. can convey that you care about them.*
    - *Try to sit at eye level with the patient.*
    - *Make it a point to let the patient know you are invested in their comfort/safety.*
- *Appropriate screening questions:*
  - *Verbalize “Disclosure is a process.”*
    - *It is important to realize this and to never push a patient to disclose the details of their situation. This should be on their own terms, and you should verbalize from the start that while you’ll be asking questions, they should share only to the level that they feel comfortable.*
  - *Try to ask open-ended questions.*
    - *“Is there anything that concerns you about your own safety?”*
    - *“Has there been anything on your mind that is concerning you?”*
    - *“Tell me about your living situation. Do you know where you will sleep every night? Do you feel like you can leave when you want?”*
    - *“What is your work like?”*
    - *“Tell me about this tattoo.” (If the patient presents with one.)*
    - *“How did you get this scar?” (If the patient presents with one.)*
    - *“Has anyone hit you or hurt you today? This week?”*
  - *Don’t be afraid to ask more direct questions when your suspicion is high.*
    - *“Have you ever traded sex for money, drugs, food or to avoid getting hurt?”*
      - **Note that with these yes/no questions, it is very easy for the patient to just say “no.”*
      - **If the patient does say “no,” consider asking:*
        - *“Do you know anyone else who has experienced these things?”*
        - *“Are you living with or working with anyone this has happened to?”*
        - *This normalizes the situation and can function as a foot in the door, a chance to build rapport, a chance to gain some insight into what the patient’s situation is.*
    - *“Has anyone ever forced you to work without fair payment or lied to you about your work conditions?”*
    - *“Can you leave your job or work situation if you want to?”*
    - *“Have you felt threatened or trapped either recently or in the past by anyone?”*
    - *“Working in the emergency department (or wherever you work), I see many patients who have experienced violence... have you ever experienced violence?”*
      - *You can make it clear that you ask all patients this question and you are not just asking them because they look or act a certain way. This can reduce the patient’s sense of feeling judged.*
  - *Sample questions from National Human Trafficking Adult Screening Tool*
    - **Notice that these questions start off with “Sometimes people...” - it can be helpful to somewhat normalize the patient’s suspected situation to make them feel more comfortable talking about it.*
    - *“Sometimes people work for someone or spend time with someone who does not let them contact their family, spend time with their friends, or go where they want when they want. Have you ever experienced this, or are you in a situation where you think this could happen?”*
    - *“Sometimes people live where they work or where the person in charge tells them to live, and they’re not allowed to live elsewhere. Have you ever experienced this?”*
    - *“Sometimes people do unfair, unsafe, or even dangerous work or stay in dangerous situations because if they don't, someone might hurt them or someone they love. Have you ever experienced this?”*
    - *“Sometimes lies are used to trick people into accepting a job that doesn’t exist, and they get trapped in a job or situation they never wanted. Have you ever experienced this, or are you in a situation where you think this could happen?”*
    - *“Sometimes people make efforts to repay a person who provided them with transportation, a place to stay, money, or something else they needed. The person they owe money to may require them to do things if they have difficulty paying because of the debt. Have you ever experienced this, or are you in a situation where you think this could happen?”*
  - *Importantly, continuing to ask these questions to all your patients in your practice will help to normalize this for you as a healthcare worker.*

1. **What are some inappropriate questions or things people may say in an attempt to be empathetic that are inappropriate and re-traumatizing?**

- *Inappropriate comments and questions:*
  - *“How did you come to be in this situation?”*
  - *“Why don’t you just leave?”*
  - *“Why has it taken you so long to come in?”*
  - *“Don't worry we’ll get you out.”*
    - *This can be perceived as victim blaming.*
  - *“I’m assuming you were forced into this?”*
    - *Human trafficking is so much more complicated than being “forced into it.” It’s complicated by emotional, psychological, financial manipulation and dependence.*
    - *Individuals experiencing trafficking may not at this point see it as being “forced onto them,” so it is inappropriate to label it as that for them without them making that claim themselves.*

After building rapport with Max, he discloses that he entered "the life" (a reference to the commercial sex industry) to get by. He explains that he came out to his parents when he was 15 years old, thinking they would be accepting, but instead, they alienated him. He ran away from home and was quickly recruited into "the life." Max states that when he first met the man who is accompanying him to the emergency department today, the man promised to provide food and housing for him. This man did provide food and housing, but he now asks Max to perform sexual acts in return.

1. **How do you appropriately respond, utilizing trauma-informed care practices?**

- *Thank and acknowledge Max for sharing.*
- *Empathize with the patient*
  - *Note that calling the situation “hard” or “traumatic” may be inappropriate – it is important to mirror how the patient is feeling about the situation at this stage.*
- *Reassure Max that this is a confidential conversation and that you will not share anything unless he chooses to report.*
- *Ask Max where he would like to go from here.*
  - *Important to give the patient autonomy and agency.*
- *Let him know that we can connect him to resources if he wants that, and that he has options for when he is ready.*
- *Use the patient’s language.*
  - *If they call it “the life,” you call it “the life.” If they call it “trafficking,” you call it “trafficking.” Labeling it yourself can be re-traumatizing.*
- *Be aware of your own emotional triggers and how they could come up in this patient example or others like it.*
  - *If you have to step out to take care of yourself, do that.*
  - *You have to take care of yourself to be able to take care of these patients.*
- *Importantly, once the patient discloses their story, do not make the patient retell it.*
  - *The staff member who is there for the disclosure should be the point of contact and convey the sensitive situation to the rest of the staff – retelling the story can be very re-traumatizing.*

1. **What are some examples of inappropriate responses?**

- *Do not say:*
  - *“You poor thing.”*
    - *This is very demeaning.*
  - *“I know how you feel.”*
    - *Unless you yourself are a survivor of human trafficking, you do not precisely know how they feel. Even if you are a survivor of human trafficking, everyone’s situation is different, so it’s inappropriate to assume you know how they feel.*
- *Do not make assumptions about how the patient may feel.*
  - *The patient may have a relationship with the trafficker, so it is inappropriate to speak negatively about that person.*
  - *Do not assume that the patient can leave their situation – they know their situation better than you and if it is too dangerous for them to leave at that moment*
- *Do not use language like "victim" as often individuals will not identify themselves as "human trafficking victims."*
  - *Some patients may not be aware that they are in an abusive situation or that their situation may be classified as “human trafficking.”*
  - *Again, use the patient’s language.*

Max abruptly stops the conversation and appears acutely anxious and frightened. "I need to get back. Where is he (the trafficker)? I want to see him."

1. **So, Max may not be able to leave his trafficking situation. Why might this be?**

- *It is essential to understand that you cannot force a patient to leave their trafficking situation. Individuals will leave when they are able and ready to. In this example, Max may have formed a bond with his trafficker and feels like this is home for him.*
- *It can also be dangerous and even life threatening for individuals to attempt to leave their situation. This is a decision that Max must make on his own.*
- *He may be dependent financially, emotionally, etc.*
  - *The trafficker may be in control of the patient’s finances. They may have control over the patient’s identification documents. They may be the patient’s only source of food and shelter.*
- *This may seem less complicated or better than navigating homelessness, etc. once leaving.*
  - *In Max’s case, he may feel that the trafficker “saved” him from his family who denied him and kicked him out of the house.*
  - *There may be a “fear of the unknown” - fear of what life will be like if he leaves.*
- *It is important to recognize small wins in each encounter.*
  - *It may be that the patient went home with their trafficker today, but the "win” may be that you had a comfortable conversation where the patient seemed to feel safe. A "win" may be that you’ve seen the patient before and at this visit they allowed you to talk to them about resources (assuming last time they did not see the need for this and refused this).*

1. **What can you do to plan for when he is ready to leave safely?**

- *You need to approach this from a harm-reduction framework.*
  - *Goal: make the patient feel safe enough to return to the ED again in the future.*
  - *Clinicians should explicitly state that the patient is welcome to return for clinical care in the future or for assistance in seeking safety.*
  - *Try to gauge what the patient needs and what stage they are in the process of making a change. They may not feel comfortable/ready to leave the situation.*
  - *Don’t assume you have any understanding of their situation and possible consequences of leaving.*
  - *Transfer power of care back into their hands and show them they have agency to make this decision.*
    - *Ask the patient what you can do to best help them.*
  - *Offer them connection to or information about resources before he needs to leave.*
    - *A phone call can be made to the National Trafficking Hotline together in person.*
    - *You can offer to hide the digits of the phone number in the discharge paperwork in an inconspicuous manner or offer to help him memorize the hotline numbers while he is there.*
      - *You can be creative with the discharge paperwork.*
      - *Some institutions give their patients inconspicuous Chapstick bottles, where when they open it, it has the number to the National Trafficking Hotline.*
  - *If a patient makes it known that they want to leave the situation, assure safety on site first.*
    - *Call the National Human Trafficking Hotline and follow their guidance on next steps.*
    - *In the meantime, work with security in an inconspicuous way to ensure safety to you and the patient.*
