# Supplementary material for: Addressing a Gap in Medical School Training: Identifying and Caring for Human Trafficking Survivors Using Trauma-Informed Care
Source: MedEdPORTAL. 2023 Mar 14;19:11304. doi: 10.15766/mep_2374-8265.11304 (PMC10011204; doi:10.15766/mep_2374-8265.11304)
Supplement: Supplementary file 1 — Didactic Lecture.pptxFacilitation Guide.docxStudent Worksheet Without Answers.docxStudent Worksheet With Suggested Answers.docxTool Kit.docxPre- and Postsession Survey Questions.docxExtra Scenarios.docx [file mep_2374-8265.11304-s001.zip › E. Tool Kit.docx]

**TOOL KIT**

**Red Flags of Human Trafficking**

| **Triage** | **History** | **Physical Exam** |
| --- | --- | --- |
| Accompanied by another person who is speaking on their behalf or holding their documents | Fearful, reluctant to make eye contact, reluctant to share information | Pan-positive ROS  *complaints across several organ systems can be a sign of trauma |
| Appears younger than stated age | Inconsistent history or unexplained delay in seeking care | Bruising, burns, or scars in various stages of healing |
| No ID, insurance, and/or unsure of address/“just visiting the area” | Housing or financial instability | Inappropriate attire  *example: winter coat in summer |
| Inconsistent health records | Frequent testing or treatment for STI/UTI, multiple pregnancies/abortions | Malnourishment |
| Unaware of location, date, and/or time | Substance use or withdrawal | Suspicious tattoos or evidence of branding |
| Delayed presentation for injury or illness |  | Occupational injuries |

**Dos & Don'ts: Trauma-Informed Care Delivery**

| Language | Do   - Mirror the language that is being used by the patient   - For example, don’t use the words “human trafficking,” “prostituting,” or “abuse” unless the patient uses them first   Don’t   - Don’t use the word “victim” -- patients may not see themselves as victims. Instead use the word patient. |
| --- | --- |
| History Taking Skills | Do   - Make sure the patient feels safe and comfortable   - Simple offerings to reposition the bed to a comfortable position, asking if temperature in the room is okay, etc. can convey that you care about them   - Try to sit at eye level or lower with the patient - Always give the patient the opportunity to answer questions in private   - With the potential trafficker in the room, the patient does not have full autonomy to share their history, express their concerns, or ask questions. All patients deserve the opportunity to be honest and candid in a safe environment   - Depending on the situation, you can say something along the lines of “Hospital requirement for a private exam” or “Need for the patient to be seen for radiology. No one else is allowed in the room because of radiation"   - Try to maintain patient’s safety while doing this   Don’t   - Don't make promises   - Don’t say “we’re going to get you out of this situation and all your problems will be solved.” Instead, ask the patient what is most appropriate for their safety at this time. The patient is the only person who can determine what is best. |
| Screening Questions | Do   - Preface the conversation by letting the patient know that they have the right to not answer any questions that they do not want or are not comfortable answering - Ask open ended questions   - Can you tell me about your living situation?   - What is your work like?   - Is there anything that concerns you about your own safety?   - Has there been anything on your mind that is concerning to you? - Feel confident asking more direct questions when your suspicion is high   - “Have you ever traded sex for money, drugs, food, or to avoid getting hurt?”     - If the patient says “no,” consider asking “do you know anyone who has?” or “are you living with or working with anyone this has happened to?”   - It can be helpful to attempt to normalize the situation and start your question with “Sometimes people...”, for example:     - “Sometimes people work for someone or spend time with someone who does not let them contact their family, spend time with their friends, or go where they want when they want. Have you ever experienced this, or are you in a situation where you think this could happen?”     - “Sometimes people live where they work or where the person in charge tells them to live, and they’re not allowed to live elsewhere. Have you ever experienced this?”     - “Working in the emergency room I have treated many people who have experienced violence, so I ask all my patients about their safety. Do you feel you are safe from harm or threats?”   Don’t   - Do not victim blame   - Do not ask “How did you come to be in this situation?”, “Why don’t you just leave?” or “Why has it taken you so long to come in?” - Do not forget how complex human trafficking is   - Do not say “I’m assuming you were forced into this.” Human trafficking is much more complicated than being “forced into it.” It is complicated by emotional, psychological, financial, and other forms of manipulation and dependence.   - Survivors may not see it as “force,” so it is inappropriate to label it as such without them making that statement themselves. |
| Physical Exam Skills | General Trauma-Informed Physical Exam   - Attend to draping and modesty - Introduce exam components and explain why that component is important for their health - Ask permission - Stay within eyesight, never go behind the patient’s back - Respect personal space, typically one arm’s length or more when not directly examining the patient - Use simple, clinical language - Use professional touch when conducting the physical exam - Be efficient - After the exam: Express thanks, discuss results, ask if the patient has any questions   Trauma-Informed GYN Exam   - Allow the patient to keep on any clothing they want - Emphasize the exam will be over whenever the patient requests it to end - Allow the patient to insert speculum and participate in the exam - Bimanual exam should be performed on the side of the patient rather than between the legs of the patient |

**State Mandated Reporting**

- All children <18 who perform commercial sex acts are survivors of sex trafficking
- Adults with disabilities who perform commercial sex acts are survivors of sex trafficking
- Other adults should be met where they are and provided with autonomy

**Resources for You** (Add local resources. Look for local organizations, and state government resources.)

| National Human Trafficking Resource Center | <https://humantraffickinghotline.org/>  24-Hour Hotline: 888-373-7888  Text “HELP” or “INFO”: 233733 SMS |
| --- | --- |
| Polaris Project | <https://polarisproject.org/> |
| Heal Trafficking | <https://healtrafficking.org/> |

Special Recognition to the *Rush University Medical Center Emergency Department Team.*
