# Supplementary material for: Addressing a Gap in Medical School Training: Identifying and Caring for Human Trafficking Survivors Using Trauma-Informed Care
Source: MedEdPORTAL. 2023 Mar 14;19:11304. doi: 10.15766/mep_2374-8265.11304 (PMC10011204; doi:10.15766/mep_2374-8265.11304)
Supplement: Supplementary file 1 — Didactic Lecture.pptxFacilitation Guide.docxStudent Worksheet Without Answers.docxStudent Worksheet With Suggested Answers.docxTool Kit.docxPre- and Postsession Survey Questions.docxExtra Scenarios.docx [file mep_2374-8265.11304-s001.zip › Educational Summary Report_11304.pdf]

MedEdPORTAL® | The AAMC Journal of Teaching and Learning Resources

Original Publication

Open Access

Addressing a Gap in Medical School Training: Identifying and Caring for Human Trafficking Survivors Using Trauma-Informed Care

EmmaRose F. Brennan, Artemis Markopoulos, MA*, Jaclyn Rodriguez, RN, SANE-A, SANE-P, Neeral K. Sheth, DO, Nupur Shah, DO

*Corresponding author: artemis_markopoulos@rush.edu

Abstract

Introduction: Human trafficking (HT) is a substantial public health problem, and health care workers are uniquely positioned to help identify and care for survivors. Despite this fact, few medical schools incorporate HT training using trauma-informed care (TIC) principles into their curricula. We developed a training session to educate medical students on recognizing HT red flags and providing TIC to HT survivors. **Methods:** One hundred twenty-seven fourth-year medical students at Rush Medical College attended a 2-hour session consisting of didactic lectures by expert speakers and participated in a group discussion guided by a clinical vignette. Students completed anonymous pre- and postsession surveys that assessed comfort levels in detecting HT red flags and providing TIC. We used a paired t test to compare pre- and postsession survey responses. **Results:** Ninety-five pre- and postsession surveys were matched with unique identifiers and used for analysis. The results demonstrated significant improvement in all the metrics assessed. **Discussion:** This training significantly improved medical students' comfort in identifying and caring for HT survivors, addressing an especially important gap in medical school education. This training can be implemented at other institutions to further improve awareness and efforts in identifying and caring for HT survivors while avoiding retraumatization.

Keywords
Child Sex Trafficking, Human Trafficking, Labor Trafficking, Sex Trafficking, Patient-Centered Care, Red Flags, Trauma-Informed Care, Case-Based Learning, Communication Skills

Educational Objectives

By the end of this session, students will be able to:

1. Discuss the prevalence of human trafficking (HT) and the associated vulnerability factors.
2. Identify the major red flags of HT in a clinical setting.
3. Establish comfort in asking appropriate screening questions to identify HT.
4. Respond with increased confidence to HT disclosures using trauma-informed practices (including use of appropriate language).
5. Describe medical care options, safety planning, and state-mandated reporting guidelines in the context of HT.

Citation:
Brennan EF, Markopoulos A, Rodriguez J, Sheth NK, Shah N. Addressing a gap in medical school training: identifying and caring for human trafficking survivors using trauma-informed care. *MedEdPORTAL*. 2023;19:11304.
https://doi.org/10.15766/mep_2374-8265.11304

Introduction

Human trafficking (HT), defined as the use of force, fraud, or coercion for the purpose of labor or sexual exploitation, is a substantial and growing public health problem.[1] Per the U.S. Department of State, the annual number of identified HT survivors worldwide increased from 44,758 in 2013 to 118,932 in 2019.[2] This number likely underrepresents the scope of the problem, given the clandestine nature of HT and the fact that individuals affected by HT are often disempowered by their traffickers and thus unable to disclose their situation. HT survivors are at an increased risk for suicidal ideation, depression, posttraumatic stress disorder (PTSD), and substance use, as well as chronic illnesses due to poor nutrition, physical abuse, and poor working conditions.[3-5] Survivors continue to report physical and mental illnesses long after exiting their trafficking situation. Of 86 HT survivors surveyed by Lederer and Wetzel, 96% reported having at least one psychological symptom after experiencing trafficking, while the average number of symptoms was 10.5.[3]

The health care system is uniquely positioned to combat the growing number of those affected by HT and mitigate their

Copyright © 2023 Brennan et al. This is an open-access publication distributed under the terms of the Creative Commons Attribution license.

subsequent psychological and physical trauma. Lederer and Wetzel found that 88% of surveyed survivors had an encounter with a health care provider while they were actively being exploited; many had more than one encounter.[3] Given the significant percentage of HT survivors who intersect with the health care system, it is essential that health care workers are trained on the identification of these survivors. It is also important that when caring for these patients, health care workers employ trauma-informed care (TIC), an approach to care that acknowledges trauma's effect on individuals and their health care outcomes and aims to avoid retraumatization.[6]

Intervention through the health care system can prove successful only if health care workers are trained to identify the red flags of HT and employ patient-centered TIC. Currently, medical schools inconsistently incorporate training on HT or TIC into their curricula. We found two publications in *MedEdPORTAL* that addressed education on both HT and TIC.[7,8] One publication targeted pediatric residents with a focus on child trafficking.[7] The other was tailored to medical students; however, its objective was to prepare them to provide health education to sex trafficking survivors[8] rather than to equip them to identify survivors and care for them clinically. We also found a publication that addressed HT but not TIC,[9] a critical concept given the high rates of PTSD and other mental health problems following the experience of HT. Other *MedEdPORTAL* publications aimed to teach TIC but did so in the context of other public health problems, including sexual assault[10] and childhood adversity.[11] Two others addressed TIC generally, with one focusing on trauma-informed communication skills[12] and the second on trauma-informed physical exam skills.[13] Interestingly, many of these publications[10-13] targeted first- or second-year medical students (one study also included dental students[12]). By contrast, our session targeted fourth-year students. While it is important to expose students to TIC early in their careers, TIC requires advanced communication and physical exam skills; therefore, we believed it was better taught with the foundations of clinical medicine already established.

While there are *MedEdPORTAL* publications addressing HT or TIC, there are few[7,8] that address both HT and TIC and none that aim to educate medical students specifically on how to identify HT survivors in a clinical setting and provide patient-centered TIC. We created this session to address that gap and bring awareness to the growing problem of HT.

Methods

Curricular Context

We administered a virtual Zoom session for fourth-year medical students at Rush Medical College in January 2022. The session was part of the Transition to Residency program, designed to teach outgoing students relevant skills before graduation. We developed the session in collaboration with current medical students, subject matter expert physicians, and the SANE (Sexual Assault Nurse Examiner) Coordinator of the Office of the Illinois Attorney General. The training was held virtually during the COVID-19 pandemic, but the training materials can be used for in-person sessions as well.

Session Materials

The session utilized the following materials:

- Didactic lecture slideshow (Appendix A)[3,14-18]
- Facilitation guide (Appendix B)
- Student worksheet without answers (Appendix C)
- Student worksheet with suggested answers (Appendix D)
- Tool kit (Appendix E)
- Pre- and postsession survey questions (Appendix F)
- Extra scenarios (Appendix G)

Sensitive Topic Awareness

Because HT is a sensitive topic, we distributed the didactic lecture slideshow (Appendix A) and student worksheet (Appendix C) to students before the session to allow them to familiarize themselves with what would be discussed. At the beginning of the session, we emphasized the topic's sensitivity and encouraged the students to take breaks if at any point they felt triggered by the session's content. Additionally, we encouraged self-care after the session and reminded students of the free mental health resources offered by Rush University Medical Center.

Personnel

The didactic component of our session included two lecturers: (1) the SANE Coordinator of the Office of the Illinois Attorney General, who delivered HT background content and informed the students about vulnerability factors and red flags of HT, and (2) a psychiatry attending physician, who spoke about TIC in the context of HT. These two experts helped guide group discussion along with an emergency medicine physician and two medical student facilitators, all with experience in the topic.

Part 1: Didactic Lecture

The didactic lecture (Appendix A) included two parts, the first part focusing on HT and the second on TIC.

The SANE Coordinator of the Office of the Illinois Attorney General delivered a short didactic lecture (Appendix A). The lecture provided a discussion on implicit bias, common HT myths

Copyright © 2023 Brennan et al. This is an open-access publication distributed under the terms of the Creative Commons Attribution license.

and misconceptions, types of trafficking, and the prevalence and scope of the problem. She also spoke about the vulnerability factors and red flags of HT. Red flags were discussed within a clinical context: at patient check-in, during a patient history, and during a patient physical exam. The Salvation Army STOP-IT Initiative Against Human Trafficking (a nonprofit organization in the Chicagoland area serving survivors of HT and providing education on HT) and the Cook County (Chicago and surrounding area) State's Attorney's Office created this portion of the lecture. In the version published here, we have omitted some slides from the original lecture to reduce its length. Our only other modification is the removal of copyrighted images.

The psychiatry attending physician created and delivered a lecture (Appendix A) on the effects of trauma and the importance of TIC. He discussed a six-part approach to TIC, including ensuring safety, trustworthiness, empowerment, collaboration, peer support, and care for cultural, historical, and gender issues.

Part 2: Small-Group Breakout Session

Part 2A: For this segment, lasting approximately 20 minutes, we split students randomly into groups of six to eight and asked them to discuss and complete the student worksheet: a progressive clinical vignette with four discussion prompts detailing a patient presenting to the emergency room and displaying red flags of HT (Appendix C). Our five facilitators rotated through the breakout rooms of medical students to monitor participation, engage students in discussion, and answer questions.

Part 2B: This second segment took approximately 50 minutes. Once students had completed their worksheets, they reconvened in the larger group to discuss their answers to the questions. Our expert facilitators then answered any additional questions. At the end of the discussion, we asked each group to email their worksheet responses for us to review for the purpose of improving the session. After the session, we emailed the students a copy of the student worksheet with sample answers (Appendix D) and the tool kit (Appendix E).

Student Assessment and Statistical Evaluation

We distributed pre- and postsession surveys (Appendix F) to assess students' self-reported comfort on several variables, including identifying HT patients, discussing HT with patients, and providing TIC. We developed the pre- and postsession survey questions based on the learning objectives to assess the success of the session; we did not use outside resources when creating the survey questions. To ensure anonymity, we had students assign themselves a unique identifier and instructed them to use this same identifier for both pre- and postsurveys. The surveys consisted of seven questions that assessed confidence on a 5-point Likert scale (1 = *very uncomfortable,* 5 = *very comfortable*). We also included a free-response question asking students if they had any previous education on HT as well as a yes/no question asking if they thought the session should be part of the required medical school curriculum. A paired t test was used to compare pre- and postsession survey responses.

Results

One hundred twenty-seven fourth-year medical students at Rush Medical College attended the session. Of these students, 95 (75%) completed both pre- and postsession surveys and were matched with a unique identifier. We compared the matched pre- and postsession survey scores. The data indicated a statistically significant difference in student comfort levels with identifying trafficked patients, discussing HT with patients, and the ability to provide TIC (Table 1).

At the end of the postsession survey, we asked students what they liked most about the session and what they felt were opportunities for improvement. Respondents indicated that the strengths of the training included the relevance of the topic for them as future physicians, the multiple learning modalities utilized during the session, and the incorporation of a variety of expert speakers. As for suggestions for improvement, several students indicated they wished the content had been discussed earlier in the medical school curriculum and in greater depth; another suggestion that stood out was to add more case vignettes for students to work through and discuss (Table 2).

Table 1. Comparison of Pre- and Postsession Survey Results (N = 95)

**Survey Item**[a]	**Presurvey *M* (*SD*)**	**Postsurvey *M* (*SD*)**	***p***
I am aware of the issue of human trafficking in the United States.	3.2 (1.0)	4.3 (0.7)	<.001
I understand what could make someone vulnerable to being trafficked.	3.2 (1.0)	4.5 (0.6)	<.001
I feel comfortable with what screening questions to ask to facilitate a disclosure and how to ask them sensitively.	2.3 (1.0)	4.2 (0.6)	<.001
I am confident in my ability to respond in a trauma-informed, patient-centered way to disclosures of human trafficking.	2.7 (1.1)	4.1 (0.6)	<.001
I feel able to safety plan with a patient who is not yet ready to leave their human trafficking situation.	2.1 (1.0)	3.9 (0.7)	<.001
I know what situations call for mandated reporting of human trafficking.	2.2 (0.9)	4.3 (0.7)	<.001
I know who to call should my patient be ready to leave their human trafficking situation.	2.0 (1.0)	4.0 (0.8)	<.001
I am aware of the red flags of human trafficking.	2.7 (1.0)	4.6 (0.6)	<.001

[a]Rated on a 5-point Likert scale (1 = *very uncomfortable,* 5 = *very comfortable*).

Copyright © 2023 Brennan et al. This is an open-access publication distributed under the terms of the Creative Commons Attribution license.

Table 2. Student Feedback

Questions and Themes	Representative Quotes
What did you like about this training?	
Content relevance	"Very relevant information that is not talked about enough in medical school training."
	"Human trafficking is an important topic that isn't covered as much as it should be."
	"I thought it was incredibly important and a very significant topic to go over."
Patient vignette	"I liked working through the patient case and actually trying to figure out what to say to the patient."
	"The questions on the word document really made us think."
	"I like that the vignette went step by step of encountering a patient who may be trafficked so that we can think about what we would do at each step from recognizing the situation to creating a safety plan."
Small-group discussion	"I liked hearing how others would respond to patients with trauma-informed tools in mind, and I found that helpful when thinking of what I would say in my own words."
Didactic lectures	"The lectures at the beginning were extremely helpful to get some of the details of how to approach these difficult situations."
Expert speakers	"There was a wide variety of expertise among the facilitators to provide diverse and unique perspectives."
	"I liked hearing the perspectives of people who are trained and do this work."
What are the opportunities for improvement for this training?	
Having the session earlier in training	"Would be helpful to have this at the end of 3rd year before we rotate through the emergency department."
	"Having it earlier in training to have kept it in mind on earlier clinical rotations would have been great."
More vignettes	"Maybe more cases with a little more variety to think through different human trafficking scenarios."

The 98 students who filled out the postsurvey had an average satisfaction rating of 4.6 out of 5. When asked how relevant they thought this session was to their future role as a resident physician, students rated it 4.8 out of 5 in terms of the level of importance.

Discussion

Our session aimed to equip students with the knowledge to broach the problem of HT, and our results demonstrated the session's efficacy in doing so. Among those who completed both the pre- and postsession surveys, there was a statistically significant increase in comfort levels across all measured items: comfort in identifying HT survivors, asking HT screening questions, discussing HT with patients, safety planning with patients, and providing TIC.

We developed this session to equip medical students with the knowledge necessary to identify survivors of HT and provide patient-centered TIC in a clinical setting. Students' feedback identified several factors that made our session unique and contributed to its success. A major strength of our training was its length. It lasted for 2 hours to maintain student attention and engagement. It also utilized a variety of modalities for learning, 25% didactic lecture and 75% interactive clinical vignette material, allowing students to absorb and apply valuable information. Keeping the didactic lecture to 25% of the content was essential in that it allowed the students to brainstorm in small and large groups about what they would do in the scenario proposed by the clinical vignette. Another strength was the incorporation of several experts in the field, all with extensive experience in both care for HT survivors and implementation of TIC in a variety of settings.

Most importantly, our session is replicable. It can be adapted for the education of any graduate student or health care worker, not just medical students. The session was hosted on Zoom due to the high number of COVID-19 cases at the time; however, it is adaptable for in-person use as well. Some of the benefits of Zoom included ease in scheduling guest speakers, the ability to host more students, and the ease and flexibility of allowing students to care for themselves in moments of feeling uneasy or triggered, given the sensitivity of the subject. The benefits of an in-person session may include greater student engagement and face-to-face interaction.

One recurring piece of feedback we received was that students wished the session content had been covered earlier in their medical school education. Our target audience was fourth-year medical students transitioning to their respective residency programs. We targeted this group because the session touched

Copyright © 2023 Brennan et al. This is an open-access publication distributed under the terms of the Creative Commons Attribution license.

on advanced communication and TIC concepts. These students each had at least 2 years of clinical experience, which we believed would strengthen the quality of small- and large-group discussions on this topic. However, we acknowledge that the introduction of basic TIC concepts could certainly be initiated earlier in training. We are considering a longitudinal curriculum in which basic TIC concepts are introduced in preclerkship years along with a more advanced session that targets students with clinical clerkship experience.

There were several limitations to our project. First, the group discussion portion of the session centered around a single case scenario. No HT survivor is the same, and thus, it could be helpful in future iterations to include a variety of cases to demonstrate the variable presentations of HT and avoid stereotyping survivors as one possible presentation. We have included a document with five supplementary case scenarios that can be drawn from in future iterations of this training (Appendix G).

Another limitation was that the session was held virtually, which may have limited the participation of students despite our asking all of them to turn their cameras on to maximize engagement. In the future, we plan to hold in-person sessions and compare the results with those of the virtual sessions. We utilized small-group discussion to encourage equitable participation; however, those who were more social and interested in the topic were likely more engaged than others. Additionally, not all medical students who underwent the training completed both the pre- and postsurveys, and there are no data on the retention of this material or on students' changes in behavior while interacting with patients in a clinical setting. In future iterations, we plan to resurvey students several weeks after the session to assess retention of the material and ask if they have since had experience identifying or interacting with HT survivors during clinical clerkships. Finally, although we consider the use of experts in HT and TIC as a strength of our resource, we do realize that in some situations it will not be feasible to recruit experts in these domains. We have included reference materials for these topics in the facilitation guide (Appendix B) to help prepare individuals who do not have a background in HT or TIC.

Given the statistically significant results, we are planning to implement HT sessions in the curricula of the many health care graduate programs at Rush University and to provide data on these trainings. Additionally, we intend to develop a skills-based assessment in the future. We are advocating to improve health care worker awareness and impact by sharing these training results with other educational institutions. We hope that introducing the topic of HT and the principles on how to best care for these patients early in training can have a significant impact on this underserved population.

Appendices

A. Didactic Lecture.pptx
B. Facilitation Guide.docx
C. Student Worksheet Without Answers.docx
D. Student Worksheet With Suggested Answers.docx
E. Tool Kit.docx
F. Pre- and Postsession Survey Questions.docx
G. Extra Scenarios.docx

All appendices are peer reviewed as integral parts of the Original Publication.

EmmaRose F. Brennan: Third-Year Medical Student, Rush Medical College

Artemis Markopoulos, MA: Second-Year Medical Student, Rush Medical College; ORCID: https://orcid.org/0000-0002-4925-5245

Jaclyn Rodriguez, RN, SANE-A, SANE-P: Sexual Assault Nurse Examiner (SANE) Coordinator, Office of the Illinois Attorney General

Neeral K. Sheth, DO: Director, Medical Education in Psychiatry, and Assistant Professor, Department of Psychiatry and Behavioral Sciences, Rush Medical College

Nupur Shah, DO: Faculty Physician, Department of Emergency Medicine, Rush University Medical Center

Acknowledgments
We would like to give special recognition to Vinodinee Dissanayake, MD, MPH; Shivani Thanawala, MD; Kaitlynn Tracy, MD; Shivali Patel, MD; and the Rush University Medical Center Emergency Department Team.

Disclosures
None to report.

Funding/Support
None to report.

Ethical Approval
Reported as not applicable.

References

1. Human trafficking. U.S. Department of Justice. Accessed January 11, 2023. https://www.justice.gov/humantrafficking

Copyright © 2023 Brennan et al. This is an open-access publication distributed under the terms of the Creative Commons Attribution license.

2. Office to Monitor and Combat Trafficking in Persons. *Trafficking in Persons Report*. 20th ed. U.S. Department of State; 2020. Accessed January 11, 2023. https://www.state.gov/wp-content/uploads/2020/06/2020-TIP-Report-Complete-062420-FINAL.pdf
3. Lederer LJ, Wetzel CA. The health consequences of sex trafficking and their implications for identifying victims in healthcare facilities. *Ann Health Law.* 2014;23(1):61-91. https://lawecommons.luc.edu/annals/vol23/iss1/5/
4. Frey LM, Middleton J, Gattis MN, Fulginiti A. Suicidal ideation and behavior among youth victims of sex trafficking in Kentuckiana. *Crisis*. 2019;40(4):240-248. https://doi.org/10.1027/0227-5910/a000557
5. Oram S, Stöckl H, Busza J, Howard LM, Zimmerman C. Prevalence and risk of violence and the physical, mental, and sexual health problems associated with human trafficking: systematic review. *PLoS Med*. 2012;9(5):e1001224. https://doi.org/10.1371/journal.pmed.1001224
6. Substance Abuse and Mental Health Services Administration. *SAMHSA's Concept of Trauma and Guidance for a Trauma-Informed Approach*. Substance Abuse and Mental Health Services Administration; 2014. HHS publication (SMA) 14-4884. Accessed January 11, 2023. https://ncsacw.acf.hhs.gov/userfiles/files/SAMHSA_Trauma.pdf
7. Garg A, Panda P, Malay S, Rose JA. A human trafficking educational program and point-of-care reference tool for pediatric residents. *MedEdPORTAL*. 2021;17:11179. https://doi.org/10.15766/mep_2374-8265.11179
8. Weber LC, Ortega JC, Bastea S, Robitz RA, Mumma BE. Women Leading Healthy Change: a reciprocal learning experience for women in the sex trade and medical students. *MedEdPORTAL*. 2021;17:11154. https://doi.org/10.15766/mep_2374-8265.11154
9. Song AY, Poythress EL, Bocchini CE, Kass JS. Reorienting orientation: introducing the social determinants of health to first-year medical students. *MedEdPORTAL*. 2018;14:10752. https://doi.org/10.15766/mep_2374-8265.10752
10. Gore DJ, Prusky M, Solomon CJE, et al. Creation of a medical student training to improve comfort providing trauma-informed care to sexual assault survivors. *MedEdPORTAL*. 2021;17:11140. https://doi.org/10.15766/mep_2374-8265.11140
11. Chokshi B, Walsh K, Dooley D, Falusi O, Deyton L, Beers L. Teaching trauma-informed care: a symposium for medical students. *MedEdPORTAL*. 2020;16:11061. https://doi.org/10.15766/mep_2374-8265.11061
12. Brown T, Mehta PK, Berman S, et al. A trauma-informed approach to the medical history: teaching trauma-informed communication skills to first-year medical and dental students. *MedEdPORTAL*. 2021;17:11160. https://doi.org/10.15766/mep_2374-8265.11160
13. Elisseou S, Puranam S, Nandi M. A novel, trauma-informed physical examination curriculum for first-year medical students. *MedEdPORTAL*. 2019;15:10799. https://doi.org/10.15766/mep_2374-8265.10799
14. Implicit bias. National Institutes of Health. Updated June 3, 2022. Accessed January 11, 2023. https://diversity.nih.gov/sociocultural-factors/implicit-bias
15. Federal law. National Human Trafficking Hotline. Accessed January 11, 2023. https://humantraffickinghotline.org/what-human-trafficking/federal-law
16. *On-Ramps, Intersections, and Exit Routes: A Roadmap for Systems and Industries to Prevent and Disrupt Human Trafficking*. Polaris; 2018. Accessed January 11, 2023. https://polarisproject.org/wp-content/uploads/2018/08/A-Roadmap-for-Systems-and-Industries-to-Prevent-and-Disrupt-Human-Trafficking.pdf
17. Murphy LT. *Labor and Sex Trafficking Among Homeless Youth: A Ten-City Study—Executive Summary*. Loyola University; 2016. Accessed January 11, 2023. https://www.covenanthouse.org/sites/default/files/inline-files/Loyola%20Multi-City%20Executive%20Summary%20FINAL.pdf
18. *Global Estimates of Modern Slavery: Forced Labour and Forced Marriage*. International Labour Office; 2017. Accessed January 11, 2023. https://www.ilo.org/wcmsp5/groups/public/—dgreports/—dcomm/documents/publication/wcms_575479.pdf

Received: May 31, 2022
Accepted: November 23, 2022
Published: March 14, 2023

Copyright © 2023 Brennan et al. This is an open-access publication distributed under the terms of the Creative Commons Attribution license.
